# Rydberg-State Double-Well Potentials of Van der Waals Molecules

**DOI:** 10.3390/molecules29194657

**Published:** 2024-09-30

**Authors:** Tomasz Urbańczyk, Andrzej Kędziorski, Marek Krośnicki, Jarosław Koperski

**Affiliations:** 1 Smoluchowski Institute of Physics, Faculty of Physics, Astronomy and Applied Computer Science, Jagiellonian University, Łojasiewicza 11, 30-348 Kraków, Poland; tomek.urbanczyk@uj.edu.pl; 2 Institute of Physics, Faculty of Physics, Astronomy and Informatics, Nicolaus Copernicus University, Grudziądzka 5/7, 87-100 Toruń, Poland; andrzej.kedziorski@fizyka.umk.pl; 3 Institute of Theoretical Physics and Astrophysics, Faculty of Mathematics, Physics and Informatics, University of Gdansk, Wita Stwosza 57, 80-308 Gdańsk, Poland; marek.krosnicki@ug.edu.pl

**Keywords:** van der Waals molecule, Rydberg electronic state, double-well potential, ab initio calculations, vibrational energy structure, rotational energy structure

## Abstract

Recent progress in studies of Rydberg double-well electronic energy states of MeNg (Me = 12-group atom, Ng = noble gas atom) van der Waals (vdW) molecules is presented and analysed. The presentation covers approaches in experimental studies as well as ab initio-calculations of potential energy curves (PECs). The analysis is shown in a broader context of Rydberg states of hetero- and homo-diatomic molecules with PECs possessing complex ‘exotic’ structure. Laser induced fluorescence (LIF) excitation spectra and dispersed emission spectra employed in the spectroscopical characterization of Rydberg states are presented on the background of the diverse spectroscopic methods for their investigations such as laser vaporization–optical resonance (LV-OR), pump-and-probe methods, and polarization labelling spectroscopy. Important and current state-of-the-art applications of Rydberg states with irregular potentials in photoassociation (PA), vibrational and rotational cooling, molecular clocks, frequency standards, and molecular wave-packet interferometry are highlighted.

## 1. Introduction—Double-Well Structure of Rydberg Potential Energy Curves

**Figure 1:**
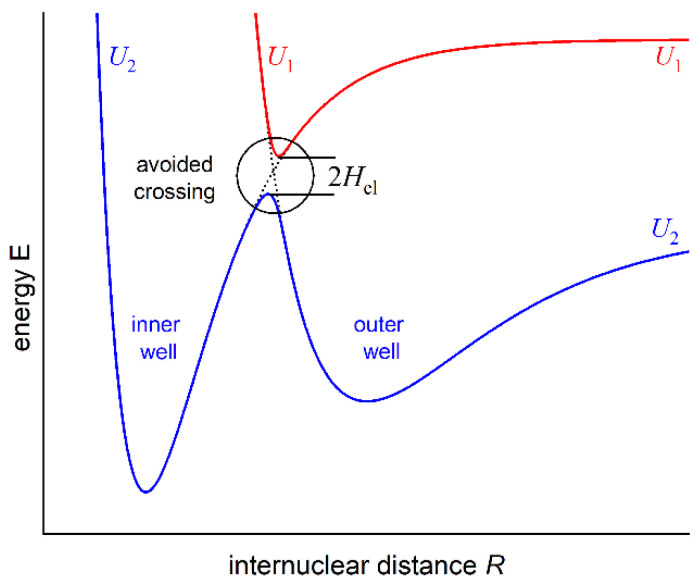
Illustration of the anti-crossing (avoided crossing) of the *U*_1_ and *U*_2_ potential energy curves that causes the formation of a potential energy barrier in the *U*_2_ separating two, inner and outer, potential wells.

As formulated by von Neumann and Wigner, the double-well (or double-minimum) structure of an interatomic potential energy curve (PEC) may have its origin in the so-called anti-crossing (or avoided crossing) phenomenon that occurs for two molecular potentials possessing the same symmetry properties [1]. For two anti-crossed potentials, the Born–Oppenheimer approximation breaks down, the adiabatic representation of electronic states takes over the diabatic one, and the potentials repel themselves (see Figure 1). This may cause the formation of a potential energy barrier that separates two potential wells (or potential minima). In order to facilitate the description, in 1985, Dressler postulated the so-called adiabaticity parameter [2] $\gamma =H_{el}/\hbar \omega_{U_{1}}$, where the $H_{el}$ electronic matrix element that couples the diabatic states and gives rise to a double-well state is compared with the vibrational constant $\omega_{U_{1}}$ in the upper of the two adiabatic states (description consistent with Figure 1). The strongly avoided (adiabatic) or weakly avoided (non-adiabatic) case dominates when γ≫1 or γ≪1, respectively.

The Rydberg character of an electronic energy state of a diatomic molecule may be manifested by the undulations of its PEC. This happens when one of the atoms is excited into its Rydberg state and the other atom experiences interactions with the Rydberg electron at relatively large internuclear distances (*R*), where the Rydberg state possesses consecutive lobes, see Refs. [3,4] and references therein.

As far as 12-group MeNg (Me = Zn, Cd, Hg and Ng = noble gas atom) van der Waals (vdW) molecules are concerned, early ab initio calculations of PECs of the lower-lying E3Σ1+[nsn+1s S13] and Σ0+1nsn+1s S10 (It is necessary to focus the Reader’s attention on the fact that throughout this article, the notation of the electronic energy states mostly follows that of the original references. Consequently, the following notations are present in this article: ΛΩ±2S+1 (or Λg,u±2S+1) and Ω±2S+1 (or Ωg,u±2S+1), corresponding to the description at the short and long *R*s or Hund’s cases (a) and (c), respectively; molecular quantum numbers: S—total spin, Λ, and Ω—projections on the internuclear axis of the total orbital angular momentum and the total angular momentum, respectively; *gerade* (g) or *ungerade* (u) symmetry for homoatomic molecules. For R→∞, the electronic energy states of diatomic molecules correlate with atomic asymptotes; in order to unify the notation, for homoatomic and heteroatomic (including MeNg) molecules with one excited atom, only the asymptote corresponding to the excited Me atom is shown; for homoatomic and heteroatomic molecules with both excited atoms, both asymptotes are given.) Rydberg states in ZnNg (n=4) [5], CdNg (n=5) [6,7], and HgNg (n=6) [8] showed that the potential barrier is not formed for Ng = He. It is formed in the neighbourhood of a single well for Ng = Ne and it is formed separating two potential wells for Ng = Ar, Kr, and Xe, manifesting a double-well character of the above-mentioned Rydberg state potentials (e.g., refer to Section 3.1). The findings were corroborated in a number of experiments performed for ZnAr [9], CdNe [10,11], CdAr [12,13,14,15,16,17,18], CdKr [14,16,19,20], HgNe [21,22,23], and HgAr [22,24,25], and recently, in more detailed ab initio studies performed for ZnAr [3] and CdAr [4] up to the (4s6s) and (5s7s) asymptotes, respectively, where the double-well character of the PECs was obtained for all the considered Σ Rydberg states.

However, the above-mentioned low-lying Rydberg states are well-separated from each other, excluding any (anti-) crossings of PECs. In 1986, it was suggested by Duval et al. [24] that the energy barrier of the lowest Rydberg state of the HgAr molecule may be correlated with the maximum of the Rydberg electron density; later, a similar claim was addressed to the lowest Rydberg state of HgNe [21]. In the 1990s, it was shown by Onda et al. [22,23] that the properties of the PECs derived from the OODR spectra of the Σ Rydberg states of HgNe and HgAr correlate with the Rydberg electron density. Inspired by the ab initio study of Rydberg states performed by Yiannopoulou et al. [26] for small diatomic molecules, extensive ab initio calculations [3,4] have supported the observation that the double-well structure (with the potential barrier present) of the $E^{3}\Sigma_{1}^{+}$ Rydberg state in ZnAr and CdAr, respectively, as the one possessing Σ symmetry, does not result from anti-crossing with other electronic states (note: $E^{3}\Sigma_{1}^{+}$ is degenerate with the ^3^$E^{3}\Sigma_{0}^{-}$ state correlating with the same $\left(4s{5s\;}^{3}S_{1}\right)$ or $\left(5s{6s\;}^{3}S_{1}\right)$ atomic asymptote in Zn or Cd, respectively). In this case, the formation of the potential barrier has a different origin. Similarly, as in the case of the so-called Rydberg molecules [27], it can be attributed to the low-energy scattering of the electron ($e^{-}$) being in the Rydberg state of the Zn or Cd atom from the ground-state Ng atom. This is a consequence of a model proposed by Fermi [28] and Omont [29] (see also Greene and collaborators [30,31]), who considered the interaction between Rydberg $e^{-}$ and the perturbing ground-state atom in a first approximation as low-energy *s*-wave (and *p*-wave [29]) scattering, leading to the energy shift that depends on *R* and is proportional to Rydberg $e^{-}$ density, i.e., the atomic Rydberg wavefunction squared, namely

(1) $$E_{s}\left(R\right)=2\pi A_{s}\left(k\right){\left|\Psi_{nlm}\left(R,0,0\right)\right|}^{2},$$

where $A_{s}\left(k\right)$ is the *s*-wave scattering length depending on the (classical) momentum k of a Rydberg electron in state $\Psi_{nlm}\left(R,\theta ,\varphi \right)$ [29]. The Rydberg molecule is assumed here to be on the *z*-axis, where θ and φ are zero. The model of the $e^{-}$–Ng interaction as $e^{-}$ scattering from Ng, is more accurate for highly excited Rydberg molecules [27,31]. As a consequence of this *s*-wave scattering, when at least one of the molecular constituents (e.g., Zn or Cd atom) is excited into the Rydberg state, PECs of the Rydberg Σ states exhibit undulations outside the inner potential well that reproduce the oscillations of the Rydberg $e^{-}$ density along the internuclear axis [3,4].

The description presented above is based on the formal division of the MeNg molecule in to three subsystems as follows: $Me^{+}$ cation, ground-state Ng atom, and Rydberg electron $e^{-}$. Outside the inner potential well, i.e., for sufficiently large *R*, the dominating contribution to the interaction energy between $Me^{+}$ and Ng is due to the charge-induced dipole interaction, whereas the $Me^{+}$–$e^{-}$ interaction is dominated by a Coulomb charge–charge one; the interaction between the Rydberg electron $e^{-}$ and the Ng atom is described by the generalized Fermi potential [28,29] considered above. In the case of the $Me^{+}$–Ng pair, the dispersion interaction is also present. However, this contribution to the interaction energy is smaller than in the case of the ground state of MeNg, mainly because of the smaller polarizability of the $Me^{+}$ cation in comparison with the Me atom. This interpretation is applicable for the $E^{3}\Sigma_{1}^{+}$ and Σ0+1 Rydberg states in a variety of MeNg molecules as suggested in Refs. [3,4,21,22,23,24] for ZnAr, CdAr, HgAr, and HgNe.

**Figure 2:**
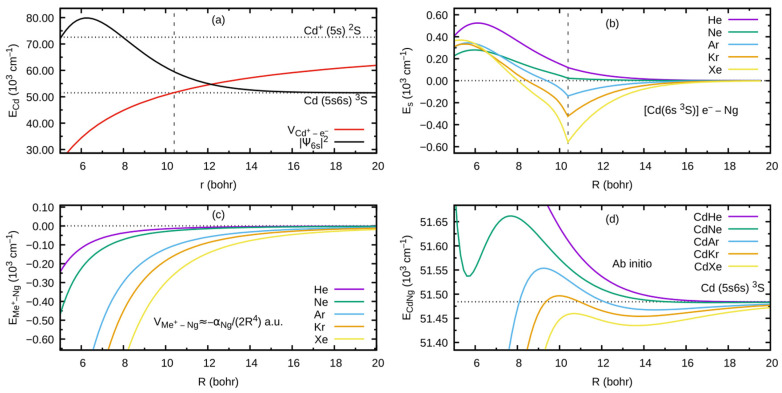
(**a**) ${Cd}^{+}$–$\;e^{-}$ interaction potential and square of the module of $\Psi_{6s}$ atomic orbital of Cd based on the results of ab initio calculations taken from Ref. [4], where values of the orbital are in arbitrary units (they do not correspond to the values on vertical axis); (**b**) energy shift $E_{s}(R)$ due to the Rydberg electron $e^{-}$–Ng interaction calculated using Equation (1) for the $E^{3}\Sigma_{1}^{+}$ Rydberg state of CdNg molecules; (**c**) charge ${Me}^{+}$–induced-dipole (Ng) interaction energy representing the dominating contribution to the long-range ${Me}^{+}$–Ng interaction; and (**d**) ab initio-calculated PECs of the $E^{3}\Sigma_{1}^{+}$ state of CdNg taken from Refs. [39,40]. The dashed vertical line in (**a**) and (**b**) indicates the classical range of the Rydberg 6*s* electron. Note: 1 a.u.(1 bohr) = 0.5292 Å. For details, see text.

As an example developed for this review, let us consider the formation of the outer well and the energy barrier in the $E^{3}\Sigma_{1}^{+}$ state of the CdNg molecule. In this case, the Cd atom in the lowest Rydberg state $\left(5s{6s\;}^{3}S\right)$ is perturbed by the appearance of the ground-state Ng atom. In Figure 2a, the interaction between the $Cd^{+}$ ion and the Rydberg electron $e^{-}$ (here: classical point charge $-\left|e\right|$) is presented along with the $\Psi_{6s}$ atomic orbital of Cd, where the values of the orbital are in arbitrary units and they do not correspond to the values provided on the vertical axis. The plots are based on results of ab initio calculations taken from Ref. [4], whereas the energy levels of the Cd atom are taken from [32]. From Figure 2a, the classically allowed region for the Rydberg electron can be established, where r<~10.4 bohr (~5.50 Å) (note that *R* is the distance between the Me and Ng atomic nuclei (i.e., internuclear distance) while *r* is the distance between the Rydberg electron and Me atomic nucleus), as well as the classical momentum of the Rydberg electron $k^{2}=2\left[E\left(5s{6s\;}^{3}S\right)-V(Cd^{+}-e^{-})\right]$ corresponding to this region. The interaction between the Rydberg electron $e^{-}$ and ground-state Ng atom is in first approximation determined by the Fermi potential [28,29], leading to the energy shift $E_{s}\left(R\right)$ given by Equation (1), presented in Figure 2b for the $\left(5s{6s\;}^{3}S\right)$ Cd state. Cusps on the border of the classical region in Figure 2b result from the fact that the scattering length $A_{s}\left(k\right)$ in Equation (1) is described semi-classically, where for the classically forbidden region, the value of $A_{s}\left(k\right)$ in the limit k⟶0 was adopted [27,28]. Low-energy electron scattering from Ng atom data for the generation of *E_s_*(*R*) in Figure 2b was taken from Refs. [33,34,35,36,37]. Figure 2c presents the charge $Me^{+}$-induced dipole (on Ng) interaction that dominates in the region of the large-enough *R* of the $Me^{+}$–Ng subsystem, where Ng static dipole polarizabilities $\alpha_{Ng}$, which determine the $Me^{+}$–Ng interaction, were taken from Ref. [38]. Figure 2d collects the $E^{3}\Sigma_{1}^{+}$-state PECs of CdNg molecules taken from Refs. [39,40] and shows the internuclear region around the outer wells and the energy barriers. In a first approximation, the sum of the $\left(5s{6s\;}^{3}S\right)$ Cd asymptote in Figure 2a and of the subsystem interactions $e^{-}$–Ng and ${Cd}^{+}$–Ng in Figure 2b,c should mimic the behavior of the actual PECs of the CdNg molecules in Figure 2d. It should be kept in mind that the simplified model of Rydberg molecules works well for highly excited states [27] and, at the same time, the lowest Rydberg state of CdNg molecules is considered here. Nonetheless, some intuitions derived from the simplified description may be picked up even in such a case [3,4]. It is seen in Figure 2b,d that the position of the energy barrier of the *E*^3^*Σ*_1_^+^ state may be ascribed to a situation in which the outside lobe of the Rydberg atomic orbital seen in Figure 2a is accompanied by the positive value of the scattering length $A_{s}\left(k\right)$ for a large enough momentum k, leading to positive energy shifts $E_{s}\left(R\right)$. Positions at the top of the energy barrier of the PECs of CdNg molecules are shifted with respect to the maximum of the square of the Rydberg atomic orbital towards larger distances because of the attractive ${Me}^{+}$–Ng interaction in Figure 2c. At the same time, the negative values of the scattering length $A_{s}\left(k\right)$ in the k⟶0 limit for Ng = Ar, Kr, Xe along with relatively large attractive ${Cd}^{+}$–Ng forces, see Figure 2b,c, results in the formation of the outer well seen in the ab initio PECs in Figure 2d. In the case of Ng = He, Ne, the positive values of the scattering length $A_{s}\left(k\right)$ and relatively weak attractive ${Cd}^{+}$–Ng forces lead to almost purely repulsive (the ab initio-calculated PECs of Rydberg states from Refs. [39,40] for CdHe and CdNe exhibit in fact very shallow outer wells of $\sim 1\;{cm}^{-1}$ depth with respect to the corresponding $\left(5s{6s\;}^{3}S\right)$ and $\left(5s{6s\;}^{3}S\right)$ atomic asymptotes) PECs outside the energy barrier. Thus, it is evident that the properties of the PECs of the lowest Rydberg state of CdNg may be qualitatively described within a simplified model of Rydberg molecules.

## 2. Motivation for the Study and Realistic Applications of the Results

Results gained in basic science research frequently result in practical applications and technological development. Technological achievements serve society and broadly understood human activities, including economic development, but also assist in pushing basic science concepts forward, thereby closing the circle between basic research and practical applications. This universal statement can be easily applied in the basic research reviewed here and devoted to acquiring knowledge on irregular double-well molecular potentials of Rydberg electronic states, leading to the description of complex interatomic interactions. To illustrate this, we will mention only few of the rich diversity of examples where knowledge on molecular potentials led to significant progress towards their applicability. We focus on the creation of entanglement between atoms—a step towards the concept of quantum computers, laser photoassociation, and invention of methods for vibrational and rotational cooling, leading to the creation of cold molecules in the ultimately coolest (υ=0,J=0) energy level, allowing, among others, the development of molecular clocks and frequency standards [41]. Also, we cannot leave out something that theorists appreciate greatly—impressive advances in ab initio calculation methods that subsequently allow us to credibly confront theoretical and experimental results.

### 2.1. Molecular Optical Clocks and Frequency Standards for Fundamental Tests

Frequency standards and optical clock transitions have been suggested as a tool for testing fundamental forces in search for New Physics beyond the Standard Model [42], for searching dark-matter [43,44,45,46,47] in the laboratory to explore possible variations in the fundamental constants of nature [43,47,48,49] and variations in the proton-to-electron mass ratio [50,51,52,53], and for searching and establishing constraints of the electron’s electric dipole moment [48,54,55,56,57].

Twelve-group Me closed-shell atoms have attracted attention as the possible candidates for optical lattice clocks based on Hg [48,58,59,60], Cd [60,61,62,63], and Zn [60,61] because of the presence of long-live atomic levels, ultra-narrow optical transitions, very small black-body radiation shift in the ^1^$S_{0}{\;-\;}^{3}P_{0}$ ‘clock transition’, and reduced susceptibility to blackbody radiation as compared with Sr- or Yb-based clocks. Consequently, they serve as promising alternatives to the currently operational Sr and Yb clocks [61,63].

This automatically directs one to the possibility of considering molecules as candidates for optical clocks and, indeed, there are examples of molecular clocks constructed using $I_{2}$ [64] and ${CH}_{4}$ [65] and proposals for clocks based on molecular ions $H_{2}^{+}$ and $HD^{+}$ as an active media [66,67]. So far, molecular clocks based on 12-group atoms are somewhat elusive. However, just recently, weakly bonding vdW diatomic molecules containing Zn or Cd and opened-shell alkali–metal (Li, Na, K, Rb, Cs, Fr) or alkaline–earth metal (Be, Mg, Ca, Sr, Ba, Ra) atoms—because of their considerable permanent electric dipole moments and high chemical reactivity—were considered within an ab initio approach for potential use in chemistry experiments and ultracold physics [68] that might lead to proposals for molecular optical clocks. Other homo-atomic weakly bound molecules, $Yb_{2}$ [69] and $Sr_{2}$ [70], were proposed to serve as suitable media to construct optical clocks based on optical Rabi frequency induced by magnetic coupling and long vibrational coherence driven by the off-resonant Raman process, respectively, or working as the THz lattice clock in $Sr_{2}$ based on pure molecular vibrations [71].

### 2.2. Experimental versus Ab Initio-Calculated Rydberg Molecular Potentials—Calculational Challenges

Frequently, a comparison of the results of ab initio-calculated molecular potentials with those from experimental studies shows large discrepancies for both ground- and excited-state potentials, including those of Rydberg states with complex, double-well, shapes. In majority of our studies on 12-group MeNg [11,12,13,15,16,17,19,20] and $Me_{2}$ [72,73] molecules, the discrepancies manifested themselves to varying degrees. The problem was closely analysed in recent articles on the calculations of the Rydberg-state potentials of CdAr [4] and ZnAr [3] molecules. In general, presently, exact ab initio calculations of many electron systems such as 12-group MeNg and $Me_{2}$ are impossible, where the main source of inaccuracy is due to deficiencies in the description of the electron correlation. Apart from the requirements for sufficient computer power, the requirements for the proper description of the excited states within ab initio calculations are the following: (1) an adequate atomic basis set optimized for electronic correlation and relativistic calculations that efficiently describes the considered states including electron correlation (and relativistic) effects and (2) a (quasi)-relativistic description starting from the reference function usually obtained with the single- or multiconfiguration self-consistent field (SCF) method, taking into account (3) the multireference character of the electronic state using, e.g., the complete active space SCF (CASSCF) method (usually in the case of excited electronic states), (4) a dynamic correlation as thorough as possible, and (5) spin-dependent relativistic effects (spin–orbit, spin–spin couplings). The dynamic electron correlation for the multi-reference CASSCF function is usually calculated with CAS second-order perturbation theory (CASPT2) or the multi-reference configuration interaction (MRCI) method. Alternatively, because of the fact that the ground state of MeNg molecules is dominated by a single-electron configuration, the excited states may be calculated with the equation-of-motion (EOM) method performed for the single-reference coupled cluster (CC) function of the ground state. If very accurate results are needed, one may consider the inclusion of hyperfine-structure interactions (if present), the effects of finite nuclear mass (beyond Born–Oppenheimer approximation), or QED effects.

More accurate ab initio calculations stimulated the requirements for more precise experiments investigating molecular energy structures with better spectral resolution. The stimulation works both ways and was exceptionally challenging for Rydberg electronic states.

### 2.3. Scheme for the Dissociation of Diatomic Molecules—Entanglement between Objects with Rest Masses

In 1995, Fry and coworkers published a proposal for the experimental realization of Bohm’s spin-1/2 particle version [74,75] of the Einstein–Podolski–Rosen (E-P-R) experiment [76] for two ^199^Hg atoms, each with nuclear spin I=1/2, that are produced in an entangled state with a total nuclear spin of zero [77]. It was proposed that the entanglement can be achieved by laser dissociation of the vibrationally and rotationally cold isotopologue of the ^199^Hg_2_ dimer produced in a supersonic expansion beam, using a spectroscopically selective stimulated Raman process. The measurement of nuclear spin correlations between the two ^199^Hg atoms in the entangled state is achieved by detecting the atoms using a spin-state-selective two-photon excitation–ionization scheme. In order to realize the idea, one needs to explore, theoretically and experimentally, the ro-vibrational energy structure of the electronic energy states involved in the process of selective dissociation, including subtle irregularities in the interatomic potentials. A proposal by Fry and coworkers followed an earlier suggestion for a test of so-called Bell inequalities [78]. The configuration of the electronic energy states in $Cd_{2}$ allowed the formulation of a similar proposal for two ^111^Cd atoms obtained by laser dissociation of the isotopologue of the ^111^Cd_2_ dimer [79], which is presently realized experimentally in our laboratory.

Except for 12-group ^199^Hg_2_ and ^111^Cd_2_ isotopologues, the E-P-R experiment and tests of Bell inequalities was also designed for the $Na_{2}$ molecule [80] and ^171^Yb_2_ isotopologue [81], both produced in supersonic beams. Some aspects of the two-atom entanglement phenomenon were experimentally realized for $Ca_{2}$ [82,83] and $H_{2}$ [84,85] molecules dissociated using laser and electron beams, respectively. A general discussion on the quantum-entanglement-related aspects of the dissociation process of homo- ($H_{2}$, ${He}_{2}$, ${Li}_{2}$, ${Cl}_{2}$) and heteronuclear (HCl) diatomic molecule experiments was reported by Esquivel and coworkers [86], where, in particular, the electronic entanglement during a dissociation process is shown to be associated, among others, with the spatial electronic density in the function of *R* and, consequently, to be dependent on the exact shape of the corresponding interatomic potential.

### 2.4. Photoassociation of Molecules with Double-Well Potentials—Cold Molecules from Cold Atoms

In different branches of atomic and molecular physics, there is rapidly growing interest in long-range forces acting between atoms interacting in a variety of traps and the photoassociation (PA) of cold molecules [41,87,88].

**Figure 3:**
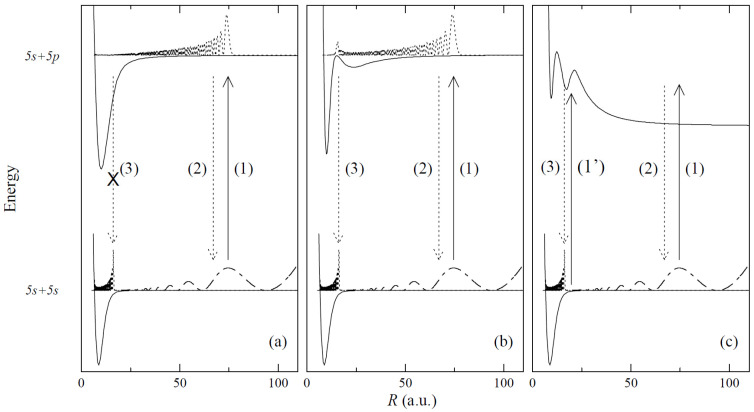
Photoassociation from the rubidium 5*s* ^2^S_1/2_ ground state asymptote [reaction (1) and (1’)] to (**a**) a typical long-range attractive potential; (**b**) a double-well potential, attractive at a long distance [e.g., $0_{g}^{-}$(5*p* ^2^P_3/2_)]; and (**c**) a double-well potential, repulsive at a long distance [e.g., $0_{g}^{+}$(5*p* ^2^P_3/2_)]. The system decays by spontaneous emission either back to the continuum [reaction (2)] or to a bound level of a lower electronic state [reaction (3)] [e.g., the $a^{3}\Sigma_{u}^{+}$(5*s* ^2^S_1/2_) state]. For case (**a**), reaction (3) is usually unlikely. Note: 1 a.u.(1 bohr) = 0.5292 Å. (from Ref. [89], under permission of EDP Sciences, Springer-Verlag).

The recent advances in laser cooling and optical trapping techniques, as well as one- and two-step PA processes of cold molecules, have been largely responsible for the renewed interest in studies of interatomic potentials including those with complex (e.g., double-well) structures, with distinct barriers present and specific behaviour in the long-range region of *R* [89] (see Figure 3). For a long time now, molecular energy states with double-well potentials have been considered as particularly suitable for PA experiments [90,91]. Knowledge of the complex shape of interatomic potentials (including higher-lying Rydberg potentials) facilitates an optimum design of PA schemes. Among the various available techniques, molecular spectroscopy of diatomic molecules has proved to be the most effective and precise way to obtain information about the interaction between atoms in the molecule being created, usually in a cold environment of an atomic trap.

It is important to emphasize here that knowledge on the interatomic potentials also allowed for designing unique schemes for PA processes. For example, using the $3^{1}\Sigma_{u}^{+}$(6*s* ^2^S_1/2_) double-well state in $Rb_{2}$, it was possible to propose its formation via PA and application in cold-physics experiments [92]. A proposal for the PA of ultra-long-range rotating Rydberg molecules was also recently reported [93]. The proposal specified different steps to calculate the wave functions and the transition matrix elements so they could be transferred to other Rydberg molecules involving different atomic species or molecular coupling cases.

### 2.5. Vibrational and Rotational Cooling of Diatomic Molecules

Exploration of electronic and ro-vibrational energy structures in molecules provides a source of information to elaborate schemes for the cooling of molecular degrees of freedom as follows: external—translation and internal—vibration and rotation [94,95]. The cooling process can be realized in a variety of atomic or molecular traps and, in the case of atomic traps, it usually follows a process of creating molecules (e.g., through PA, see Section 2.4). The internal cooling of molecules can also be realized in molecular beams (e.g., free-jet or supersonic), where vibrations and rotations are partly frozen out because of the properties of the expansion itself. With respect to the interatomic potentials involved in the process, including those of a complex character, it is important that for an efficient cooling process, an electronic transition with good properties, in terms of lifetime, coupling strength, laser accessibility, and losses of molecules from the cooling scheme, is chosen. Moreover, the cooling electronic transition should be as ‘closed’ as possible with the assumption that after excitation, spontaneous emission returns the largest possible fraction of the molecular population to the initial electronic state. Consequently, it imposes one of the favourable conditions: the position of the excited-state potential minimum ${(R}_{e}^{\prime }),$ which should be as close as possible to that of the ground-state potential ${(R}_{e}^{\prime \prime }$), i.e., $R_{e}^{\prime }-R_{e}^{\prime \prime }=∆R_{e}\approx 0$. The condition assures highly diagonal Franck–Condon (F-C) factors, i.e., domination of $\upsilon^{\prime }-\upsilon^{\prime \prime }=\Delta \upsilon =0$ transitions, where υ is a vibrational quantum number.

The above-mentioned conditions allowed for the proposal of theoretical schemes for cooling and experimentally realizing them for homoatomic (e.g., [81,94,96,97,98,99]) and heteroatomic (e.g., [94,100,101,102,103]) molecules. The caesium dimer $Cs_{2}$ was the first homoatomic molecule for which the cooling scheme was proposed. Its external and internal cooling in supersonic beams was based on the B1Πu(6p P1/22)−X1Σg+(6s S1/22) transition [96]. This was followed by experimental realization in a trap using the $B^{1}\Pi_{u},$ C1Πu(6p P3/22)−X1Σg+ transitions (with the $C^{1}\Pi_{u}$ state possessing a double-well structure) [94,97], reaching the absolute ground-state $\left(\upsilon^{\prime \prime }=0,\;J^{\prime \prime }=0\right)$ level [97], where J is a rotational quantum number. Proposals for the vibrational and rotational cooling of 12-group ^202^$Hg_{2}$ [98] and ^114^$Cd_{2}$ [81,99] were based on the $F^{3}0_{u}^{+}\left({6s6p\;}^{3}P_{1}\right)-X^{1}0_{g}^{+}$(6*s*^2^ ^1^S_0_) and $c^{3}1_{u}\left(5s{5p\;}^{3}P_{2}\right)-X^{1}0_{g}^{+}$(5*s*^2^ ^1^S_0_) transitions, respectively. The ^3^$0_{u}^{+}\left({6s6p\;}^{3}P_{1}\right)-X^{1}0_{g}^{+}$(6*s*^2^ ^1^S_0_) transition was proposed for cooling down the internal degrees of freedom in ^174^$Yb_{2}$ [81,99]. Among heteroatomic molecules, frequently possessing large permanent electric dipole moments, which is desirable in manipulation by means of static and/or time-dependent electric fields, the experimental realization of vibrational and rotational cooling was reported for kinetically cold NaCs in a magneto-optical trap [94,100] using $A^{1}\Sigma^{+},b^{3}\Pi$(3*s* ^2^S_1/2_ + 6*p* ^2^P_1/2,3/2_) − *X*^1^Σ^+^(6*s* ^2^S_1/2_) transitions and achieving vibrational cooling from $\upsilon^{\prime \prime }\geq 4$ to $\upsilon^{\prime \prime }=0$. Moreover, theoretical schemes for the direct vibrational and rotational cooling of TlCl [101], AgH, AgD [102], and ^24^$Mg^{35}Cl$ [103] were proposed by employing the $a^{3}\Pi_{0^{+}}$(6*p* ^2^P_1/2_ + 3*p*^5^ ^2^P_3/2_) − *X*^1^Σ_0^+^_, $A^{1}\Sigma^{+}$(5*p* ^2^P_1/2,3/2_ + 1*s* ^2^S_1/2_) − *X*^1^Σ^+^(5*s* ^2^S_1/2_ + 1*s* ^2^S_1/2_), and *A*^2^Π(3*s*^2^ ^1^S_0_ + 3*p*^5^ ^2^P_3/2_) − *X*^2^Σ^+^ transitions, respectively, as well as highly diagonal F-C factor patterns.

## 3. Optical–Optical Double Resonance (OODR) Method in Molecular Spectroscopy—Assessment and Main Advantages

### 3.1. OODR—Principle of the Method

This article describes, in a broader context of similar methods, molecular spectroscopy experiments in which molecules are sequentially excited using spatially overlapping two-laser beams. This sequential excitation method, called optical–optical double-resonance (OODR), is a special case of two-photon spectroscopy in which two visible or ultraviolet wavelength photons of different frequencies resonantly excite a molecule from an initial level (υ,J) to a final level ($\upsilon^{\prime },J^{\prime }$) via a real intermediate level ($\upsilon^{\prime \prime },J^{\prime \prime }$). The OODR process is also called double-optical resonance, step-by-step excitation, or stepwise, two-step, or two-colour excitation.

The OODR method makes the excitation of higher-lying molecular electronic energy states possible, to which excitation from the ground state using a one-step process is not possible as the energy of excitation is too high (deep UV or VUV) or the symmetry of the excited state forbids the direct excitation from the ground state. This way, OODR paves the way for investigating higher-lying Rydberg molecular states or, in the case of homoatomic molecules, for studying molecular states that possess the same symmetry as the ground state from which the excitation originates. As a good example from our laboratory is the spectroscopy of a *gerade* Rydberg energy state in $Cd_{2},$ while the ^3^$1_{g}(5s{6s\;}^{3}S_{1})$ state was excited from the $X^{1}0_{g}^{+}$(5*s*^2^ ^1^S_0_) ground state via the $b^{3}0_{u}^{+}({5s5p\;}^{3}P_{1})$ intermediate state [104].

**Figure 4:**
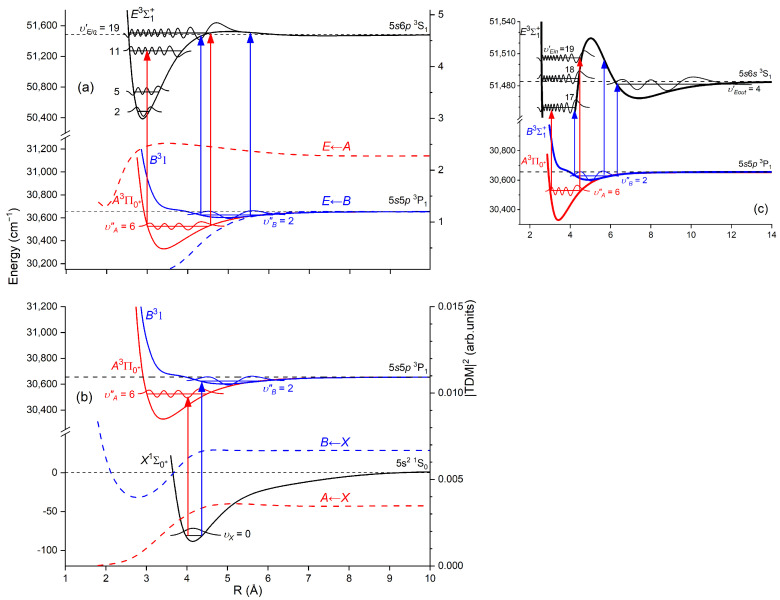
Illustration of optical–optical double resonance (OODR) method applied in molecular excitation. Potential energy curves (PECs) (solid lines) of the $X^{1}\Sigma_{0^{+}}$(5*s*^2^ ^1^S_0_), $A^{3}\Pi_{0^{+}}\left(5s{5p\;}^{3}P_{1}\right)$, $B^{3}1\left(5s{5p\;}^{3}P_{1}\right),$ and $E^{3}\Sigma_{1}^{+}\left(5s{6s\;}^{3}S_{1}\right)$ states in CdAr and electronic transition dipole moments squared ${\left|TDM\right|}^{2}(R)$ (dashed lines) are plotted according to the recent result published by Krośnicki et al. [4]. ${\left|TDM\right|}^{2}$ are plotted for (**a**) the first step of OODR: the $A^{3}\Pi_{0^{+}}\leftarrow X^{1}\Sigma_{0^{+}}$ and $B^{3}1\leftarrow X^{1}\Sigma_{0^{+}}$ transitions, and (**b**) the second step of OODR: the $E^{3}\Sigma_{1}^{+}\leftarrow A^{3}\Pi_{0^{+}}$ and $E^{3}\Sigma_{1}^{+}\leftarrow B^{3}1$ transitions. Examples of vibrational transitions (**a**) $\upsilon_{A,B}^{\prime \prime }\leftarrow \upsilon_{X}=0$ and (**b**) $\upsilon_{E}^{\prime }\leftarrow \upsilon_{A,B}^{\prime \prime }$ used in OODR are shown with vertical lines. The intensity of the vibrational transition depends on ${\left|TDM\right|}^{2}∼{\left|\int \psi_{el}^{\prime *}\mu_{el}\psi_{el}^{\prime \prime }d\tau_{el}\right|}^{2}$ along with so-called overlap integrals (**a**) $\int \psi_{\upsilon }\psi_{\upsilon^{\prime \prime }}dR$ and (**b**) $\int \psi_{\upsilon^{\prime \prime }}\psi_{\upsilon^{\prime }}dR$ for the first and second transition, respectively, where $\mu_{el}$ is an electric dipole operator, $\psi_{el}$ are electronic eigenfunctions, and $\psi_{\upsilon }$ are υ-level vibrational eigenfunctions (shown for each vibrational level). (**c**) Details of the second excitation. The ab initio-calculated height of the potential barrier is somewhat larger than that obtained from an experiment. Experimental positions of the $\upsilon_{E_{in}}^{\prime }$ and $\upsilon_{E_{out}}^{\prime }$ and $\upsilon_{A,B}^{\prime \prime }$ levels are also depicted.

Another advantage of OODR, which makes it an extremely useful spectroscopic tool, is a possibility to adequately choose an intermediate state in order to excite different parts of the final-state potential. This approach was employed in studies of 12-group-MeNg molecules including HgNg [21,22,23,24,25] and CdNg [10,11,12,13,14,15,16,17,105]. Figure 4 illustrates the OODR method using an example of the $E^{3}\Sigma_{1}^{+}\left(5s{6s\;}^{3}S_{1}\right)\leftarrow B^{3}1\left(5s{5p\;}^{3}P_{1}\right)\leftarrow X^{1}\Sigma_{0^{+}}$(5*s*^2^ ^1^S_0_) or $E^{3}\Sigma_{1}^{+}\left(5s{6s\;}^{3}S_{1}\right)\leftarrow A^{3}\Pi_{0^{+}}\left(5s{5p\;}^{3}P_{1}\right)\leftarrow X^{1}\Sigma_{0^{+}}$(5*s*^2^ ^1^S_0_) transition in CdAr. (According to the study presented in Ref. [4], the symmetry of the B state in CdAr changes with *R*. For a small *R*, i.e., in the region of the repulsive branch, state B possesses ‘pure’ Π symmetry, in the vicinity of the potential-well minimum, it possesses mostly Π symmetry, and at a larger *R*, the symmetry is Π+Σ ‘half-and-half’ mixed. This is similar for the remaining CdNg molecules [106]. Therefore, for the B state, the $B^{3}1$ notation will be kept throughout this review. This remark is also valid for ZnAr [3] and HgNg [8] molecules.) The molecule is excited in the first resonant optical transition from the $X^{1}\Sigma_{0^{+}}$ ground state to one of the $A^{3}\Pi_{0^{+}}$ or $B^{3}1$ states using the chosen $\upsilon_{A}^{\prime \prime }\leftarrow \upsilon_{X}=0$ or $\upsilon_{B}^{\prime \prime }\leftarrow \upsilon_{X}=0$ vibrational transition, respectively. The second resonant optical transition is a matter of choice. As the final $E^{3}\Sigma_{1}^{+}$ Rydberg state possesses a double-well structure, its potential can be probed using $\upsilon_{E}^{\prime }\leftarrow \upsilon_{A}^{\prime \prime }$ or $\upsilon_{E}^{\prime }\leftarrow \upsilon_{B}^{\prime \prime }$ transitions starting from either the $A^{3}\Pi_{0^{+}}$ or $B^{3}1$ state, respectively. Using the $A^{3}\Pi_{0^{+}}$ state as an intermediate, only the $E^{3}\Sigma_{1}^{+}$-state inner well ($E^{3}\Sigma_{1\;in}^{+}$) can be probed, whereas excitation via the $B^{3}1$ state offers an exploration of the $E^{3}\Sigma_{1}^{+}$-state outer well ($E^{3}\Sigma_{1\;out}^{+}$) along with the potential barrier exploring free←bound transitions (note: using excitation via the $B^{3}1$ state, a limited number of transitions to the $E^{3}\Sigma_{1\;in}^{+}$ near the dissociation limit can also be realized). The electronic transition dipole moments squared ${\left|TDM\right|}^{2}$ shown in Figure 4 constitute supporting information on which part of the $E^{3}\Sigma_{1}^{+}$-state PEC can be probed in the chosen transition.

### 3.2. Review of OODR Experiments in Diatomic Molecules

Since late 1970s and early 1980s, the OODR method has been employed in spectroscopy of higher-lying (Rydberg) electronic energy states of a variety of diatomic molecules, which were produced in quartz cells, heat-pipe ovens, specially designed furnaces, and molecular beams, including supersonic jets.

As the first, alkali diatomic homoatomic molecules have been investigated in heat-pipe ovens. Bernheim and coworkers spectroscopically studied the ${E^{1}\Sigma }_{g}^{+}$(3*s* ^2^S_1/2_) and ${F^{1}\Sigma }_{g}^{+},\;G^{1}\Pi_{g}$(2*p* ^2^P_1/2,3/2_ + 2*p* ^2^P_1/2,3/2_) Rydberg states in Li27 and provided molecular constants and quantum defects for ${{ns\sigma }^{1}\Sigma }_{g}^{+}$, ${{nd\sigma }^{1}\Sigma }_{g}^{+}$, and ${{nd\pi }^{1}\Pi }_{g}$ Rydberg series [107,108,109,110]. Xie and Field performed studies of the $2^{3}\Pi_{g}$(2*p* ^2^P_1/2,3/2_ + 2*p* ^2^P_1/2,3/2_), ${3^{3}\Sigma }_{g}^{+}$(3*p* ^2^P_1/2,3/2_), ${a^{3}\Sigma }_{u}^{+}$(2*s* ^2^S_1/2_), and $b^{3}\Pi_{u}$(2*p* ^2^P_1/2,3/2_>) states in other isotopologue, Li26 [111]. In Na_2_, the ^3^Π_g_(3*p* ^2^P^0^ + 3*p* ^2^P^0^) state was directly observed by Li and Field [112], whereas the ${5^{1}\Sigma }_{g}^{+}$(4*p* ^2^P^0^) and ${6^{1}\Sigma }_{g}^{+}$(5*s* ^2^S_1/2_) so-called ‘shelf’ states were investigated by Sanli et al. [113] and Saaranen et al. [114]. In 1999, the OODR spectroscopy of Li_2_ and Na_2_ Rydberg states was comprehensively characterized and discussed in a review article by Li and Lyyra [115]. For K239, four Rydberg states ${4^{3}\Sigma }_{g}^{+},3^{3}\Pi_{g}$(5*p* ^2^P^0^), $2^{3}\Delta_{g}$(4*d* ^2^D_3/2,5/2_), and $b^{3}\Pi_{u}$(4*p* ^2^P^0^) were studied by Kim et al. [116], while for Rb_2_, Arndt et al. observed and analysed the $3^{1}\Pi_{g}$(6*s* ^2^S_1/2_ + 6*p* ^2^P^0^) state [117] and the double-well of the ${6^{1}\Sigma }_{g}^{+}$(5*p* ^2^P^0^ + 5*p* ^2^P^0^) state [118]. Studies of Rydberg states in heteroatomic diatoms employing OODR were performed, among others, by Jabbour and Huennekens for the $6^{1}\Sigma^{+}$(5*p* ^2^P_1/2_ + 4*s* ^2^S_1/2_) state in NaK [119] and Chu et al. for the $C^{1}\Sigma^{+}$(4*s* ^2^S_1/2_ + 1*s* ^2^S_1/2_) double-well state in NaH [120], both in heat-pipe ovens; by Bernath and Field for the $E^{2}\Sigma^{+}$ and $E^{\prime 2}\Pi^{+}$ states in CaF [121]; by Ludwigs and Royen for the $G^{2}\Sigma^{+}$ state in BaCl [122], both produced in a Broida-type furnaces; and by Field et al. for the $i^{1}\Sigma^{+}$ and $a^{3}\Pi$ states in BaO [123], produced in a specially designed apparatus [124].

Many experiments, similar to those reported from our lab in this review, employing OODR combined with molecular beams have been performed in studies of Rydberg states, among others, by Donovan and coworkers, who investigated the ${E0}_{g}^{+}{(5s5p^{5}\;}^{3}P_{2})$ and ${f0}_{g}^{+}{(5s5p^{5}\;}^{3}P_{0})$ ion-pair states in I_2_ using a pulsed beam of iodine with He as a carrier gas [125], by Michalak and Zimmermann, who studied the ^2^Σ(5*s* ^2^S_1/2_), ^2^Σ(7*s* ^2^S_1/2_), ^2^Π_1/2_, ^2^Π_3/2_, $\Delta_{3/2}$, $\Delta_{5/2}$(5*d* ^2^D_3/2,5/2_), and $\Delta_{5/2}$(5*f* ^2^F_5/2,7/2_) states in the KAr vdW molecule expanded in a supersonic continuously working (cw) jet [126]; by Bouloufa et al., who investigated the $C^{1}\Sigma^{+}$(3*s* ^2^S_1/2_ + 1*s* ^2^S_1/2_) state in LiH produced in an effusive cw beam [127]; by Kleimeno et al., who studied the $1_{g}\left({2p^{5}4p}^{\prime }{\left[3/2,1/2>\right]}_{1}\right)$ and $0_{g}^{+}\left({2p^{5}4p}^{\prime }{\left[3/2>\right]}_{2}\right)$ states in the Ne_2_ vdW molecule expanded in a pulsed supersonic beam [128]; and by Sunahori et al., who investigated the $E^{4}\Pi {(3p\;}^{2}P_{1/2,3/2}+2{p^{2}\;}^{3}P_{0})$ state in the BC free radical produced in a discharge free jet pulsed expansion [129]. In all of the above OODR schemes, the Rydberg state excitation was realized from the electronic ground state via an adequately and carefully chosen intermediate state.

The family of vdW molecules described in this article refers to MeNg, where Me is a 12-group metal atom (Me = Hg, Cd, Zn) and Ng is a noble gas atom. In early experiments, the excitation of the Rydberg states in MeNg was realized using OODR and reported for HgNe [21,22,23], HgAr [22,24,25], CdNe [11], CdAr [17,105], and CdKr [20]. Rydberg energy states of other MeNg molecules have not been explored using OODR, but it is worthwhile to mention here that the laser vaporization–optical resonance (LV-OR) method (see Section 3.3.1) was employed in the case of ZnAr [9]; as an alternative to OODR, LV-OR was also used in an investigation of Rydberg states in CdAr [18].

Analysing those early experiments, one has to note that by employing OODR, a number of Rydberg electronic energy states has been reached. Among HgNg (Ng = Ne, Ar) molecules, the first one that was investigated is the $C^{3}\Sigma_{1}^{+}\left({6s7s\;}^{3}S_{1}\right)$ double-well triplet state in HgAr. (In fact, the inner and outer wells of the $C^{3}\Sigma_{1}^{+}$ double-well state in HgAr was assigned by Duval et al. [24] as C and D wells, respectively, in order to distinguish between excitation to or emission from these inner and outer wells properly. In fact, in their earlier study, the inner and outer wells were treated as separate C(Ω=1) and D(Ω=0) electronic energy states with the D state of unknown origin). The $C^{3}\Sigma_{1}^{+}$ state was excited from the ground state via the $A^{3}\Pi_{0^{+}}({6s6p\;}^{3}P_{1})$ or $B^{3}1({6s6p}^{3}P_{1})$ intermediate state and characterized using LIF excitation spectra [24]. The authors found the inner and outer potential wells to be 1430 ± 7 ${cm}^{-1}$ and 38 ± 7 ${cm}^{-1}$ deep, respectively. The same intermediates, $A^{3}\Pi_{0^{+}}$ and $B^{3}1$, were used to excite the triplet Rydberg series of the ^3^$\Sigma^{+}\left({6sns\;}^{3}S_{1},n=7–10\right)$ states in HgNe [21,22], the ^3^$\Sigma^{+}\left({6s8s\;}^{3}S_{1}\right)$ Rydberg state in HgAr [22], and the singlet Rydberg series of the ^1^$\Sigma^{+}\left(6s{ns\;}^{1}S_{0},n=7–9\right)$ states in HgNe [23]. Studies by Onda et al. [22,23] showed that for HgNe, a distinct potential barrier in ^3^$\Sigma^{+}\left(6s{ns\;}^{3}S_{1}\right)$ and $i^{1}\Sigma^{+}\left({6sns\;}^{1}S_{0}\right)$ occurs for n=7 and is accompanied with the inner well located (almost) entirely above the dissociation limit. For n=8, a relatively deep inner well $\left(D_{e}\geq 200\;{cm}^{-1}\right)$ appears along with a small energy barrier $\left(D_{e}\sim 10\;{cm}^{-1}at\;R=\sim 7Å\right)$ for the singlet state, whereas for the triplet state, the energy barrier is expected at a slightly larger R. For the ^3^$\Sigma^{+}\left({6s8s\;}^{3}S_{1}\right)$ state in HgAr, only the inner potential well was characterized and found to be 1602 ± 4 ${cm}^{-1}$ deep (the outer, shallower potential well was not located).

Early studies of CdNg (Ng = Ne, Ar, Kr) molecules, in which OODR was employed, were performed by Czajkowski et al. [105] and Koperski and Czajkowski [11,17,20]. For all of three molecules, the potential of the $E^{3}\Sigma_{1}^{+}\left({5s6s\;}^{3}S_{1}\right)$ state was investigated in CdNe [11], CdAr [17], and CdKr [20] using excitation from $X^{1}\Sigma_{0^{+}}$(5*s*^2^ ^1^S_0_) via the $A^{3}\Pi_{0^{+}}\left({5s5p\;}^{3}P_{1}\right)$ or $B^{3}1\left({5s5p\;}^{3}P_{1}\right)$ intermediate state, allowing first-time studies of different parts of the $E^{3}\Sigma_{1}^{+}$-state potential exploring bound←bound and free←bound transitions, but with limited spectral resolution. From those studies, it was concluded that the $E^{3}\Sigma_{1}^{+}$-state potential in CdNe, CdAr, and CdKr possesses a distinct potential barrier which, in CdAr and CdKr, separates inner (deep) and outer (shallow) potential wells (for the $E^{3}\Sigma_{1}^{+}\;$state in CdNe, evidence of the outer well was not found, and the potential barrier was located entirely above the ${(5s6s\;}^{3}S_{1})\;Cd$ asymptote). It was also concluded that the outer well in CdAr and CdKr may accommodate eight ($\upsilon^{\prime }$ = 0–7) and seven ($\upsilon^{\prime }$ = 0–6) vibrational levels, respectively.

### 3.3. Example of Methods Alternative to OODR

#### 3.3.1. Laser Vaporization–Optical Resonance (LV-OR)

In general, the laser vaporization–optical resonance (LV-OR) method relies on utilizing a vaporization laser pulse (commonly, the second or third harmonic of the 1064 nm Nd:YAG laser, the 690 nm Ti:Sa laser, or the 308 nm XeCl excimer laser) in the first step of the excitation. It is usually used to overcome obstacles associated with heating a metal sample in the reservoir before the expansion to ensure sufficient metal partial vapour pressure (typical values are 250–350 mbar) to produce molecules during the adiabatic expansion to the vacuum. For most metal elements, the heating temperature should exceed 1300 K, creating considerable difficulties. Laser vaporization itself produces a number of metal atoms in their metastable electronic states. For example, for ZnAr (n=4) [9] and CdAr (n=5) [18], during the expansion, metastable metal atoms form molecules with a carrier gas (argon) in electronic states, such as $a^{3}\Pi_{0^{-}}\left({nsnp\;}^{3}P_{0}\right)$ or $b^{3}\Pi_{2}\left({nsnp\;}^{3}P_{2}\right)$, which may serve as intermediate states in the LV-OR process. Consequently, characterization of the $E^{3}\Sigma^{+}({4s5s\;}^{3}S_{1})$ Rydberg state in ZnAr and the $e^{3}\Sigma^{+}({5s6s\;}^{3}S_{1})$ Rydberg state in CdAr was performed using supersonic expansion beams and LIF excitation spectra recorded using the $E^{3}\Sigma^{+}⟵a^{3}\Pi_{0^{-}}$, $b^{3}\Pi_{2}$ and $e^{3}\Sigma^{+}⟵b^{3}\Pi_{2}$ second-step transitions, respectively.

The problem with the LV-OR method is that it relies on a highly non-selective excitation process that is realized in the first step of the excitation (LV), which produces a considerable amount of intermediate states, such as $a^{3}\Pi_{0^{-}}\left({nsnp\;}^{3}P_{0}\right)$, $A^{3}\Pi_{0^{+}}$, $B^{3}1\left({nsnp\;}^{3}P_{1}\right),$ and $b^{3}\Pi_{2}$, ^3^$\Sigma_{1}^{+}$, ^3^$\Sigma_{0^{-}}^{+}\left({nsnp\;}^{3}P_{2}\right)$. Consequently, usually more than one intermediate state may participate in the second step of the excitation, causing difficulties in the proper interpretation of the LIF excitation spectra. A particular case can be found in the above-mentioned investigation of the $E^{3}\Sigma^{+}$ state in ZnAr (e.g., Figure 1 in Ref. [9]), where two distinct $\upsilon^{\prime }$-progressions to the $E^{3}\Sigma^{+}$ state are present. In the interpretation of the recorded spectrum, the authors assumed that ‘the two series of vibrational progressions correspond to transitions from $\upsilon^{\prime \prime }=0$ of different ^3^$\Pi_{\Omega }$ levels to the F-C favoured $\upsilon^{\prime }$ levels of the $E^{3}\Sigma^{+}$ upper state’ and the ^3^$\Pi_{\Omega }$-state potentials were represented by respective Morse functions. Unfortunately, in an analogous study of the $e^{3}\Sigma^{+}$ state in CdAr, no corresponding spectrum was presented [18].

As compared with OODR, another disadvantage associated with LV-OR is the fact that it makes it impossible to choose the most appropriate intermediate state in order to study different parts of the final-state interatomic potential.

Despite the above-mentioned disadvantages, the LV-OR method was also employed using supersonic expansion beams in studies of other MeNg and Me_2_ (Me = 2-, 11- or 13-group atom) molecules, for example, MgNg (Ng = Ne, Ar, Kr, Xe) [130,131,132,133,134], AgNg (Ng = Ar, Kr, Xe) [135], AuAr [136], AlNg (Ng = Ar, Kr, Xe) [137,138], InNg (Ng = Ar, Kr, Xe) [139,140], Be_2_ [141], Ba_2_ [142], and Ga_2_ [143].

#### 3.3.2. Laser Photoassociation and Excitation (Pump-and-Probe)

Another frequently used technique that allows for studying Rydberg electronic states in molecules, especially those without stable or very weakly bound ground electronic state, is the so-called pump-and-probe method. This method has been widely used in the investigation of 12-group homoatomic Hg_2_ [144,145] and Zn_2_ [146,147,148,149] dimers and heteroatomic HgZn [150,151,152,153] and HgCd [154] excimers in search of media for potential tuneable laser working in UV and based on vdW molecules and exciplexes. These unstable diatomic molecules have repulsive ground-state potentials exhibiting shallow vdW minima with depths in the range of 220–550 ${cm}^{-1}$ [155,156,157,158]. Consequently, at temperatures higher than 270–380 K, they cannot exists in their ground states, so excitation to their Rydberg states has to be re-arranged, creating a considerable population of their low-lying metastable states while maintaining their vapour partial pressure at the necessary level.

**Figure 5:**
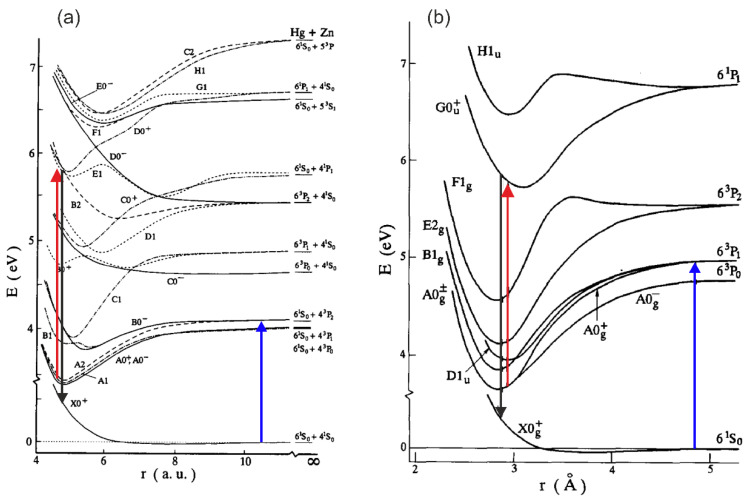
Diagram of interatomic potentials for (**a**) HgZn (from Ref. [153], under permission of Elsevier Science B.V.) and (**b**) Hg_2_ (from Ref. [72], under permission of Elsevier Science B.V.) showing the relevant pump (blue arrows), probe (red arrows), and LIF (black arrows) processes. Arrows are added to the original figure. Note: 1 a.u. = 0.5292 Å.

The pump-and-probe method employed in those studies relied on the irradiation of hot atomic vapour in a quartz cell by two laser pulses. The cell contains Hg, Zn, a Zn-Hg mixture, or a Cd-Hg amalgam to investigate Hg_2_, Zn_2_, HgZn, and HgCd, respectively. The cell is heated to 600–1100 K, depending on its content, to ensure a metal vapour partial pressure in the range of 600–2200 mbar. The first laser (pump) pulse is close to the resonance transition from the ^1^$S_{0}$ atomic ground state to the lowest-lying ^3^$P_{J}$ manifold. As a result of the process of three-body collisions ${2Me(}^{1}S_{0})+h\nu ⟶(Me_{2}^{*}\;or\;MeMe^{*})$ and the collisional relaxation that followed them, a $Me_{2}^{*}$ homoatomic or $MeMe^{*}$ heteroatomic metal dimer in a metastable $A0_{g}^{\pm }{(6s6p\;}^{3}P_{1})$, ^3^$\Pi_{g}({4s4p\;}^{3}P_{J})$, $A0^{\pm }{(6{s^{2}\;}^{1}S_{0}+4s4p\;}^{3}P_{J}),$ or ^3^${\Pi {(6s^{2}\;}^{1}S_{0}+5s5p\;}^{3}P_{J})$ state is created in $Hg_{2}$, ${Zn}_{2}$, HgZn, or HgCd, respectively (for HgZn [153] and Hg_2_ [72], see Figure 5). The consecutive laser (probe) pulse excites a corresponding molecule from the metastable ‘reservoir’ to the higher-lying Rydberg state, after which a resulting LIF is recorded while tuning the probe laser frequency. Several Rydberg states were investigated, providing spectroscopic characterization for the $H^{1}1_{u}{(6s6p\;}^{1}P_{1})$, $I^{1}0_{u}^{+}{(6s7s\;}^{1}S_{0})$, and ${J^{1}1_{u}(6s7s\;}^{3}S_{1})$ states in Hg_2_ [144,145], the ^3^$\Pi_{u}{(4s4d\;}^{3}D_{J})$ and ^3^$\Sigma_{u}^{+}$, ^3^$\Pi_{u}$, ^3^$\Sigma_{g}^{+}({4s4p\;}^{3}P_{J}+{4s4p\;}^{3}P_{J})$ states in Zn_2_ [146,147,148,149], the $E0^{-}$, $F1(6{s^{2}\;}^{1}S_{0}+4s{5s\;}^{3}S_{1})$, $F0^{-}({6{s^{2}\;}^{1}S_{0}+4s5p\;}^{3}P_{1})$, $D0^{+},\;G1{(6s6p\;}^{1}P_{1}+4{s^{2}\;}^{1}S_{0}),$ and $E1{(6s6p\;}^{3}P_{2}+4{s^{2}\;}^{1}S_{0})$ states in HgZn [150,151,152,153], and the $E1(6{s^{2}\;}^{1}S_{0}+{5s5p\;}^{1}P_{1})$ and $F1({{6s^{2}\;}^{1}S_{0}+5s6s\;}^{3}S_{1})$ states in HgCd [154], some of them having a complex double-well structure in their Rydberg-state potentials.

Instead of hot atomic ensemble in a cell or a heat-pipe oven, pump-and-probe experiments can be carried out for cold or ultracold atoms in atomic traps (magneto-optical, all-optical, etc.). Knowledge on the interatomic potentials, especially those with an ‘exotic’ irregular structure, thus enabling the PA of cold or ultracold homo- and heteroatomic diatomic molecules, is highly required. The formation of cold $Cs_{2}$ through PA in the $0_{g}^{-}({6p\;}^{2}P_{3/2})$ double-well excited state [159,160] and in the ground state through PA in the $1_{u}({6p\;}^{2}P_{3/2})$ long-range state [160,161] were reported. Also, giant, so-called Rydberg macrodimers, i.e., 1 μm sized cold $Cs_{2}$ molecules correlating with $nP_{3/2}(n+1)S_{1/2}$ dissociation asymptotes (n=43, 44) was produced from two Cs Rydberg atoms [162]. Cold $K_{2}$ molecules were formed through PA in the ${B^{1}\Pi }_{u}({4p\;}^{2}P_{J})$ state with a potential barrier [163] and in the ground state through two-photon PA in the ${5^{1}\Pi }_{u}({4d\;}^{2}D_{J})$ or ${6^{1}\Pi }_{u}({4f\;}^{2}F_{J})$ state the via ${1^{1}\Pi }_{g}({4p\;}^{2}P_{J})$ state [164]. The formation of cold ^85^$Rb_{2}$ through the $\upsilon^{\prime \prime }=39$ level of the $a^{3}\Sigma_{u}^{+}({5s\;}^{2}S_{1/2})$ state short-range PA to the $1^{3}\Pi_{g,\Omega =1}({5p\;}^{2}P_{3/2})$ state with a potential barrier [165] was performed. Among heteroatomic molecules, the formation of ultracold dipolar LiCs in the lowest ro-vibrational levels $\left(\upsilon^{\prime \prime }=0,\;J^{\prime \prime }=0\right)$ by PA into the $B^{1}\Pi ({2s\;}^{2}S_{1/2}+{6p}^{2}P_{3/2})$ state and decay to the $X^{1}\Sigma^{+}({2s\;}^{2}S_{1/2}+{6s\;}^{2}S_{1/2})$ state was reported [166]. In each of the above cases, when cold molecules were formed in one of their excited electronic state, PA was followed by an ionization using a carefully chosen molecule ⟶ molecular ion electronic transition using tuneable laser radiation. This allowed for a precise vibrational spectroscopy of very dense $\upsilon^{\prime }$ levels close to the dissociation limit in a long range of *R*.

#### 3.3.3. Polarization Labelling Spectroscopy

**Figure 6:**
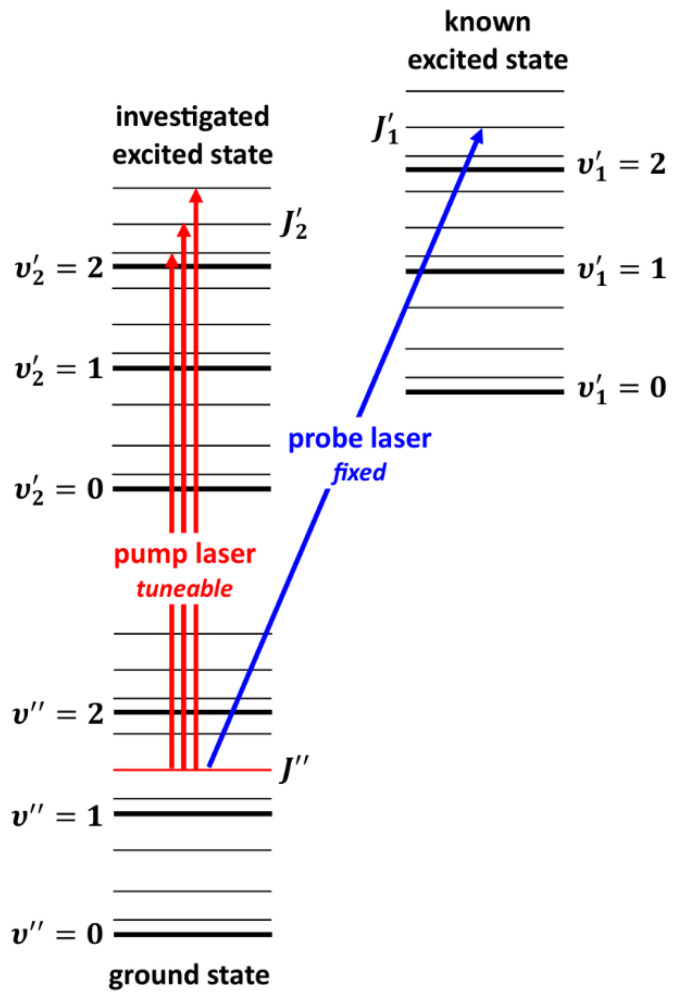
The ‘V-scheme’ of polarization labelling spectroscopy (see the text for details).

A very interesting alternative to OODR is the polarization labelling spectroscopy (PLS) method [167], which allows for simplifying spectra of diatomic molecules and investigating, among others, higher-lying molecular states, including those with a complex nature. A V-type optical double-resonance version of PLS relies on using two lasers, i.e., a fixed-frequency probe laser and a tuneable-frequency pump laser (see Figure 6). The frequency of the linearly polarized, weak probe laser is set in resonance with known ro-vibrational molecular transitions $\left(\upsilon_{1}^{\prime },J_{1}^{\prime })⟵(\upsilon^{\prime \prime },J^{\prime \prime }\right)$. The frequency of circularly or linearly polarized pump laser is tuned across the studied band system. At the frequencies at which the transition induced by the pump laser $\left(\upsilon_{2}^{\prime },J_{2}^{\prime })⟵(\upsilon^{\prime \prime },J^{\prime \prime }\right)$ shares the same lower level $\left(\upsilon^{\prime \prime },J^{\prime \prime }\right)$ with the probe laser transition, the probe laser beam changes its polarization. The change is detected with a set of crossed polarizers placed in the path of the probe laser beam on both sides of the molecular sample. Tuning the pump laser frequency over the excited-state $\upsilon_{2}^{\prime }$-progression $\left(\upsilon_{2}^{\prime },J_{2}^{\prime }=J_{1}^{\prime }\pm 1\right)$ provides the desired polarization labelling spectrum, simplified by the fact that it originates from a few ground-state levels with fixed and known $\left(\upsilon^{\prime \prime },J^{\prime \prime }\right)$ quantum numbers.

The PLS method has been employed to characterize the potentials of electronic states with double-minima in alkali metal homoatomic dimers, including ${{\;2}^{1}\Sigma }_{u}^{+}({ns\;}^{2}S_{1/2})$ in K_2_ (*n* = 5) [168] and in Na_2_ (*n* = 4) [169], $C^{1}\Pi_{u}({2p\;}^{2}P_{1/2,3/2})$ and ${{\;2}^{1}\Sigma }_{u}^{+}({3s\;}^{2}S_{1/2})$ in Li_2_ [170], ${{\;3}^{1}\Sigma }_{u}^{+}({6s\;}^{2}S_{1/2})$ in Rb_2_ [92], and $E{\left(3\right)}^{1}\Sigma_{u}^{+}({6s\;}^{2}S_{1/2}+{7s\;}^{2}S_{1/2})$ in Cs_2_ [171], and in heteroatomic dimers, including ${6^{1}\Sigma }^{+}({3s\;}^{2}S+{5p\;}^{2}P)$ in NaK [172], ${{\;6}^{1}\Sigma }^{+}$ in KCs $\left({5s\;}^{2}S_{1/2}+{6s\;}^{2}S_{1/2}\right)$ [173], and ${{\;6}^{1}\Sigma }^{+}({4s\;}^{2}S_{1/2}+{5d\;}^{2}D_{3/2,5/2})$ in NaRb [174], all produced in heat-pipe ovens.

## 4. Ab Initio-Calculated Potentials of MeNg Molecules—Early, Recent, and Future Approaches

The interatomic potentials of 12-group MeNg molecules (Me = Zn, Cd, Hg), including those of Rydberg electronic energy states, have been ab initio-calculated by a number of researchers, uncovering information on their PECs, respective absorption oscillator strengths $\left(f\right),$ and TDMs. The ab initio results were frequently confronted with the experimental outcomes, in some cases allowing successful experiments by the correct choice of excitation and/or emission spectral regions associated with the studied molecular electronic transitions.

The interatomic potentials of higher-lying electronic energy states of CdNg molecules including Rydberg states correlating with the $\left(5s6{s\;}^{3}S_{1}\right)$ and $\left(5s6{s\;}^{1}S_{0}\right)$ Cd asymptotes, and fs or TDMs, were theoretically studied by Czuchaj and Sienkiewicz [175] (Czuchaj and Sienkiewicz [175] reported PECs up to electronic Rydberg states correlating with the 5s5d 1D,D3 and 5s6p 3P,P1 asymptotes), Czuchaj and Stoll [6], Czuchaj et al. [7], Krośnicki and collaborators [39,40], Krośnicki et al. [4], and Li et al. [176], where in Refs. [4,176], Rydberg states were calculated up to the $\left(5s7{s\;}^{1}S_{0}\right)$ asymptote. The spin–orbit (s-o) effect was not included in Ref. [6] (in fact, in Ref. [6], the s-o interaction was included only for $\left(5s5{p\;}^{3}P_{J}\right)$ Cd levels using a semi-empirical approach (the s-o interaction was also included in the calculations presented in Ref. [175])). The calculations of Refs. [4,6,7,39,40] resulted in a double-well structure of the ^3^$\Sigma_{1}^{+}(5s{6s\;}^{3}S_{1})$ and ^1^$\Sigma_{0^{+}}({5s6s\;}^{1}S_{0})$-state potentials; it should be noted that in Ref. [4], all the considered Rydberg states of Σ symmetry up to the $\left(5s7{s\;}^{1}S_{0}\right)$ asymptote exhibited a double-well character.

As far as the methods of calculation are concerned, in Ref. [175], the semiempirical pseudopotential method was used, where the CdNg molecule was treated as two-electron system by placing ${Cd}^{2+}$ and total Ng into cores, while in Ref. [6], valence ab initio CASSCF/CASPT2 [177] calculations for CAS ${\left(5s5p6s\right)}^{2}$ were executed with ${Cd}^{2+}$ and ${Ng}^{8+}$ cores replaced by semi-empirical [178] and quasi-relativistic [179] pseudopotentials, respectively (CAS ${\left(5s5p6s\right)}^{2}$ is spanned by all many-electron functions in which two ‘active’ electrons are distributed on the active molecular orbitals of the predominant Cd 5s5p6s character. The remaining electrons occupy closed shells or are represented by pseudopotentials. Similar denotations of the CAS’s will be used hereafter). In Ref. [7], large scale valence ab initio CASSCF/CASPT2 [177] calculations were performed for CAS ${\left(5s5p6s6p\right)}^{2}$ with ${Cd}^{20+}$ and ${Ng}^{8+}$ cores simulated by energy-consistent effective-core pseudopotentials (ECPs), including scalar-relativistic effects and the s-o interaction within the valence shell [179,180]. Finally, in Refs. [39,40], calculations were performed with the CASSCF/CASPT2 level of theory [177] for CAS ${\left(5s5p6s6p\right)}^{2}$ with ${Cd}^{28+}$ and ${Ng}^{8+}$ cores replaced by ECPs [179,181]. The ground state PECs in Refs. [39,40] were calculated with the coupled-clusters method CCSD(T) [182,183]. It is noted that in Refs. [39,40], the internally contracted MRCI method [184,185] was used for a better description of the wavefunctions taken for the calculations of TDMs.

With respect to recent ab initio calculations of CdAr interatomic potentials, in 2019 Krośnicki et al. [4] reported on the relatively unexplored area of low-lying Rydberg states of vdW molecules. They performed first-time fully ab initio calculations on interatomic potentials of the Rydberg states of CdAr lying above the $(5s6{s\;}^{3}S_{1}$, ^1^$S_{0})$ asymptotes, reaching the $\left(5s6{p\;}^{3}P_{0,1,2}\right)$, $(5s5{d\;}^{1}D_{2}$, ^3^$S_{1,2,3})$, $\left(5s6{p\;}^{1}P_{1}\right),$ and $(5s7{s\;}^{3}S_{1}$, ^1^$S_{0})$ asymptotes. All the calculated PECs of the Rydberg states of Σ symmetry exhibited undulations, resulting in their double-well character (see also Section 1). The main calculations were performed with the restricted active space (RAS) SCF [186] method followed by RAS second-order perturbation theory (RASPT2) [187] for RAS (5*s*–1*e*//5*p*5*d*6*s*6*p*7*s*–1*h*) (here, the RAS (5*s*–1*e*//5*p*5*d*6*s*6*p*7*s*–1*h*) active space is spanned by the many-electron states in which only the single excitations are allowed from the doubly occupied molecular orbital of the predominant Cd 5*s* character into 5*p*5*d*6*s*6*p*7*s* counterparts. The remaining electrons occupy closed shells); the s-o interaction was included via the RAS state interaction (RASSI) method [188]. Wherever it was possible, the selected states were recalculated with the CASSCF/CASPT2 [189,190,191,192] and CCSD(T) [193] methods. In Ref. [4], the results of CCSD(T) calculations served as the benchmarks for the assessment of the RASSCF/RASPT2 counterparts.

In other recently performed calculations of CdNg potentials of Li et al. [176], only singlet potential curves of CdNg, up to the ^1^$\Sigma_{0^{+}}(5s{7s\;}^{1}S_{0})$ Rydberg state, and corresponding *f*s and TDMs were calculated. Surprisingly, no conclusion was provided in Ref. [176] about the double-well character of the ^1^$\Sigma_{0^{+}}$-Rydberg state potentials. Excitation energies in Ref. [176] were calculated with the EOM-CCSD method [194,195,196,197]. The corresponding PECs of the excited states were obtained by adding EOM-CCSD excitation energies to the ground-state PEC obtained with the CCSD(T) method, and with quasirelativistic energy-consistent small-core ECPs [181] along with large atom-centred basis sets and with midbond functions. Towards such results, experimental verification is highly needed. The respective experimental approach is planned in our laboratory in order to meet this demand.

To ensure a complete view of Rydberg-state interatomic potential calculations of 12-group MeNg molecules, those for ZnNg and HgNg are reviewed below.

Large scale quasirelativistic valence ab initio CASSCF/CASPT2 for CAS ${\left(4s4p5s5p\right)}^{2}$ calculations of the ^3^$\Sigma_{1}^{+}$ and ^1^$\Sigma_{0^{+}}$-Rydberg state potentials of ZnNg correlating with the $\left(4s5{s\;}^{3}S_{1}\right)$ and $\left(4s5{s\;}^{1}S_{0}\right)$ Zn asymptotes, respectively, were performed by Czuchaj et al. [5] and Krośnicki and collaborators [39,40]. In the calculations by Ref. [5], the ${Zn}^{20+}$ and ${Ng}^{8+}$ cores were replaced by ECP [179,198], which also accounted for scalar-relativistic effects and the s-o interaction. The result of the calculations showed that the ^3^$\Sigma_{1}^{+}$- and ^1^$\Sigma_{0^{+}}$-state potential curves in ZnAr, ZnKr, and ZnXe exhibit shallow second minima at larger *R*s. In Refs. [39,40], the calculations were made similar to those for CdNg described above with the ${Zn}^{20+}$ and ${Ng}^{8+}$ cores replaced by ECP and s-o and relativistic effects taken into account.

Recent ab initio calculations of ZnNg interatomic potentials were performed by Kędziorski et al. for ZnAr [3], Li et al. for ZnNg [199], and Li et al. for ZnHe [200]. In Ref. [3], PECs were ab initio-calculated up to the Rydberg state correlating with the $\left(4s6{s\;}^{1}S_{0}\right)$ Zn asymptote. The state-average (SA) CASSCF [189] was employed for CAS ${\left(4s4p4d5s5p6s\right)}^{2}$. Dynamic correlation effects were accounted for via multi-state (MS) CASPT2 [192]. The s-o interaction was included via the restricted active space state interaction method (RASSI-SO) (in Ref. [3], the detailed analysis of the accuracy of the results of ab initio calculations was performed with an emphasis on the important role of midbond functions). In Ref. [199], only singlet interatomic potentials of ZnNg, including the ^1^$\Sigma_{0^{+}}({4s6s\;}^{1}S_{0})$ Rydberg state, and corresponding TDMs, F-C factors*,* and spectroscopic constants were calculated with the EOM-CCSD method, as in Ref. [176], with no conclusion about the double-well character of the ^1^$\Sigma_{0^{+}}$ state. The calculations reported in Ref. [199] were executed using a method similar to that employed in Ref. [176]. Finally, in Ref. [200], ZnHe Rydberg state potentials were ab initio-calculated up to the $\left(4s5{p\;}^{1}P\right)$ asymptote using multireference configuration interaction plus the Davidson correction (MRCI+Q) method [184,201] on top of CASSCF calculations with CAS ${\left(Zn\;4s4p5s5p\;He\;1s\right)}^{4}$; s-o coupling was included.

Large-scale valence ab initio CASSCF/CASPT2 [177] for CAS ${\left(6s6p7s7p\right)}^{2}\;$calculations of the Rydberg ^3^$\Sigma_{1}^{+}$ and ^1^$\Sigma_{0^{+}}$-state potentials of HgNg correlating with the $\left(6s7{s\;}^{3}S_{1}\right)$ and $\left(6s7{s\;}^{1}S_{0}\right)$ Hg asymptotes, respectively, were performed by Czuchaj et al. [8]. In the calculations, the ${Hg}^{20+}$ and ${Ng}^{8+}$ cores were simulated by energy-consistent pseudopotentials [179,180], which also accounted for scalar-relativistic effects and s-o interactions. The result of the calculations showed that the ^3^$\Sigma_{1}^{+}$ and ^1^$\Sigma_{0^{+}}$-state potential curves in HgAr, HgKr, and HgXe exhibited shallow second minima at larger *R*s.

As mentioned above, the main source of the inaccuracies in ab initio calculations of excited states of MeNg molecules is due to deficiencies in the description of the electron correlation. Thus, a future approach for capturing electron correlations will probably be based on EOM-CC methods, where the level of the approximation should go beyond the CCSD one. The need for a higher level of CC approximation was shown, e.g., in Ref. [3], where the results of CCSD calculations were less accurate in comparison with MS-CASPT2 counterparts. The first works reporting EOM-CC calculations for excited states of MeNg molecules were published by Li et al. [176,199], where non-iterative triples were taken into account indirectly by adding EOM-CCSD excitation energies to the CCSD(T) total energies of the ground state of MeNg molecule. Detailed comparison of these recent results of ab initio calculations [176,199] with experimental data is needed.

## 5. Progress in CdNg Spectroscopy of the $E^{3}\Sigma_{1}^{+}({5s6s\;}^{3}S_{1})$ Rydberg State—Recently Performed OODR Experiments

Since 2015, in our lab, we have been performing a series of experiments to characterize the $E^{3}\Sigma_{1}^{+}({5s6s\;}^{3}S_{1})$ Rydberg state in CdNe, CdAr, and CdKr molecules. The experiments brought very interesting results and conclusions that moved forward ways to characterize Rydberg states and/or employ subtleties of the method to extract interesting spectroscopic characteristics that had been impossible to be deduced earlier. The results of these experiments were frequently confronted with those from available outcomes of ab initio calculations.

In this review, we present progress that has been made to the present [10,12,13,14,15,19,202]. The presentation is supplemented with extended analyses and discussions. We also present other ab initio and experimental results representing original evidence and analyses that constitute the most interesting advances in the $E^{3}\Sigma_{1}^{+}$ Rydberg state theoretical and experimental characterization in CdNg molecules. The progress should be compared with previous studies of others that are reviewed in Section 3.2 and Section 3.3, and in Section 5 below.

### 5.1. Special Approach for Rotational Characterization—Direct Bond Length Determination of the $E^{3}\Sigma_{1}^{+}({5s6s\;}^{3}S_{1})$ State in CdNe

In the case of heavy molecules, rotational spectroscopy imposes higher demand on the spectral bandwidth of the laser that is employed to resolve the relatively dense rotational energy structure. In OODR experiments, the demand is imposed on two lasers tuned to both optical transitions.

Until 2022, the spectroscopical characterization of the $E^{3}\Sigma_{1}^{+}({5s6s\;}^{3}S_{1})$ Rydberg state potential of CdNe molecule was performed only once [11]. It did not involve the rotational resolution approach. Very recently, using the OODR process, Urbańczyk et al. [10] demonstrated a selective $J^{\prime }$—excitation in the energy structure of the $E^{3}\Sigma_{1}^{+}\;$state for the first time—a smart approach that allowed them to perform rotational characterization with a laser possessing a limited spectral bandwidth. The OODR experiment employed the $E^{3}\Sigma_{1}^{+},\upsilon_{E}^{\prime },J^{\prime }⟵A^{3}\Pi_{0^{+}},\upsilon_{A}^{\prime \prime }=(0,\;1),J_{R}^{\prime \prime }⟵X^{1}\Sigma_{0^{+}},\upsilon_{X}=0,J$ path of the excitation.

**Figure 7:**
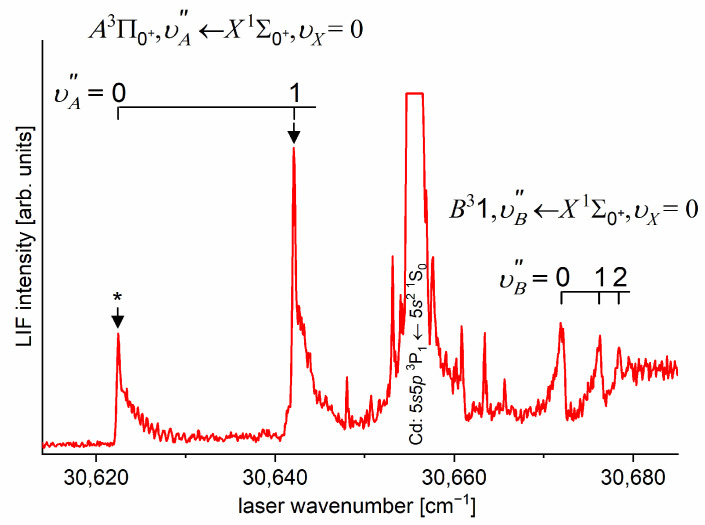
LIF excitation spectrum recorded using the $A^{3}\Pi_{0^{+}},\upsilon_{A}^{\prime \prime }⟵X^{1}\Sigma_{1}^{+},\upsilon_{X}=0$ and $B^{3}1,\upsilon_{B}^{\prime \prime }⟵X^{1}\Sigma_{1}^{+},\upsilon_{X}=0$ transitions in CdNe, as reported in Ref. [203], the former being first step of the excitation in the OODR process $E^{3}\Sigma_{1}^{+},\upsilon^{\prime }⟵A^{3}\Pi_{0^{+}},\upsilon_{A}^{\prime \prime }⟵X^{1}\Sigma_{1}^{+},\upsilon_{X}=0,$ which allowed for investigating the $E^{3}\Sigma_{1}^{+}$-state potential and, partly, potential barrier. The arrow shows the $\upsilon_{A}^{\prime \prime }=0,1$ that was used as the origin for the second transition in OODR. The asterisk depicts the vibrational band recorded in higher resolution and shown in Figure 8.

**Figure 8:**
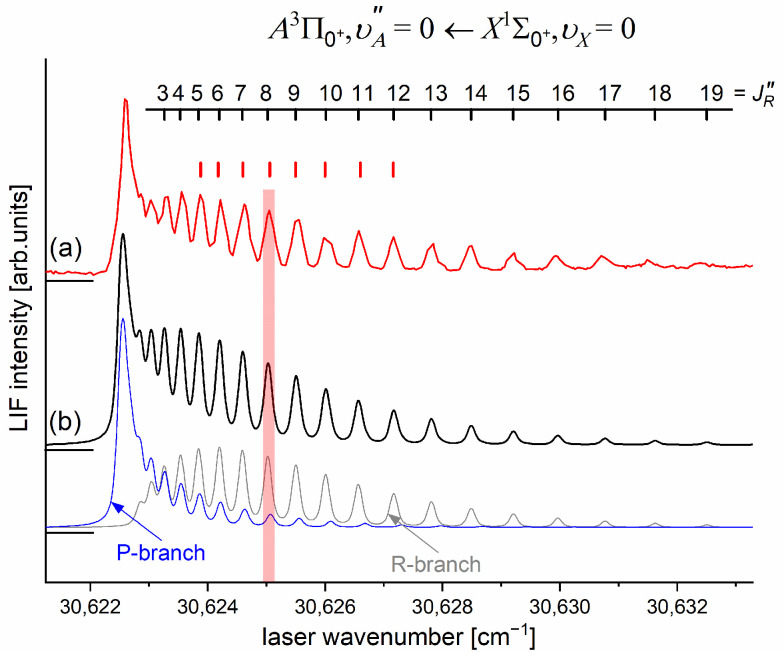
(**a**) LIF excitation spectrum showing the profile of the vibrational component recorded using the $A^{3}\Pi_{0^{+}},\upsilon_{A}^{\prime \prime }=0,J_{R}^{\prime \prime }⟵X^{1}\Sigma_{0^{+}},\upsilon_{X}=0,J$ first OODR transition in CdNe. (**b**) Simulation performed using LEVEL [205] and PGOPHER [206] programs allowed for determining the $J_{R}^{\prime \prime }$ assignment shown above the spectrum, which reveals the partly resolved structure of the R-branch (P-branch is also shown). In the simulation, $T_{rot}=5\;K$ (rotational temperature) and $\Delta_{L}=\Delta_{G}=0.1\;cm^{-1}$ were assumed (Lorentzian and Gaussian broadenings responsible for laser bandwidth and transversal divergence of molecular beam, respectively) as well as Morse representations of the $A^{3}\Pi_{0^{+}}$- and $X^{1}\Sigma_{0^{+}}$-state potentials from Ref. [203]. The positions of the $J_{R}^{\prime \prime }$ levels used as the intermediates in the OODR process (red ticks, compare with Figure 9) and a $\pm 10\;cm^{-1}$ vertical bar representing the laser bandwidth are depicted.

As the first step in the OODR process, the $A^{3}\Pi_{0^{+}},\upsilon_{A}^{\prime \prime }=0,1⟵X^{1}\Sigma_{1}^{+},\upsilon_{X}=0$ vibrational transitions were used. The corresponding low-resolution LIF excitation spectrum is shown in Figure 7 [203], whereas the profile of the $A^{3}\Pi_{0^{+}},\upsilon_{A}^{\prime \prime }=0⟵X^{1}\Sigma_{0^{+}},\upsilon_{X}=0$ transition is shown in Figure 8. It reveals a partly resolved rotational structure that, after simulation, was interpreted as pronounced transitions of R-branch $(J_{R}^{\prime \prime }=J+1)⟵J$ and condensed transitions of P-branch $\left(J_{P}^{\prime \prime }=J-1\right)⟵J,$ which constitutes the band head and partly overlaps R-branch for low $J_{R}^{\prime \prime }$ (as can be seen in Figure 8, the contribution from the P-branch outside the band head is negligibly small (which is also in accordance with the analysis by Kvaran et al. [204]), so it is not considered in the present consideration). Energy separations of R-branch transitions were large enough to choose one rotational transition to the $A^{3}\Pi_{0^{+}},\upsilon_{A}^{\prime \prime }$ state with a $0.1\;cm^{-1}$ (FWHM) spectrally broad laser. The excited rotational level in the intermediate state was applied to excite molecules using the $E^{3}\Sigma_{1}^{+},\upsilon_{E}^{\prime },J^{\prime }⟵A^{3}\Pi_{0^{+}},\upsilon_{A}^{\prime \prime }=0,J_{R}^{\prime \prime }$ second-step OODR transition.

**Figure 9:**
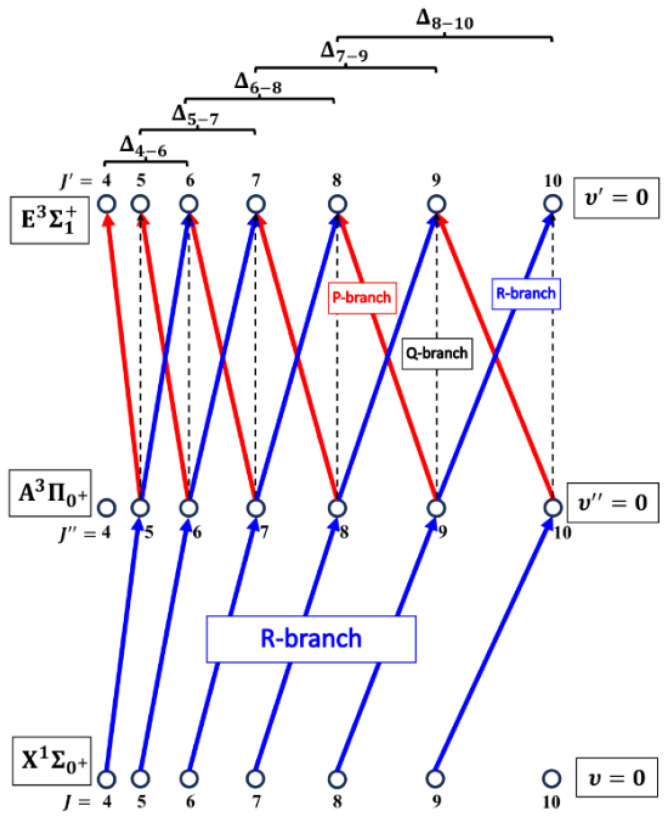
Branches of rotational transitions involved in the realization of the selective $J^{\prime }$ excitation in the OODR experiment performed in CdNe using the $E^{3}\Sigma_{1}^{+},\upsilon_{E}^{\prime }=0,J\prime ⟵A^{3}\Pi_{0^{+}},\upsilon_{A}^{\prime \prime }=0,J_{R}^{\prime \prime }⟵X^{1}\Sigma_{0^{+}},\upsilon_{X}=0,J$ transition paths. Details are provided in the text.

The rotational transitions that were involved in the OODR process are schematically shown in Figure 9. After each R-branch transition in the $A^{3}\Pi_{0^{+}}⟵X^{1}\Sigma_{0^{+}}$ first excitation, three P-, Q-, and R-branch $(J^{\prime }=J_{R}^{\prime \prime }-1)⟵J_{R}^{\prime \prime }$, $(J^{\prime }=J_{R}^{\prime \prime })⟵J_{R}^{\prime \prime }$ and $(J^{\prime }=J_{R}^{\prime \prime }+1)⟵J_{R}^{\prime \prime }$ transitions, respectively, were possible in the $E^{3}\Sigma_{1}^{+}⟵A^{3}\Pi_{0^{+}}$ second excitation.

**Figure 10:**
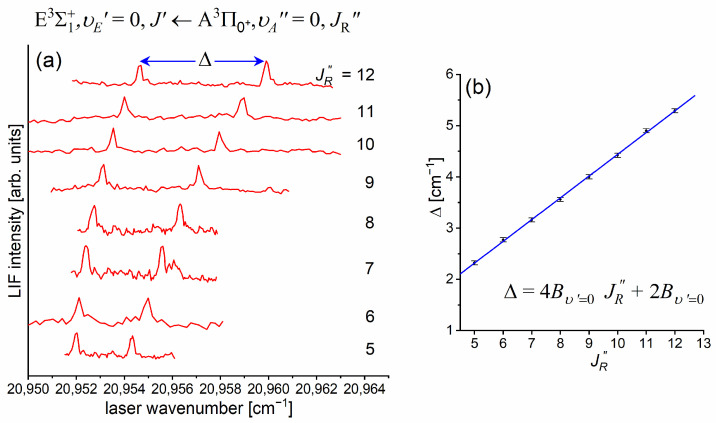
(**a**) LIF excitation spectra recorded using the $E^{3}\Sigma_{1}^{+},\upsilon_{E}^{\prime }=0,J\prime ⟵A^{3}\Pi_{0^{+}},\upsilon_{A}^{\prime \prime }=0,J_{R}^{\prime \prime }$ second OODR transition in CdNe, for $J_{R}^{\prime \prime }=5,\ldots ,12$ selected in the $A^{3}\Pi_{0^{+}},\upsilon_{A}^{\prime \prime }=0,J_{R}^{\prime \prime }⟵X^{1}\Sigma_{0^{+}},\upsilon_{X}=0,J$ first OODR transition (see also Figure 9). (**b**) Separations $\Delta (J_{R}^{\prime \prime })$ between energies of rotational transition recorded for the $P(J_{R}^{\prime \prime }-1)$ and $R(J_{R}^{\prime \prime }+1)$ branches. Linear regression allowed for determining the $B_{\upsilon^{\prime }=0}$ rotational constant.

Figure 10a presents the result of the experiment. LIF excitation spectra were recorded using the $E^{3}\Sigma_{1}^{+},\upsilon_{E}^{\prime }=0,J^{\prime }⟵A^{3}\Pi_{0^{+}},\upsilon_{A}^{\prime \prime }=0,J_{R}^{\prime \prime }$ second OODR transition after $J_{R}^{\prime \prime }=5,\ldots ,12$ were selected in the $A^{3}\Pi_{0^{+}},\upsilon_{A}^{\prime \prime }=0,J_{R}^{\prime \prime }⟵X^{1}\Sigma_{0^{+}},\upsilon_{X}=0,J$ first OODR transition, as shown in Figure 8. The $\Delta =Energy(J_{P}^{\prime })-Energy(J_{R}^{\prime })$ increase linearly with $J_{R}^{\prime \prime }$—see Figure 10b—the observed $\Delta \left(J_{R}^{\prime \prime }\right)$ dependency allowed for directly determining the $B_{\upsilon^{\prime }=0}$ rotational constant. A similar experiment was performed for the $E^{3}\Sigma_{1}^{+},\upsilon_{E}^{\prime }=1,J^{\prime }⟵A^{3}\Pi_{0^{+}},\upsilon_{A}^{\prime \prime }=0,J_{R}^{\prime \prime }$ second OODR excitation, resulting in the determination of $B_{\upsilon^{\prime }=1}$. The results are collected in Table 1.

Several essential remarks have to be stated here. Firstly, in the experiment, the isotopic structure was not resolved, as vibrational quantum numbers $\upsilon_{E}^{\prime }$ and $\upsilon_{A}^{\prime \prime }$ involved in the transitions are small. Secondly, the $E^{3}\Sigma_{1}^{+}⟵A^{3}\Pi_{0^{+}}$ transition occurred for $\Omega^{\prime }=1⟵\Omega^{\prime \prime }=0$, so according to the selection rules, the spectra should contain Q-branch as well. According to Okunishi and coworkers, however, who investigated the analogous transition in HgNe [21], the intensity of the Q-branch band-head can be significantly lower with respect to that of the P-branch.

The rotational constant $B_{e}^{\prime }$ at the equilibrium internuclear distance $R_{e}^{\prime }$ is related to $B_{\upsilon^{\prime }}$ according to the relation $B_{\upsilon^{\prime }}=B_{e}^{\prime }-\alpha_{e}^{\prime }(\upsilon^{\prime }+1/2)$, where $\alpha_{e}^{\prime }$ is a constant. Having experimentally determined $B_{\upsilon^{\prime }=0}$ and $B_{\upsilon^{\prime }=1},$ it is straightforward to calculate $B_{e}^{\prime }$ without knowing $\alpha_{e}^{\prime }$ and then, using relationship $R_{e}^{\prime }=\sqrt{h/8\pi^{2}c\mu_{CdNe}B_{e}^{\prime }}$, where h, c. and $\mu_{CdNe}$ are the Planck constant, speed of light, and reduced mass of CdNe, directly derive $R_{e}^{\prime }$ (see Table 1).

**Figure 11:**
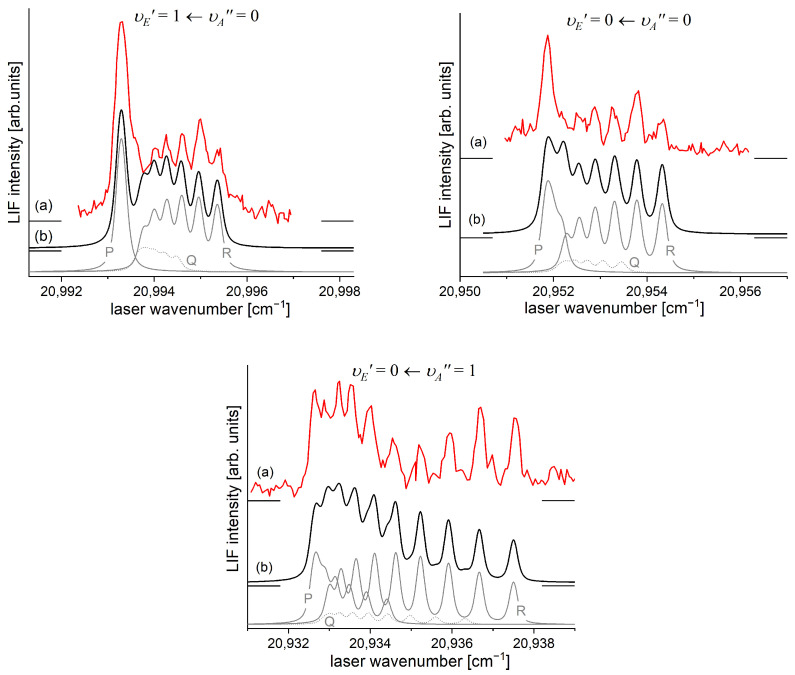
(**a**) Partly rotationally resolved profiles of vibrational bands recorded in the LIF excitation spectra of the $E^{3}\Sigma_{1}^{+},\upsilon_{E}^{\prime }⟵A^{3}\Pi_{0^{+}},\upsilon_{A}^{\prime \prime }$ second-step OODR transitions in CdNe. (**b**) Simulations performed using the LEVEL [205] and PGOPHER [206] programs: P-, Q-, and R-branches are shown. The intensity of Q-branch is damped, as concluded in Ref. [10]. All simulations are performed assuming $T_{rot}=5\;K$, $\Delta_{L}=\Delta_{G}=0.15\;cm^{-1}$, $J_{max}=6–9$ depending on the transition and the isotopic shift between CdNe isotopologues (abundances > 3%) included in the simulation as approximately one order of magnitude smaller than their rotational structure.

It is interesting to examine partly rotationally resolved profiles of vibrational bands recorded in the LIF excitation spectra of the $E^{3}\Sigma_{1}^{+},\upsilon_{E}^{\prime }=0,1⟵A^{3}\Pi_{0^{+}},\upsilon_{A}^{\prime \prime }=0,1$ second-step OODR transitions when the second excitation is not performed, as in Figure 8, with the selection of particular $J_{R}^{\prime \prime }$, but with first-step laser frequency set at the band-head where components of the P-branch are very dense. Figure 11 presents profiles of the $\upsilon_{E}^{\prime }=0⟵\upsilon_{A}^{\prime \prime }=0$, $\upsilon_{E}^{\prime }=1⟵\upsilon_{A}^{\prime \prime }=0$, and $\upsilon_{E}^{\prime }=0⟵\upsilon_{A}^{\prime \prime }=1$ transitions and their simulations [205,206], in which rotational constants derived in the above-described analysis were used. The alee simulations show satisfactory agreement with the experimental profiles, which confirms the correctness of the adopted experimental procedure and interpretation of the obtained results.

**Figure 12:**
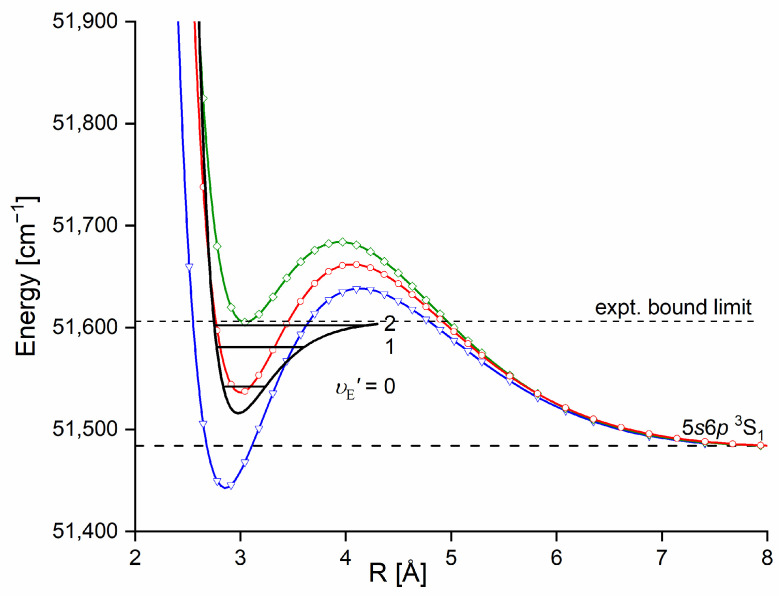
The $E^{3}\Sigma_{1}^{+}({5s6s\;}^{3}S_{1})$ Rydberg state potential well of CdNe determined experimentally [10,11] (black solid line) represented with a Morse function compared with the results of ab initio calculations from Czuchaj and Stoll [6] (blue: empty triangles and line), Czuchaj et al. [7] (green: empty squares and line), and Krośnicki and collaborators [39,40] (red: empty circles and line). Positions of $\upsilon^{\prime }$ levels supported by the potential well are depicted: $E\left(\upsilon^{\prime }=0\right)=51,542\;{cm}^{-1}$, $E\left(\upsilon^{\prime }=1\right)=51,581\;{cm}^{-1},$ $E\left(\upsilon^{\prime }=2\right)=51,602.5\;{cm}^{-1}$ as observed in the experiment. The energy limit beyond which no bound⟵bound transitions were observed is depicted.

The $E^{3}\Sigma_{1}^{+}\;$ Rydberg state potential well in CdNe accommodates three vibrational levels $\left(\upsilon_{E}^{\prime }=0,\;1,\;2\right)$, as concluded by experiment and its Morse representation; two of them were rotationally investigated, providing the position of the potential well in *R*s [10] (see Figure 12). The height of the barrier estimated from experimentally acquired data [11] should not exceed 132 ${cm}^{-1}$ above the Cd $\left({5s6s\;}^{3}S_{1}\right)$ asymptote. Additionally, having already determined the $D_{e}^{\prime }$ well depth [11], the obtained $E^{3}\Sigma_{1}^{+}$-state PEC could be confronted with results of ab initio calculations [6,7,39,40]. The comparison in Figure 12 shows that as far as the $D_{e}^{\prime }$ (from the bottom of the well to the top of the potential barrier) and the $R_{e}^{\prime }$ bond length of the $E^{3}\Sigma_{1}^{+}\;$-state potential are concerned, the closest to the experimental values ($D_{e}^{\prime \;expt}=91.0\;{cm}^{-1}$, $R_{e}^{\prime \;expt}=2.98\;Å$) (the experimental well depth $D_{e}^{\prime \;expt}$ is defined, as shown in Figure 12, from the bottom of the well to the energy beyond no bound⟵bound transitions were observed [11]) are those from the ab initio result of Czuchaj et al. [7] ($D_{e}^{\prime \;ab-initio}=79.0\;{cm}^{-1}$, $R_{e}^{\prime \;ab-initio}=3.05\;Å$). However, the ab initio result of Krośnicki and collaborators [39,40] in the closest way reproduces the position of the experimentally determined potential above the Cd asymptote. A general conclusion may state that for this relatively light CdNe molecule, an experiment-to-ab initio result comparison is moderately satisfactory and the Morse potential derived from the experiment and detection of bound⟵bound trandistions does not depart from available ab initio-calculated PECs.

### 5.2. Advances in the $E^{3}\Sigma_{1}^{+}({5s6s\;}^{3}S_{1})$-State Characterization in CdAr

#### 5.2.1. Improved Determination of the $E^{3}\Sigma_{1}^{+}$-State Inner Well Potential

By 2018, the inner well of the $E^{3}\Sigma_{1}^{+}({5s6s\;}^{3}S_{1})$ Rydberg state potential ($E^{3}\Sigma_{1\;in}^{+}$) of the CdAr molecule (see Figure 4) was investigated as a whole twice, once in 1992 [105] and once in 2003 [17]. In both studies, pronounced $\upsilon_{E_{in}}^{\prime }=0–19⟵\upsilon_{A}^{\prime \prime }=5$ vibrational progression was recorded using the $E^{3}\Sigma_{1\;in}^{+}⟵A^{3}\Pi_{0^{+}}\left(5{s5p\;}^{3}P_{1}\right)$ second OODR transition. The experiments allowed for characterizing the inner well potential and representing it with a Morse function stating at the same time that the Morse representation is not adequately close to the dissociation limit [105] or while approaching the potential barrier [17] (note: in Ref. [105], the potential barrier and outer well were not identified from experimental data).

Also, profiles of several $\upsilon_{E_{in}}^{\prime }{⟵\upsilon }^{\prime \prime }$ vibrational bands of the $e^{3}\Sigma_{1\;in}^{+}⟵b^{3}\Pi_{2}\left(5{s5p\;}^{3}P_{2}\right)$ [18] and $E^{3}\Sigma_{1\;in}^{+}⟵A^{3}\Pi_{0^{+}}$ [16] transitions were recorded in two experiments performed by employing the LV-OR and OODR methods, respectively. Bennet and Breckenridge reported high resolution spectra of $\upsilon_{e_{in}}^{\prime }=6–10{⟵\upsilon }_{b}^{\prime \prime }=0$ bands [18] as well as $B_{e}^{\prime }$ and respective $B_{\upsilon^{\prime }}$ rotational constants. Urbańczyk et al. [16] recorded partly isotopically resolved $\upsilon_{E_{in}}^{\prime }=13,14,16⟵\upsilon_{A}^{\prime \prime }=5$ bands, which allowed for, limited, however, rotational characterization of the $E^{3}\Sigma_{1}^{+}$-state inner potential well. Both characterizations were consistent with each other.

**Figure 13:**
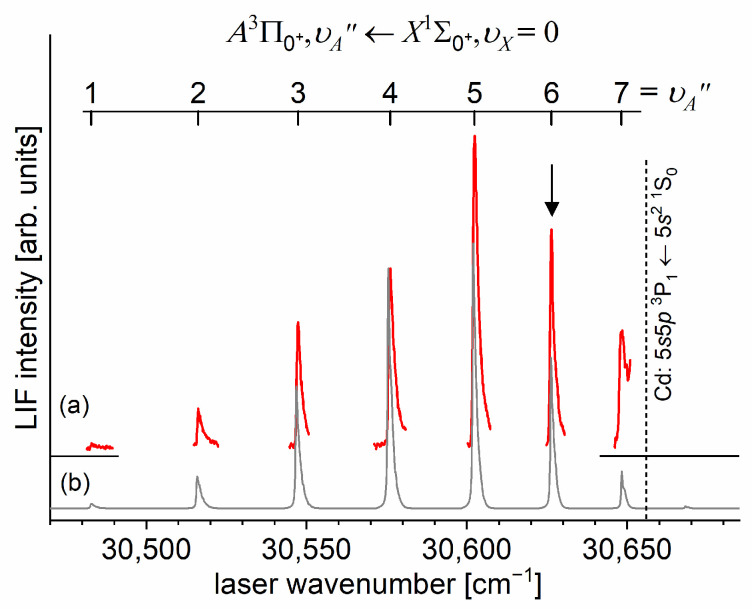
(**a**) LIF excitation spectrum recorded using the $A^{3}\Pi_{0^{+}},\upsilon_{A}^{\prime \prime }⟵X^{1}\Sigma_{1}^{+},\upsilon =0$ transition in CdAr, being first step of excitation in the OODR process $E^{3}\Sigma_{1\;in}^{+},\upsilon_{E_{in}}^{\prime }⟵A^{3}\Pi_{0^{+}},\upsilon_{A}^{\prime \prime }⟵X^{1}\Sigma_{1}^{+},\upsilon =0$, which allowed for investigating the $E^{3}\Sigma_{1in}^{+}$-state inner well and, partly, potential barrier. (**b**) Simulation performed using the LEVEL [205] and PGOPHER [206] programs and data derived from the analysis of the experimental spectrum [13]. The arrow shows the $\upsilon_{A}^{\prime \prime }=6$ that was used as the origin for the second transition in OODR. Also, the $\upsilon_{A}^{\prime \prime }=6⟵\upsilon =0$ band recorded with higher resolution was used, among others, in the isotopologue selection experiment (see Section 5.4). The position of the atomic transition in Cd is depicted.

**Figure 14:**
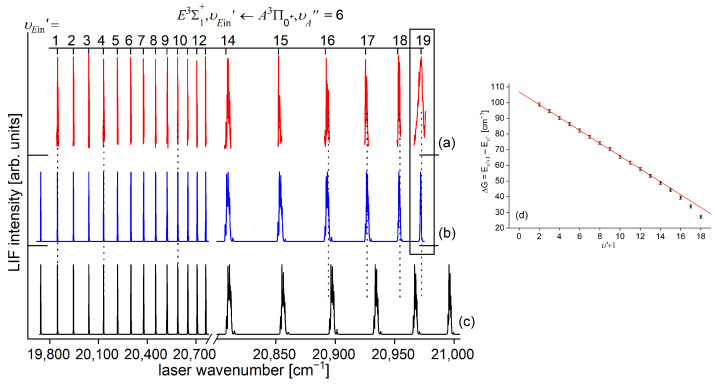
(**a**) LIF excitation spectrum of the $\upsilon_{E_{in}}^{\prime }=0–19⟵\upsilon_{A}^{\prime \prime }=6$ progression recorded using $E^{3}\Sigma_{1\;in}^{+}⟵A^{3}\Pi_{0^{+}}$ in CdAr. (**b**) Simulations performed using a representation of the $E^{3}\Sigma_{1}^{+}$-state inner well (**b**) obtained from the IPA method and (**c**) using a Morse function. As the figure compares only the positions of the vibrational components, their intensities in (**a**–**c**) were normalized. To make the comparison easier, several last $\upsilon_{E_{in}}^{\prime }⟵\upsilon_{A}^{\prime \prime }=6$ components are shown on different horizontal scales. Details of the $\upsilon_{E_{in}}^{\prime }=19⟵\upsilon_{A}^{\prime \prime }=6$ component, shown in the rectangular frame. (**d**) B-S plot for the $\upsilon_{E_{in}}^{\prime }=0–19⟵\upsilon_{A}^{\prime \prime }=6$ progression presenting a distinct nonlinearity for approx. $\upsilon_{E_{in}}^{\prime }>12$.

In a very recent experiment by Urbańczyk et al. [13], the $E^{3}\Sigma_{1}^{+}$-state inner well was reinvestigated and $\upsilon_{E_{in}}^{\prime }=0–19⟵\upsilon_{A}^{\prime \prime }=6$ progression was recorded with higher accuracy and with a spectrally narrower laser than that in Ref. [17]. Figure 13 and Figure 14 present LIF excitation spectra recorded using transitions corresponding to the first ($A^{3}\Pi_{0^{+}},\upsilon_{A}^{\prime \prime }⟵X^{1}\Sigma_{1}^{+},\upsilon_{X}=0$) and second step ($E^{3}\Sigma_{1\;in}^{+},\upsilon_{E_{in}}^{\prime }⟵A^{3}\Pi_{0^{+}},\upsilon_{A}^{\prime \prime }=6$) of the OODR process, respectively.

Here, it is crucial to notice that mutual positions of the $E^{3}\Sigma_{1}^{+}$- and $A^{3}\Pi_{0^{+}}$-state potentials allow (providing proper choice of $\upsilon_{A}^{\prime \prime }$) for recording a whole vibrational progression termination at all $\upsilon_{E_{in}}^{\prime }$ supported by the $E^{3}\Sigma_{1}^{+}$-state potential inner well. This gives an opportunity to characterize the inner well in the best possible way. The improvement in Ref. [13] was achieved by thorough analysis and simulation of the spectrum, providing a more consistent characterization of the inner well based on the inverted perturbation approach (IPA) [207]. Moreover, $\upsilon_{E_{in}}^{\prime }=2,5,11,17{⟵\upsilon }_{A}^{\prime \prime }=6$ vibrational bands were recorded with higher resolution, which provided their more reliable rotational characterization (see analysis below). To complete the inner well characterization, free⟵bound ($\upsilon_{A}^{\prime \prime }=6)$ transitions were included in the analysis (see analysis below).

The IPA method starts from the chosen ab initio or analytical potential expressed in E(R) pointwise form and optimizes E of specific points in order to obtain the best agreement between $E\left(\upsilon^{\prime }\right)$ vibrational and $E\left(J^{\prime }\right)$ rotational energy levels, determined by solving the Schrödinger equation and energies recorded in experimental spectra. Importantly, optimization of the pointwise potential, which possesses more degrees of freedom than, e.g., analytical potential, allows for a better agreement between the simulated and experimental energies and provides a better, more reliable results for PEC representation.

The IPA method employed for the $\upsilon_{E_{in}}^{\prime }=0–19⟵\upsilon_{A}^{\prime \prime }=6$ progression concluded with a better simulation of the LIF excitation spectrum with the $E^{3}\Sigma_{1}^{+}$-state inner well represented by the pointwise potential. Figure 14 shows a comparison of the $\upsilon_{E_{in}}^{\prime }=0–19⟵\upsilon_{A}^{\prime \prime }=6$ progression recorded in the experiment with two simulations as follows: that obtained from the IPA method and that with the $E^{3}\Sigma_{1}^{+}$-state inner well represented by a Morse function. What is evident from the comparison is an inadequacy of Morse-function representation for approximately $\upsilon_{E_{in}}^{\prime }>12$ (which is a generally acknowledged conclusion a Morse function most adequately representing molecular potential in the vicinity of the bottom of the potential well), a problem that is eliminated by the IPA result. The conclusion is also demonstrated in the respective Birge–Sponer (B-S) plot shown in the inset. The conclusion is in line with the Rydberg character of the $E^{3}\Sigma_{1}^{+}$-state and a conclusion reached by Krośnicki et al. [4], showing the presence of a maximum in the Rydberg-electron density distribution in the region of the outer wall (i.e., which is closer to the barrier) in the $E^{3}\Sigma_{1}^{+}$-state inner well.

**Figure 15:**
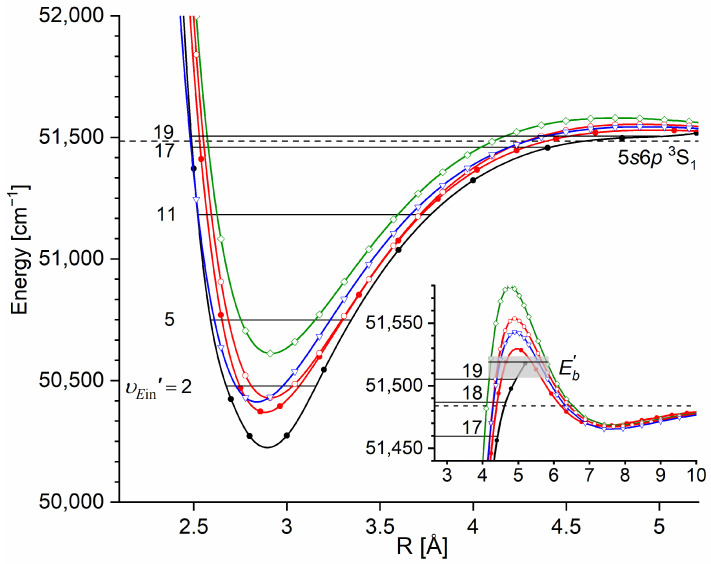
Comparison of the $E^{3}\Sigma_{1}^{+}\left({5s6s\;}^{3}S_{1}\right)$-state inner well IPA representation in CdAr (black: full circles and line) with the results of the most recent ab initio calculations by Krośnicki et al. [4] (red: full circles and line) and ab initio calculations by Czuchaj and Stoll [6] (blue: empty triangles and line), Czuchaj et al. [7] (green: empty squares and line), and Krośnicki and collaborators [39,40] (red: empty circles and line). The inset shows vicinity of the potential barrier that separates the inner and the outer wells. The position of three $\upsilon_{E_{in}}^{\prime }$ levels closest to the dissociation energy is shown. The height of the potential barrier $E_{b}^{\prime }$ is as estimated in Ref. [13] (grey rectangle) and as determined in Ref. [12] (horizontal black line) (also see Table 1).

A comparison of the IPA result with available ab initio inner well representations is shown in Figure 15. It is evident that the $E^{3}\Sigma_{1}^{+}$-state IPA representation of the inner well is deeper than the depths obtained in all ab initio results. Additionally, three ab initio-calculated potentials, i.e., those by Czuchaj and Stoll [6] and Krośnicki and collaborators [39,40] as well as that, very recently, by Krośnicki et al. [4], are very close to each other as far as the depth of the potential well is concerned. As can be seen in Figure 14 (and Figure 15), the IPA method, by definition, provided a very reliable representation of the $E^{3}\Sigma_{1}^{+}$-state inner well potential. But one has to deal here with more complex, i.e., double-well, potential; therefore, the inner well potential representation of the $E^{3}\Sigma_{1}^{+}\;$ state should join smoothly with a reliable representation of the potential barrier and representation of the potential outer well that extends for larger *R*s.

**Figure 16:**
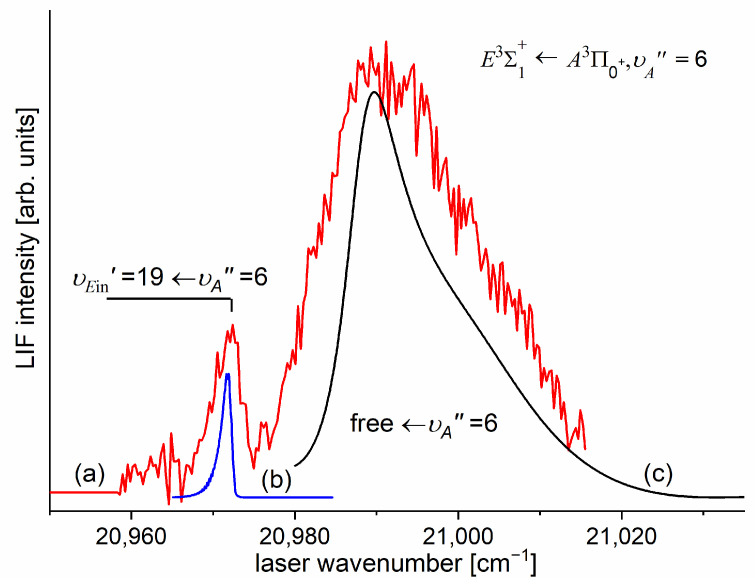
(**a**) LIF excitation spectrum recorded using the $E^{3}\Sigma_{1}^{+}⟵A^{3}\Pi_{0^{+}}$ transition in CdAr [13], showing the last $\upsilon_{E_{in}}^{\prime }=19⟵\upsilon_{A}^{\prime \prime }=6$ quasi-bound⟵bound transition and profile of free⟵bound transitions starting from $\upsilon_{A}^{\prime \prime }=6$. (**b**) and (**c**) Simulations of the bound⟵bound and free⟵bound transitions performed using the PGOPHER [206] and BCONT [209] programs, respectively.

In order to investigate the two crucial joining points, it was necessary to reach the potential barrier in the excitation from both sides, i.e., to record $\upsilon_{E}^{\prime }{⟵\upsilon }_{A,B}^{\prime \prime }$ progressions to the highest $\upsilon_{E}^{\prime }$ in both the $E^{3}\Sigma_{1}^{+}⟵A^{3}\Pi_{0^{+}}$ and $E^{3}\Sigma_{1}^{+}⟵B^{3}1$ transitions (refer to Figure 4). As far as the former is concerned, Figure 16 shows the $\upsilon_{E_{in}}^{\prime }=19⟵\upsilon_{A}^{\prime \prime }=6$ last quasi-bound⟵bound transition lying (along with that to $\upsilon_{E_{in}}^{\prime }=18$) above the dissociation energy correlating with the $\left({5s6s\;}^{3}S_{1}\right)$ Cd asymptote (see Figure 15) ($\upsilon_{E_{in}}^{\prime }=19$ and $\upsilon_{E_{in}}^{\prime }=18$ are regarded as quasi-bound resonant vibrational levels lying above the dissociation energy and supported by the presence of the potential barrier). Also, Figure 16 shows a wide profile of free⟵bound transitions starting from $\upsilon_{A}^{\prime \prime }=6$ and terminating at the repulsive inner wall of the potential. Simulation of both parts of the spectrum assuming representation of the $E^{3}\Sigma_{1}^{+}\;$-state inner well by the IPA result confirmed once again its advantage over that of a Morse function. However, simulation of the free⟵bound transitions shown in Figure 16c had to be shifted by 5 ${cm}^{-1}$ towards larger wavenumbers to reproduce the experimental spectrum—this may suggest that the steepness of the repulsive part of the inner well potential is somewhat smaller than assumed in the simulation. The height of the barrier was estimated to be in the 21.1–39.4 ${cm}^{-1}$ energy interval above the $\left({5s6s\;}^{3}S_{1}\right)$ Cd asymptote (see inset in Figure 15).

**Figure 17:**
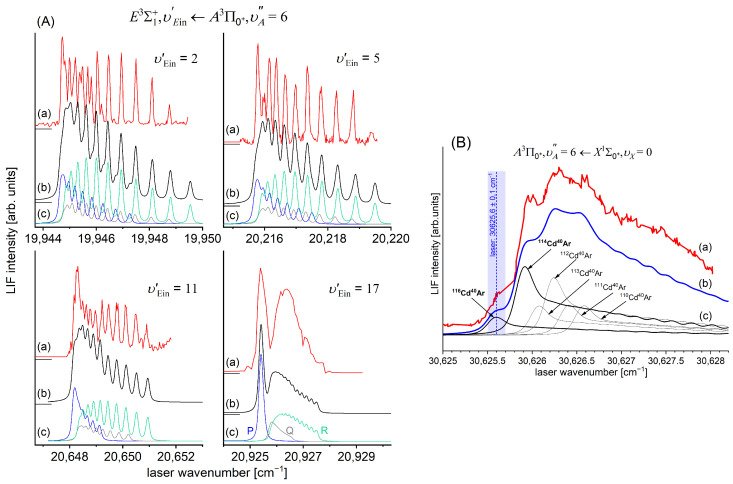
(**A**) Partly rotationally resolved profiles of vibrational bands recorded in the LIF excitation spectrum of the $E^{3}\Sigma_{1\;in}^{+},\upsilon_{E_{in}}^{\prime }⟵A^{3}\Pi_{0^{+}},\upsilon_{A}^{\prime \prime }=6$ second-step OODR transition in ^116^${Cd}^{40}Ar$ (with a small admixture of ^114^${Cd}^{40}Ar$). (**a**) Experimental spectra. (**b**) Simulations performed using the LEVEL [205] and PGOPHER [206] programs assuming $T_{rot}=2.5\;K\left(\upsilon_{E_{in}}^{\prime }=2\;and\;5\right),T_{rot}=3.5\;K\left(\upsilon_{E_{in}}^{\prime }=11\right),$ $T_{rot}=$5 K ($\upsilon_{E_{in}}^{\prime }=17$), and $\Delta_{L}=\Delta_{G}=0.1\;cm^{-1}$. (**c**) Intensities of rotational P-, Q- and R-branches contributing to (**b**), depicted with a colour code in part for $\upsilon_{E_{in}}^{\prime }=17$. The intensity of the Q-branch is damped, as concluded in Ref. [10]. (**B**) The profile of the $A^{3}\Pi_{0^{+}},\upsilon_{A}^{\prime \prime }=6⟵X^{1}\Sigma_{1}^{+},\upsilon =0$ first-step OODR transition showing the possibility of selective excitation of ^116^${Cd}^{40}Ar$ with a small admixture of ^114^${Cd}^{40}Ar$ isotopologue (see also Section 5.4). (**a**) Experimental LIF excitation spectrum, (**b**) total simulation of the profile, and (**c**) contributions to the total simulated profile corresponding to different CdAr isotopologues. Vertical blue dashed line and wide bar depict a laser wavenumber of the first-step OODR transition and the laser bandwidth, respectively.

As far as the position of the inner well ($R_{e\;in}^{\prime })$ is concerned, it was corroborated by detecting and simulating a series of partly rotationally resolved profiles of vibrational bands of the $E^{3}\Sigma_{1in}^{+},\upsilon_{E_{in}}^{\prime }⟵A^{3}\Pi_{0^{+}},\upsilon_{A}^{\prime \prime }$ transition in ^116^${Cd}^{40}Ar$ with a small admixture of ^114^${Cd}^{40}Ar$. A selective isotopologue excitation exploiting the $A^{3}\Pi_{0^{+}},\upsilon_{A}^{\prime \prime }=6⟵X^{1}\Sigma_{1}^{+},\upsilon =0$ first-step OODR transition was possible, as shown in Figure 17B and discussed in more detail in Section 5.4 of this review. Figure 17A presents four recorded profiles corresponding to $\upsilon_{E_{in}}^{\prime }=2,\;5,\;11,\;17⟵\upsilon_{A}^{\prime \prime }=6$ vibrational transitions. Simulation of the profiles allowed for determining respective $B_{\upsilon_{E_{in}}^{\prime }}$ rotational constants and corroborating the $R_{e\;in}^{\prime }=2.850\pm 0.005\;Å$ as that verified by also employing the IPA method. As already stated, rotationally resolved vibrational profiles were recorded by Bennet and Breckenridge [18], but they belonged to the $e^{3}\Sigma^{+},\;\upsilon_{e_{in}}^{\prime }⟵b^{3}\Pi_{2},\;\upsilon_{b}^{\prime \prime }$ transition after highly nonselective LV process and assuming $\upsilon_{b}^{\prime \prime }=0$.

In Figure 15, it is obvious that, similar to the $E^{3}\Sigma_{1}^{+}$ Rydberg state in CdNe, in CdAr, agreement between the experimental and ab initio-calculated potentials is not entirely satisfactory for either the inner well or the potential barrier. It was necessary to and characterize the complex $E^{3}\Sigma_{1}^{+}$-state double-well potential for all three components including both the inner and outer wells and the barrier. This is presented in Section 5.2.3.

#### 5.2.2. Agreement Plot, Agreement Parameter, and a New Method for the Outer Well $R_{e\;out}^{\prime }$ Bond Length Adjustment

Experimental evidence of the existence of the $E^{3}\Sigma_{1}^{+}({5s6s\;}^{3}S_{1})$ Rydberg state outer well ($E^{3}\Sigma_{1\;out}^{+}$) in the CdAr molecule, which corroborated the findings of ab initio calculation, was not known until 2003. For the first time, Koperski and Czajkowski [17] reported the presence of a second shallow outer well that was separated from the inner well ($E^{3}\Sigma_{1\;in}^{+}$) by the potential barrier. From LIF excitation $E^{3}\Sigma_{1\;out}^{+},\upsilon_{E_{out}}^{\prime }⟵B^{3}1,\upsilon_{B}^{\prime \prime }=0,1,2$ bound←bound and free←bound spectra recorded with limited spectral resolution, they characterized the outer well and attempted to join the inner and outer wells with an arbitrarily chosen polynomial function.

**Figure 18:**
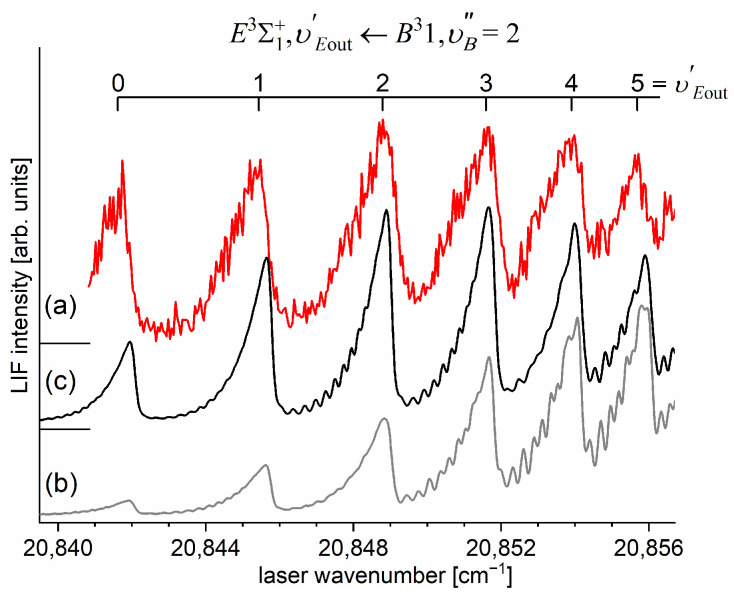
The $E^{3}\Sigma_{1\;out}^{+},\upsilon_{E_{out}}^{\prime }⟵B^{3}1,\upsilon_{B}^{\prime \prime }=2$ vibrational band of CdAr. (**a**) Experimental spectrum. Simulations performed using the PGOPHER program [206] assuming that (**b**) $R_{e\;out}^{\prime }$ = 7.63 Å [16] and (**c**) $R_{e\;out}^{\prime }$ = 6.90 Å were obtained as a result of the new method of bond length adjustment with the help of the $C\left(R_{e\;out}^{\prime }\right)$ agreement parameter [15]. In both simulations, $T_{rot}=5\;K$ and $\Delta_{L}=\Delta_{G}=0.1\;cm^{-1}$ were used. Note: according to later studies, the figure shows the wrong $\upsilon_{Eout}^{\prime }$ assignment as it lacks the low-intensity $\upsilon_{Eout}^{\prime }=0⟵\upsilon_{B}^{\prime \prime }=2$ component that was recorded during the investigation of the $E^{3}\Sigma_{1\;out}^{+},\upsilon_{E_{out}}^{\prime }⟵B^{3}1,\upsilon_{B}^{\prime \prime }$ transitions [12], causing correction of the spectroscopical characterization of the $E^{3}\Sigma_{1\;out}^{+}$ well. This is also illustrated in Figure 19 and Figure 20.

**Figure 19:**
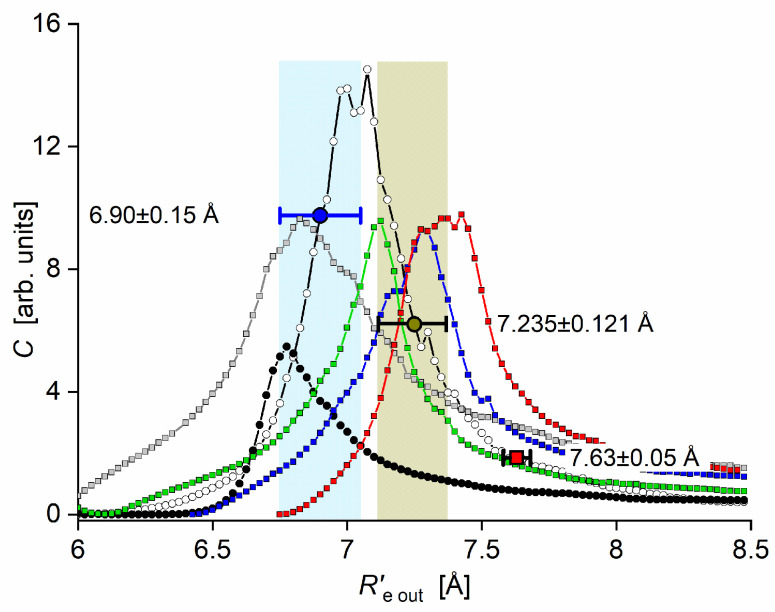
The $C\left(R_{e\;out}^{\prime }\right)$ agreement coefficient describing the agreement between $I_{expt}^{\left(i\right)}$ experimental and $I_{sim}^{\left(i\right)}(R_{e\;out}^{\prime })$ simulated intensities of the vibrational components in the LIF excitation spectrum recorded using the $E^{3}\Sigma_{1out}^{+},\upsilon_{E_{out}}^{\prime }⟵B^{3}1,\upsilon_{B}^{\prime \prime }$ transition (see Equation (2)) for $\upsilon_{B}^{\prime \prime }=1$ (black full circles and line) [15] and $\upsilon_{B}^{\prime \prime }=2$ (black empty circles and line) [15]. The plot is supplemented with the recent results of Ref. [12], which concluded in the correction of the $\upsilon_{E_{out}}^{\prime }$ assignment (see Section 5.2.3) for $\upsilon_{B}^{\prime \prime }=1$ (grey squares and line), $\upsilon_{B}^{\prime \prime }=2$ (green squares and line), $\upsilon_{B}^{\prime \prime }=3$ (blue squares and line), and $\upsilon_{B}^{\prime \prime }=4$ (red squares and line). Symbols with error bars and corresponding rectangles depict the $R_{e\;out}^{\prime }$ results of Refs. [12,15] and, for comparison, Ref. [16].

Further investigation [16] was devoted to the detection of $E^{3}\Sigma_{1\;out}^{+},\upsilon_{E_{out}}^{\prime }=0–6⟵B^{3}1,\upsilon_{B}^{\prime \prime }=1$ vibrational bands and was conducted with higher spectral resolution, which allowed for partly resolving the rotational structure. Moreover, in Ref. [17], as a result of the better spectral resolution than in Ref. [16], the $\upsilon_{E_{out}}^{\prime }$ assignment in the spectrum was changed, i.e., $\upsilon_{E_{out}}^{\prime }$ of Ref. [16] equals $\upsilon_{E_{out}}^{\prime }$ of Ref. [17] minus one. Consequently, the determination of the $E^{3}\Sigma_{1\;out}^{+}$ well depth ($D_{e\;out}^{\prime }$) and bond length ($R_{e\;out}^{\prime }$) was improved to new values (see Table 1). Later on, a new method for $R_{e\;out}^{\prime }$ adjustment was proposed along with revisitation of the $E^{3}\Sigma_{1\;out}^{+}⟵B^{3}1$ spectrum [15]. The $E^{3}\Sigma_{1\;out}^{+},\upsilon_{E_{out}}^{\prime }=0–6⟵B^{3}1,\upsilon_{B}^{\prime \prime }=2$ vibrational band was additionally recorded and, along with that originating from $\upsilon^{\prime \prime }=1$, it served as input data for the proposal based on the fact that the $R_{e\;out}^{\prime }$ = 7.63 Å [16] simulation of both spectra, i.e., originating from $\upsilon_{B}^{\prime \prime }=1$ and from $\upsilon_{B}^{\prime \prime }=2$, showed considerable disagreement with the experimental ones as far as the distribution of vibrational component intensities is concerned (see traces (a) and (b) in Figure 18). To resolve the problem, the so-called agreement parameter was proposed

(2) $$C\left(R_{e\;out}^{\prime }\right)=\frac{1}{\sum_{i}{\left[I_{expt}^{\left(i\right)}-I_{sim}^{\left(i\right)}(R_{e\;out}^{\prime })\right]}^{2}},$$

where $I_{expt}^{\left(i\right)}$ and $I_{sim}^{\left(i\right)}(R_{e\;out}^{\prime })$ are the normalized experimental and simulated intensities of the *i*th vibrational component in the LIF excitation spectrum. Such a definition of the $C\left(R_{e\;out}^{\prime }\right)$ causes it to achieve the highest values only for those $R_{e\;out}^{\prime }$ for which the intensity distribution of the simulated vibrational components are close to the intensity of the respective components in the experimental spectrum. Figure 19 shows $C\left(R_{e\;out}^{\prime }\right)$ agreement coefficient for the $E^{3}\Sigma_{1\;out}^{+},\upsilon_{E_{out}}^{\prime }⟵B^{3}1,\upsilon_{B}^{\prime \prime }$ transition originating from (a) $\upsilon_{B}^{\prime \prime }=1$ (empty circles) and (b) $\upsilon_{B}^{\prime \prime }=2$ (full circles) along with the resulting $R_{e\;out}^{\prime }=6.90\pm 0.15\;Å$ [15]. For comparison, the result of Ref. [16] is also shown. Moreover, the plot in Figure 19 is augmented with the best result so far, showing improvement in the $R_{e\;out}^{\prime }$ determination, i.e., 7.235±0.121 Å [12], resulting from the accumulation of more experimental data and correction of $\upsilon_{E_{out}}^{\prime }$ assignment (see Section 5.2.3).

**Figure 20:**
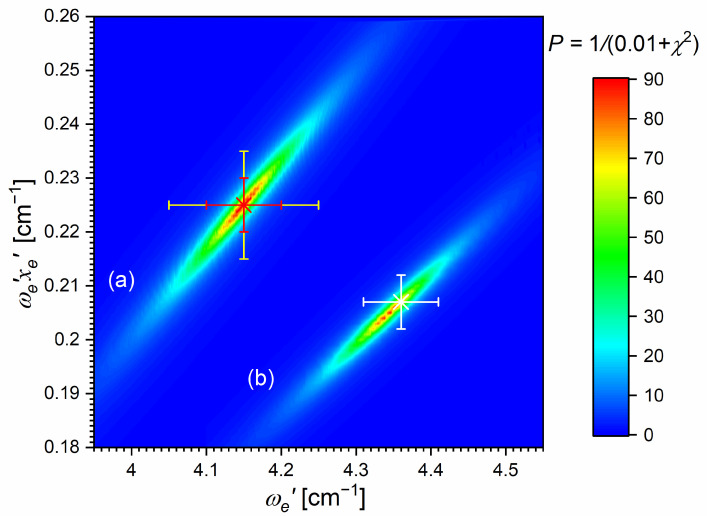
The $\left(\omega_{e}^{\prime }-\omega_{e}^{\prime }x_{e}^{\prime }\right)$ *agreement plot* drawn according to Equation (3), expressing simulation-to-experiment agreement with respect to the $E^{3}\Sigma_{1out}^{+}$ state of CdAr using LIF excitation spectra of the $E^{3}\Sigma_{1out}^{+},\upsilon_{E_{out}}^{\prime }⟵B^{3}1,\upsilon_{B}^{\prime \prime }$ transitions for (**a**) $\upsilon_{B}^{\prime \prime }=1,$ as in Ref. [15], and (**b**) $\upsilon_{B}^{\prime \prime }=3,$ as in Ref. [12], after recording an additional component in the spectrum and correction of the $\upsilon_{E_{out}}^{\prime }$ assignment (see Section 5.2.3). The plot shows the dependency between $\omega_{e}^{\prime }$ and $\omega_{e}^{\prime }x_{e}^{\prime }$ vibrational constants. Error bars: uncertainties in $\omega_{e}^{\prime }$ and $\omega_{e}^{\prime }x_{e}^{\prime },$ as in Ref. [16] (red), Ref. [15] (yellow), and, finally, Ref. [12] (white).

Apart from the new method of the agreement coefficient C, another method, called the agreement plot, was proposed in Ref. [15] as complementary to, e.g., the B-S plot with respect to the determination of the $\omega_{e}^{\prime }$ and $\omega_{e}^{\prime }x_{e}^{\prime }$ vibrational constants. It can be used provided that the upper electronic-state potential (to which excitation occurs) is represented by a Morse function. The agreement plot illustrates the fact that $\omega_{e}^{\prime }$ and $\omega_{e}^{\prime }x_{e}^{\prime }$ are not independent and there is a number of ${(\omega }_{e}^{\prime },\omega_{e}^{\prime }x_{e}^{\prime })$ pairs resulting in satisfactory simulation of the experimental spectrum (with not resolved rotational structure). Also, the agreement plot allows for determining the $\omega_{e}^{\prime }$ and $\omega_{e}^{\prime }x_{e}^{\prime }$ uncertainties in a more reliable way than when using the B-S plot. The agreement plot method relies on the calculated value of the *P* coefficient (see Figure 20) expressed by the following formula. (The P coefficient is calculated to construct the *agreement plot* as a so-called heatmap plot. Consequently, the numerical (dimensionless) value of P becomes important. While $\Delta E_{\upsilon^{\prime }}^{sim}$ and $\Delta E_{\upsilon^{\prime }}^{expt}$ are expressed in ${cm}^{-1}$, $\chi^{2}$ is also expressed in ${cm}^{-2}$. The parameters in Equation (3) were selected so that P is always in the range from 0 to 100; therefore, the denominator 0.01 was added to introduce this upper restriction. To ensure that P is dimensionless, parameter 0.01 in the denominator as well as 1 in the numerator had to be expressed in ${cm}^{-2}$ as well.)

(3) $$P=\frac{1}{0.01+\chi^{2}},$$

where $\chi^{2}=\sum_{\upsilon^{\prime }}(\Delta E_{\upsilon^{\prime }}^{expt}-\Delta E_{\upsilon^{\prime }}^{sim}{)}^{2}$, $\Delta E_{\upsilon^{\prime }}^{sim}$ is the separation between the energies of corresponding $\upsilon^{\prime }$: $\Delta E_{\upsilon^{\prime }}^{sim}=E_{\upsilon^{\prime }}^{sim}-E_{{int\;\upsilon }^{\prime }}^{sim}$, where $E_{{int\;\upsilon }^{\prime }}^{sim}$ is the energy of a selected (for example, the most intense) $E^{3}\Sigma_{1out}^{+},\upsilon_{E_{out}}^{\prime }⟵{B^{3}1,\upsilon }_{B}^{\prime \prime }=1$ transition in the experimental spectrum, and $\Delta E_{\upsilon^{\prime }}^{expt}$ is the separation between energies of vibrational components in the experimental spectrum calculated similar to $\Delta E_{\upsilon^{\prime }}^{sim}$. Next, for each $\omega_{e}^{\prime }$ and $\omega_{e}^{\prime }x_{e}^{\prime }$ combination, the $\Delta E_{\upsilon^{\prime }}^{sim}$ values were computed (LEVEL program [205]) and $\Delta E_{\upsilon^{\prime }}^{expt}$ values were determined from the experimental spectrum, leading to $P{(\omega }_{e}^{\prime },\omega_{e}^{\prime }x_{e}^{\prime })$ according to Equation (3). The result for the $E^{3}\Sigma_{1\;out}^{+},\upsilon_{E_{out}}^{\prime }⟵B^{3}1,\upsilon_{B}^{\prime \prime }=1$ transition [15] is shown in Figure 20a, whereas Figure 20b shows the result after recording an additional component in the spectrum and a change in the $\upsilon_{E_{out}}^{\prime }$ assignment (see Section 5.2.3) [12]. It should be emphasized that each of the ${(\omega }_{e}^{\prime },\omega_{e}^{\prime }x_{e}^{\prime })$ pairs for which P>30 results in satisfactory simulation-to-experiment agreement.

#### 5.2.3. Final Approach: The $E^{3}\Sigma_{1}^{+}$-State Complete Potential Determination

Thanks to the very recent investigation of LIF excitation spectra recorded using the $E^{3}\Sigma_{1\;out}^{+}⟵B^{3}1,\upsilon_{B}^{\prime \prime }$ transitions [12], the respective measurement range has been widened as compared with the previous studies [15] described in Section 5.2.2. Both, bound⟵bound and free⟵bound type of transitions in the excitation spectra were recorded and involved in the analysis; moreover, a number of $\upsilon_{B}^{\prime \prime }$ levels from which the excitation originates was increased from $\upsilon_{B}^{\prime \prime }=0–2$ to $\upsilon_{B}^{\prime \prime }=0–4$. Consequently, the study became the most complete characterization of the $E^{3}\Sigma_{1\;out}^{+}$ Rydberg state potential of CdAr performed to date, and together with earlier studies of the $E^{3}\Sigma_{1}^{+}$-state inner-well [13], constituted the most thorough description of the MeNg Rydberg state potential based on experimental data.

**Figure 21:**
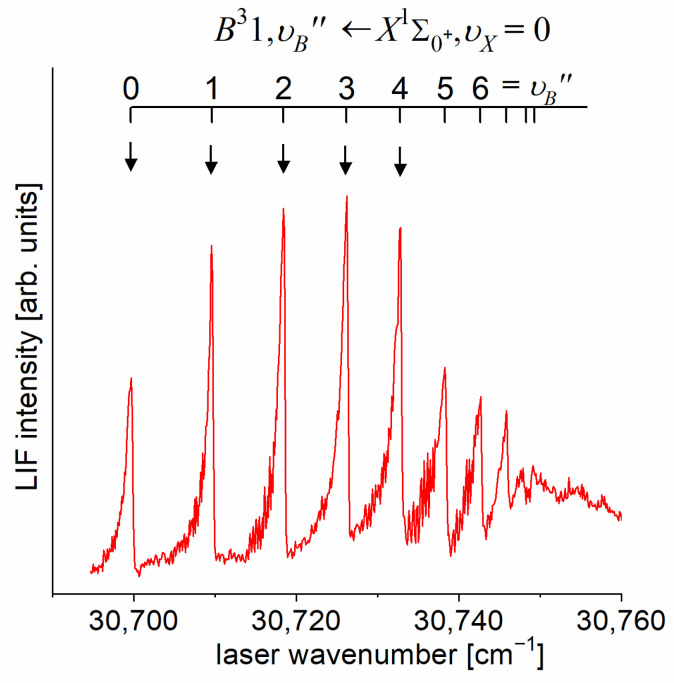
LIF excitation spectrum recorded using the $B^{3}1,\upsilon_{B}^{\prime \prime }⟵X^{1}\Sigma_{1}^{+},\upsilon_{X}=0$ transition in CdAr, as reported in Ref. [210], which is the first step of excitation in the OODR process that allowed for investigating the $E^{3}\Sigma_{1\;out}^{+}$-state outer well [12,15] and potential barrier [12] using the $E^{3}\Sigma_{1\;out}^{+},\upsilon_{E_{out}}^{\prime }⟵B^{3}1,\upsilon_{B}^{\prime \prime }$ second-step OODR transition. Arrows show $\upsilon_{B}^{\prime \prime }$ values that were used as origins in the second-step transition.

**Figure 22:**
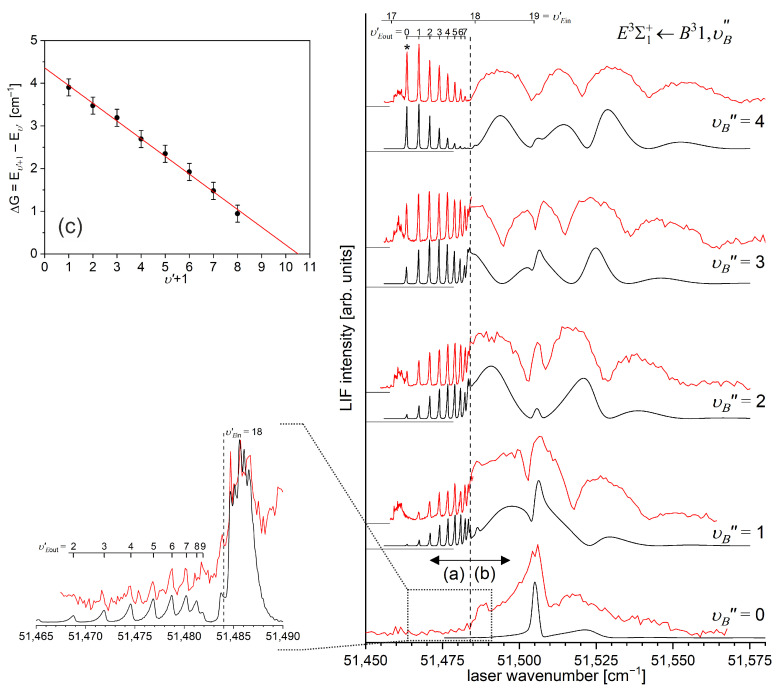
LIF excitation spectra (red lines) recorded using (**a**) the $E^{3}\Sigma_{1\;out}^{+},\upsilon_{E_{out}}^{\prime }⟵B^{3}1,\upsilon_{B}^{\prime \prime }=0–4$ bound⟵bound and (**b**) the $E^{3}\Sigma_{1}^{+}⟵B^{3}1,\upsilon_{B}^{\prime \prime }=0–4$ free⟵bound transitions in CdAr, and their respective simulations [205,206,209] (black lines). The position $\upsilon_{E_{out}}^{\prime }=0⟵\upsilon_{B}^{\prime \prime }$ component that was not previously recorded [15] is depicted (one asterisk). The position of the $E^{3}\Sigma_{1\;in}^{+},\upsilon_{E_{in}}^{\prime }=17–19⟵B^{3}1,\upsilon_{B}^{\prime \prime }$ vibrational components is shown, proving that the spectrum also contains transitions to the $E^{3}\Sigma_{1}^{+}$-state inner well. Unlike in other spectra recorded using second-step OODR transition and presented in this review, here, laser wavenumbers are given with respect to the $X^{1}\Sigma_{1}^{+}$-state asymptote. The energy corresponding to the $\left({5s6s\;}^{3}S_{1}\right)$ Cd asymptote is depicted (vertical dashed line). The inset to (**a**) shows details of the very weak ${\upsilon_{E_{out}}^{\prime }⟵\upsilon }_{B}^{\prime \prime }=0$ bound⟵bound transitions with clearly visible $\upsilon_{E_{in}}^{\prime }=18⟵\upsilon_{B}^{\prime \prime }=0$. (**c**) B-S plot for $\upsilon_{E_{out}}^{\prime }⟵\upsilon_{B}^{\prime \prime }=4$ progression and with corrected $\upsilon_{E_{out}}^{\prime }$ assignment, providing final values for the $E^{3}\Sigma_{1\;out}^{+}$ outer well vibrational constants $\omega_{e}^{\prime }=4.36\;{cm}^{-1}$ and $\omega_{e}^{\prime }x_{e}^{\prime }=0.207$ ${cm}^{-1}$.

Figure 21 and Figure 22 present the LIF excitation spectra recorded using transitions corresponding to the first ($B^{3}1,\upsilon_{B}^{\prime \prime }⟵X^{1}\Sigma_{1}^{+},\upsilon_{X}=0$) and second steps ($E^{3}\Sigma_{1}^{+}⟵B^{3}1,\upsilon_{B}^{\prime \prime }=0–4$) of the OODR process, respectively. It is necessary to emphasize that in order to characterize the $E^{3}\Sigma_{1}^{+}$-state outer well and the potential barrier, five $\upsilon_{B}^{\prime \prime }$ values were chosen as origins for the second-step excitation (see Figure 21). This assured very thorough and complete probing of the $E^{3}\Sigma_{1\;out}^{+}$ outer well and neighbouring barrier via bound⟵bound and free⟵bound transitions to different parts of the $E^{3}\Sigma_{1}^{+}$-state potential.

A closer look at Figure 22 reveals a series of bound⟵bound and free⟵bound LIF excitation spectra recorded using the $E^{3}\Sigma_{1}^{+}⟵B^{3}1,\upsilon_{B}^{\prime \prime }$ transition and originating from $\upsilon_{B}^{\prime \prime }=0–4$.

Bound⟵bound transitions probe the vibrational energy structure in the $E^{3}\Sigma_{1\;out}^{+}$ well, whereas free⟵bound transitions terminate on the outer wall of the potential barrier above the dissociation limit (refer to Figure 4) and provide information on the barrier itself, i.e., its position, height, and, to a certain degree, its shape.

This unique opportunity to simultaneously record both kinds of transitions is due to the relative positions of the $E^{3}\Sigma_{1}^{+}$- and $B^{3}1$-state potentials. Interpretation of LIF excitation spectra of $\upsilon_{E}^{\prime }⟵\upsilon_{B}^{\prime \prime }$ bound⟵bound transitions is straightforward, but interpretation of free⟵bound transitions originating from a given $\upsilon_{B}^{\prime \prime }$ needs an explanation. As can be seen in Figure 22b, the spectra of free⟵bound transitions show an undulated structure (oscillations); however, they do not correspond strictly to the so-called reflection nature, as classified by Tellinghuisen [211], according to which they should ‘reflect’ the radial probability distribution in the initial $\upsilon_{B}^{\prime \prime }$ level, conserving the peak and node count (i.e., $\upsilon_{B}^{\prime \prime }+1$) of the $\psi_{\upsilon_{B}^{\prime \prime }}$ initial $\upsilon_{B}^{\prime \prime }$-level wavefunction. The reflection spectra (unlike the second type—interference spectra) are frequently recorded in dispersed emission (also called dispersed fluorescence) from a previously excited $\upsilon^{\prime }$ level to the repulsive branch of, most frequently, the ground-state $\upsilon^{\prime \prime }$ level, giving an opportunity to determine the emitting $\upsilon^{\prime }$-level quantum number (this type of spectra is associated with the ‘Condon internal diffraction’ phenomenon introduced by E. U. Condon [212]) (see examples for MeNg molecules reported in Refs. [213,214]). However, in this particular case of the $E^{3}\Sigma_{1}^{+}{⟵B}^{3}1,\upsilon_{B}^{\prime \prime }$ excitation, free⟵bound transitions terminate on the upper-state repulsive part (i.e., outer wall) of the potential barrier (causing immediate dissociation of the molecule), providing a rare opportunity for its characterization.

As far as the spectra of bound⟵bound transitions are concerned (Figure 22a), the entire $\upsilon_{E_{out}}^{\prime }$-level vibrational structure in the $E^{3}\Sigma_{1}^{+}$-state outer well was probed in the experiment [12]; therefore, the B-S plot analysis (Figure 22c) turned out to be a very reliable tool to determine $\omega_{e}^{\prime }$, $\omega_{e}^{\prime }x_{e}^{\prime },$ and $\upsilon_{max}^{\prime }=\frac{\omega_{e}^{\prime }}{2\omega_{e}^{\prime }x_{e}^{\prime }}-\frac{1}{2}=10$, i.e., the maximum number of $\upsilon_{E_{out}}^{\prime }$ in the outer well. Moreover, as the B-S plot is linear, it was straightforward to propose a Morse representation for the $E^{3}\Sigma_{1\;out}^{+}$ potential well. In addition, with respect to the earlier analysis by Ref. [15] (see in Section 5.2.2 and Figure 18), here, an additional $\upsilon_{E_{out}}^{\prime }$ (previously missing) component in the spectrum was recorded (see Figure 22a—marked with asterisk). Therefore, the whole approach delivered new vibrational characterization of the $E^{3}\Sigma_{1\;out}^{+}$ outer well, which was compared with that of Ref. [15] presented in Figure 20. The new $\upsilon_{E_{out}}^{\prime }$ assignment also affected the determination of the $R_{e\;out}^{\prime }$ presented in Ref. [15] (see Figure 19 and Table 1).

When analysing the spectra of free⟵bound transitions (Figure 22b), one may notice their departures from pure reflection character. Following Duval et al. [24], this can be interpreted as extraneous oscillations in the spectra, which possess an interference structure that results from the occurrence of a potential barrier. Also, an influence of lying above the dissociation limit last $\upsilon_{E_{in}}^{\prime }=19$ level, supported by the inner well and excited from the $B^{3}1,\;\upsilon_{B}^{\prime \prime }$ levels, is evident, and this vibrational component overlaps with bound free⟵transitions.

Additional information of the $E^{3}\Sigma_{1}^{+}$-state potential barrier is provided in the experiment and analysis presented in Ref. [13] (see Figure 16 in Section 5.2.1) as the LIF excitation spectrum recorded using the $E^{3}\Sigma_{1}^{+}⟵A^{3}\Pi_{0^{+}},\upsilon_{A}^{\prime \prime }=6$ transitions that includes a profile of free⟵bound transitions terminating at the repulsive inner wall of the potential barrier. As already stated, simulation of that profile provided an estimation of the height of the barrier (see the inset in Figure 15). Determination of the height of the barrier from the $E^{3}\Sigma_{1}^{+}{⟵B}^{3}1,\upsilon_{B}^{\prime \prime }$ free⟵bound transitions [12] corroborated the previous result [13] and specified the value as $E_{b}^{\prime }$ = 27 ${cm}^{-1}$ (see Table 1).

**Figure 23:**
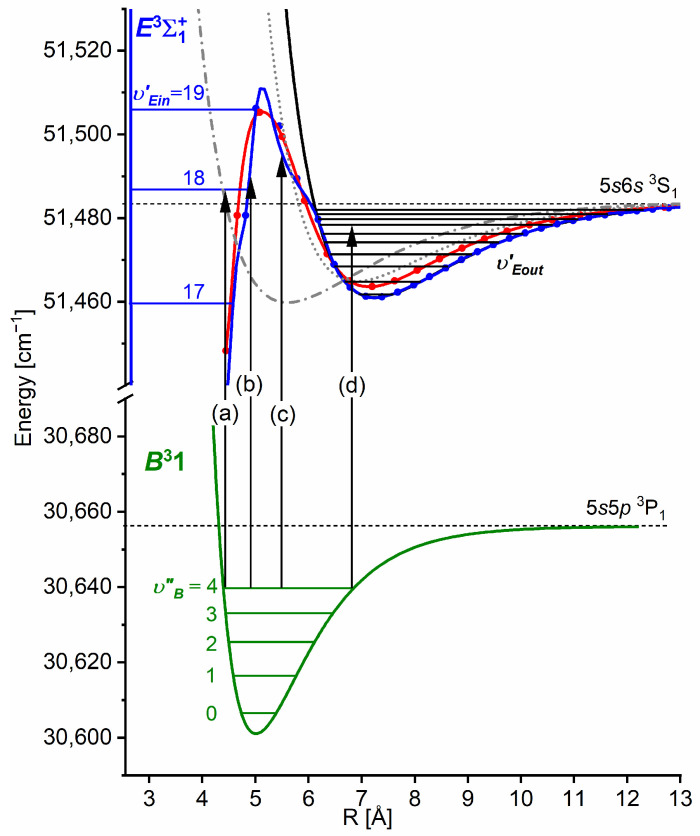
Comparison of the CdAr interatomic potential of the $E^{3}\Sigma_{1}^{+}({5s6s\;}^{3}S_{1})$ Rydberg-state outer well representations close to the dissociation limit, being the result of series of experiments using the OODR excitation method as follows: very first investigation by Koperski and Czajkowski [17] (grey dashed-dotted line), result of Urbańczyk and Koperski [15] (grey dotted line), and the most recent result of Sobczuk et al. [12] (black solid line)—also see Table 1. Note: for the inner potential well, the representations refer to Figure 15. Part of the whole $E^{3}\Sigma_{1}^{+}$-state potential representation in the vicinity of the potential barrier as the result of Ref. [12] is also shown (blue line and points)—see the text for details. The ab initio-calculated potential by Krośnicki et al. [4] is shown for comparison (red line and points). The potential of the $B^{3}1$ intermediate state [16] used in the OODR process is also drawn (green solid line). Three ways to execute the excitation using the $E^{3}\Sigma_{1}^{+}{⟵B}^{3}1,\upsilon_{B}^{\prime \prime }=4$ transitions are depicted with vertical arrows as follows: (**a**) quasi-bound⟵bound to the $E^{3}\Sigma_{1\;in}^{+}$, (**b**,**c**) free⟵bound terminating at the inner and outer repulsive walls of the potential barrier, respectively, and (**d**) bound⟵bound to the $E^{3}\Sigma_{1\;out}^{+}$.

The real success of the presented studies relied on the first-time determination of entire $E^{3}\Sigma_{1}^{+}$-state interatomic potential that consisted of inner well, potential barrier, and outer well. This was achieved entirely using experimental data obtained from LIF excitation spectra of bound⟵bound and free⟵bound transitions and their simulations assuming a pointwise model potential consisting of three parts corresponding to the three regions—this potential is shown in Figure 23 and consists of the following:

- $E^{3}\Sigma_{1\;in}^{+}$, deeper inner well—for R<4.56 Å—adopted as the result of IPA method [13].
- Potential barrier—for 4.82 Å<R<5.91 Å—modification of the ab initio-calculated potential [4].
- $E^{3}\Sigma_{1\;out}^{+}$, shallower outer well—for R>6.38 Å—represented by a Morse function [12] converted to the pointwise form combined using a cubic spline method. To obtain a simulation of the free⟵bound profiles that satisfactorily reproduce that recorded in the experiment, slight modifications were introduced as follows: 0.01-Å and 0.16-Å shifts along the *R* axis of all ab initio points [4] used to construct the barrier and the IPA-based $E^{3}\Sigma_{1\;in}^{+}$-state potential [13], respectively.

In Figure 23, the $E^{3}\Sigma_{1}^{+}$-state potential of Ref. [12] is compared with those representations that were obtained earlier with limited sets of experimental data [15,17].

### 5.3. Perspectives: Bound→Free Emission after OODR Excitation of the CdAr (and ZnAr) Rydberg State—Characterization of Lower-Lying ‘Dark’ States or States Inaccessible in Direct Excitation from the Ground State

**Figure 24:**
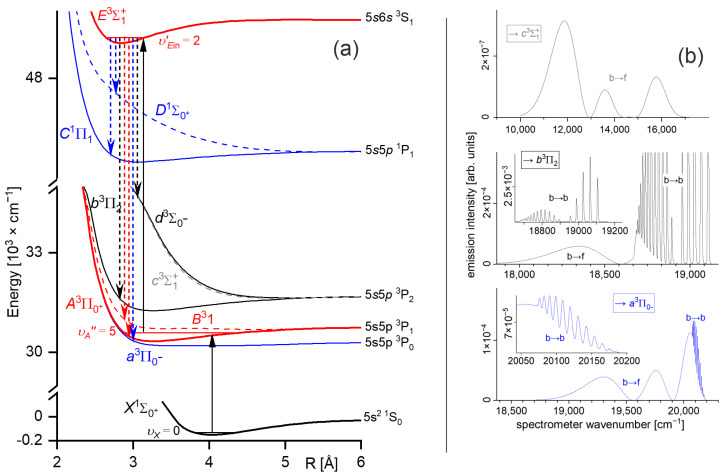
(**a**) Ab initio-calculated interatomic potentials of CdAr electronic energy states [4] used to illustrate the OODR process of the excitation of $E^{3}\Sigma_{1}^{+},\upsilon_{E_{in}}^{\prime \prime }=2$ from $X^{1}\Sigma_{1}^{+},\upsilon_{X}=0$ via $A^{3}\Pi_{0^{+}},\upsilon_{A}^{\prime \prime }=5$ (vertical solid arrows) followed by emission to several lower-lying molecular states, allowing for the characterization of their potentials using bound→free and bound→bound transitions. (**b**) Examples of simulated dispersed emission spectra [205,206,209] with bound→free (b→f) and bound→bound (b→b) transitions from $E^{3}\Sigma_{1}^{+},\upsilon_{E_{in}}^{\prime \prime }=2$ to $c^{3}\Sigma_{1}^{+}({5s5p\;}^{3}P_{2})$, $b^{3}\Pi_{2}({5s5p\;}^{3}P_{2})$ and $a^{3}\Pi_{0^{-}}({5s5p\;}^{3}P_{0})$ electronic ‘dark’ states, which would serve as data to determine their PECs [202].

The higher-lying Rydberg electronic energy state can be exploited, after its excitation, as a source of emission to lower-lying states that (1) because of the selection rules in excitation, cannot be excited from the ground state (consequently called ‘dark’ states) or (2) because of their relative position, certain parts of their PECs can be probed only in emission (not in excitation). Figure 24 shows a number of electronic energy states of CdAr lying below the $E^{3}\Sigma_{1}^{+}({5s6s\;}^{3}S_{1})$ Rydberg state. Therefore, after excitation of a selected $E^{3}\Sigma_{1}^{+}$-state $\upsilon_{E}^{\prime }$ level, a corresponding number of emission channels occurs and the emission terminates on those states, giving rise to bound→free or bound→bound transitions, depending where the emission terminates, on the repulsive or bound part of the respective molecular potential, respectively. The analysis of the dispersed emission spectra (recorded with a spectrometer or monochromator) allows for determining the shape of the potential on which the emission terminated.

The approach presented above for the $E^{3}\Sigma_{1}^{+}$ Rydberg state in CdAr as a source of multichannel emission after the OODR process down to the ‘dark’ electronic states was also demonstrated for the ^1^$1({4s4d\;}^{1}D_{2})$ Rydberg state in ZnAr [202]. A more versatile study, i.e., simulations supported by experiment, was performed for the $C^{3}\Sigma_{1}^{+}({6s7s\;}^{3}S_{1})$ Rydberg state of HgAr by Duval et al. [24].

### 5.4. Improved Determination of the Inner and Outer Wells of the $E^{3}\Sigma_{1}^{+}$-State Potential in CdKr

By 2018, the $E^{3}\Sigma_{1}^{+}({5s6s\;}^{3}S_{1})$ Rydberg double-well state potential of the CdKr molecule was investigated as a whole only once in 2004 [20]. In that study, pronounced $\upsilon_{E_{in}}^{\prime }=0–23⟵\upsilon_{A}^{\prime \prime }=9$ and $\upsilon_{E_{in}}^{\prime }=18–27⟵\upsilon_{B}^{\prime \prime }=1$ progressions to the vibrational energy structure of the inner well were recorded using the $E^{3}\Sigma_{1in}^{+}⟵A^{3}\Pi_{0^{+}}$ and ${E^{3}\Sigma_{1\;in}^{+}⟵B}^{3}1$ transitions, respectively. Also, $\upsilon_{E_{out}}^{\prime }=0–6⟵\upsilon_{B}^{\prime \prime }=1$ progression to the outer well and fraction of free⟵bound ($\upsilon_{B}^{\prime \prime }=1$) transitions to the repulsive wall of the potential barrier were recorded using the ${E^{3}\Sigma_{1\;out}^{+}⟵B}^{3}1$ transition. Analysis of the experimental spectra allowed for limited characterization of the inner and outer $E^{3}\Sigma_{1}^{+}$-state potential wells. Both potential wells were individually represented with a corresponding Morse function stating, at the same time, that Morse representations were not adequately close to the dissociation limit or the potential barrier.

In the next study, the ${E^{3}\Sigma_{1\;in}^{+},\upsilon }_{E_{in}}^{\prime }=21⟵{A^{3}\Pi_{0^{+}},\upsilon }_{A}^{\prime \prime }=9$ vibrational band was recorded and simulated, focusing on its isotopic and rotational structures [16]. Simulation of the band [206] allowed for performing a limited rotational characterization by determining the respective rotational constants (see Table 1).

**Figure 25:**
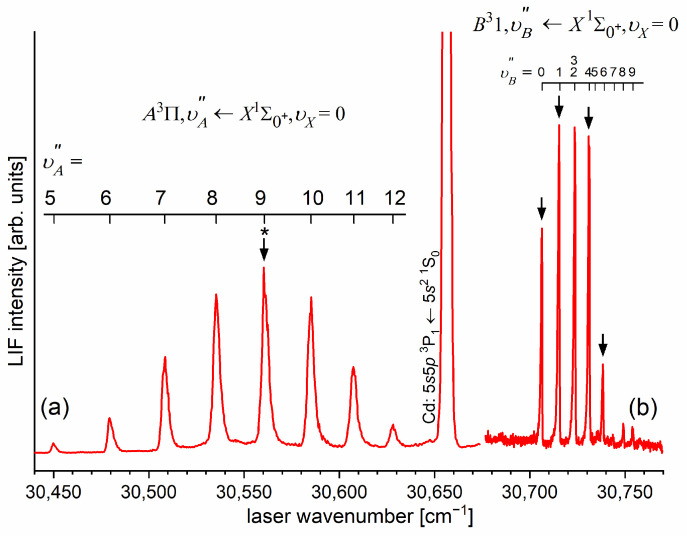
LIF excitation spectra recorded using the (**a**) $A^{3}\Pi_{0^{+}},\upsilon_{A}^{\prime \prime }⟵X^{1}\Sigma_{1}^{+},\upsilon_{X}=0$ [215] and (**b**) $B^{3}1,\upsilon_{B}^{\prime \prime }⟵X^{1}\Sigma_{1}^{+},\upsilon_{X}=0$ [106] transitions in CdKr, both being the first steps of the excitation in OODR processes, i.e., $E^{3}\Sigma_{1\;in}^{+},\upsilon_{E_{in}}^{\prime }⟵A^{3}\Pi_{0^{+}},\upsilon_{A}^{\prime \prime }⟵X^{1}\Sigma_{1}^{+},\upsilon_{X}=0$ and $E^{3}\Sigma_{1}^{+},\upsilon_{E}^{\prime }⟵B^{3}1,\upsilon_{B}^{\prime \prime }⟵X^{1}\Sigma_{1}^{+},\upsilon_{X}=0,$ that allowed for investigating the inner $E^{3}\Sigma_{1\;in}^{+}$ and outer $E^{3}\Sigma_{1\;out}^{+}$ potential wells. Arrows show the $\upsilon_{A}^{\prime \prime }=9$ and $\upsilon_{B}^{\prime \prime }=0,\;1,\;4,\;6$ that were used as origins for the second-step OODR transition. The asterisk depicts the $\upsilon_{A}^{\prime \prime }=9⟵\upsilon_{X}=0$ vibrational band recorded with higher resolution and used, among others, in the isotopologue selection experiment (see Section 5.4). Note: irregular, i.e., non-Morse, behaviour of $\upsilon_{B}^{\prime \prime }$ levels is due to the double-well character of the $B^{3}1$-state potential [106].

**Figure 26:**
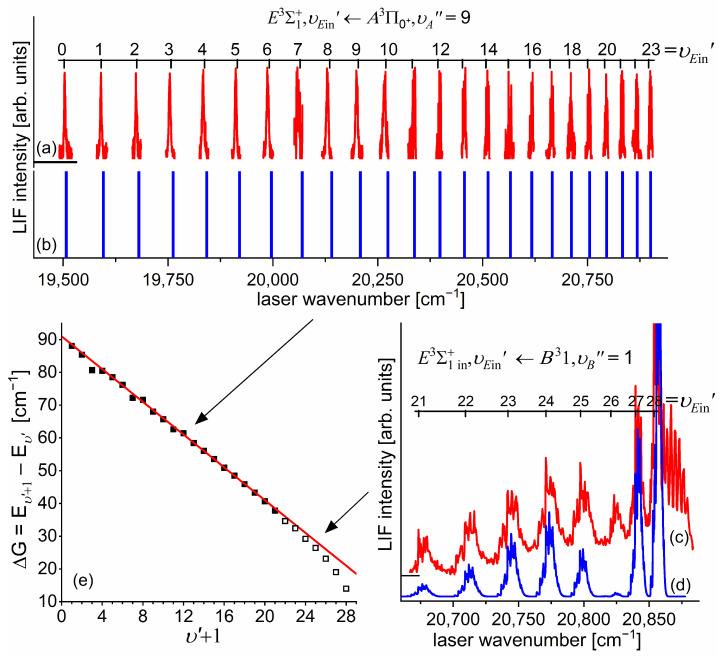
Experimental LIF excitation spectra terminating at the vibrational energy structure of the $E^{3}\Sigma_{1}^{+}$-state inner potential well in CdKr, recorded using (**a**) $E^{3}\Sigma_{1\;in}^{+},\upsilon_{E_{in}}^{\prime }⟵A^{3}\Pi_{0^{+}},\upsilon_{A}^{\prime \prime }=9$ and (**c**) $E^{3}\Sigma_{1\;in}^{+},\upsilon_{E_{in}}^{\prime }⟵B^{3}1,\upsilon_{B}^{\prime \prime }=1$ transitions, with intensities of the spectrum in (**a**) normalized because of the large spectral region in which the total spectrum for $\upsilon_{E_{in}}^{\prime }$ was recorded (different dye lasers and carrier gas pressures were applied). (**b**) Simulation of positions only and (**d**) simulation of positions and intensities performed assuming the $E^{3}\Sigma_{1\;in}^{+}$ potential representation as the result of the IPA method using the (**b**) LEVEL [205] and (**d**) LEVEL and PGOPHER [206] programs, assuming (**d**) $T_{rot}=3\;K$ and $\Delta_{L}=\Delta_{G}=0.15\;cm^{-1}$. (**e**) B-S plot for the $\upsilon_{E_{in}}^{\prime }=0–21⟵\upsilon_{A}^{\prime \prime }=6$ (full squares) and $\upsilon_{E_{in}}^{\prime }=22–28⟵\upsilon_{B}^{\prime \prime }=1$ (empty squares) progressions, showing distinct nonlinearity for $\upsilon_{E_{in}}^{\prime }\geq 24$. The size of the points in the plot equals the error bars for ΔG.

In 2019, a very thorough and as complete as possible investigation of the CdKr inner $E^{3}\Sigma_{1\;in}^{+}$ and outer $E^{3}\Sigma_{1\;out}^{+}$ potential wells was conducted by performing a series of experiments in a relatively large spectral region and recording LIF excitation spectra that probed the vibrational and isotopic structures of both $E^{3}\Sigma_{1\;in}^{+}$ and $E^{3}\Sigma_{1\;out}^{+}$ in different ranges of $\upsilon_{E_{in}}^{\prime }$ and $\upsilon_{E_{out}}^{\prime }$, respectively [19]. Both the $A^{3}\Pi_{0^{+}}$ and $B^{3}1$ intermediate states were used in the OODR process (see the $A^{3}\Pi_{0^{+}},\upsilon_{A}^{\prime \prime }⟵X^{1}\Sigma_{1}^{+},\upsilon_{X}=0$ [215] and (b) $B^{3}1,\upsilon_{B}^{\prime \prime }⟵X^{1}\Sigma_{1}^{+},\upsilon_{X}=0$ [106] transitions used in OODR first-step excitation—Figure 25) and the following transitions were recorded:

- To the inner potential well (see Figure 26a,c):

$E^{3}\Sigma_{1\;in}^{+},\upsilon_{E_{in}}^{\prime }=0–23⟵A^{3}\Pi_{0^{+}},\upsilon_{A}^{\prime \prime }=9$;

$E^{3}\Sigma_{1\;in}^{+},\upsilon_{E_{in}}^{\prime }=21–28⟵B^{3}1,\upsilon_{B}^{\prime \prime }=1$.

**Figure 27:**
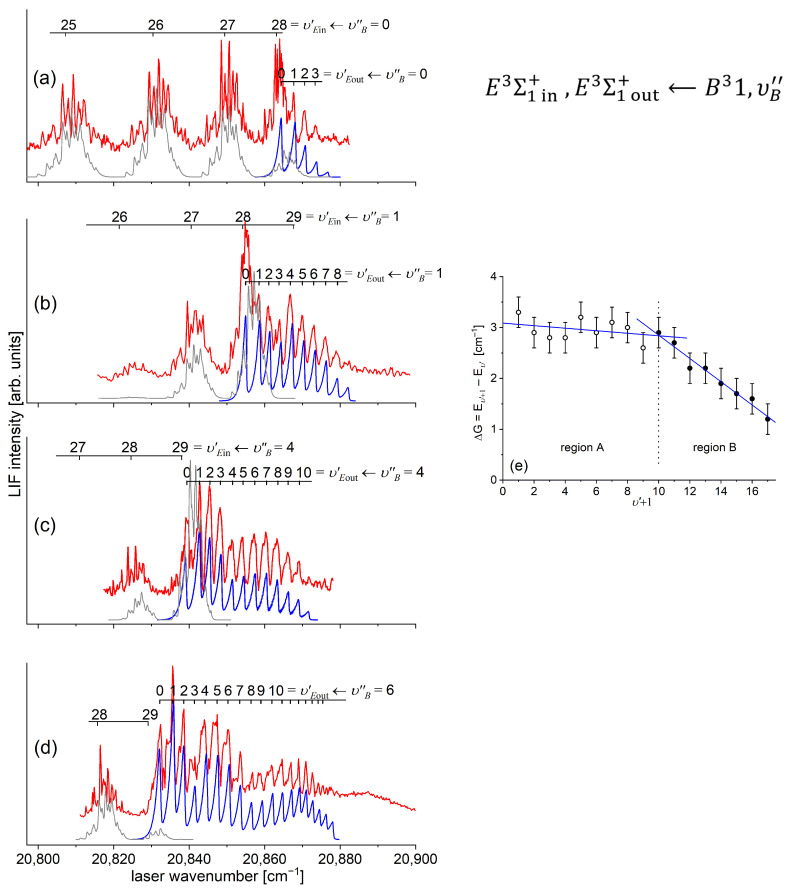
LIF excitation spectra (red traces) terminating at the $\upsilon_{E_{out}}^{\prime }$ vibrational energy structure of the $E^{3}\Sigma_{1}^{+}$-state outer well and the most upper-lying $\upsilon_{E_{in}}^{\prime }$ levels of the $E^{3}\Sigma_{1}^{+}$-state inner well in CdKr, recorded using $E^{3}\Sigma_{1\;out}^{+},\upsilon_{E_{out}}^{\prime }⟵B^{3}1,\upsilon_{B}^{\prime \prime }$ and $E^{3}\Sigma_{1\;in}^{+},\upsilon_{E_{in}}^{\prime }⟵B^{3}1,\upsilon_{B}^{\prime \prime }$, respectively, starting at (**a**) $\upsilon_{B}^{\prime \prime }=0$, (**b**) $\upsilon_{B}^{\prime \prime }=1$, (**c**) $\upsilon_{B}^{\prime \prime }=4$, and (**d**) $\upsilon_{B}^{\prime \prime }=6$. Simulations of the $E^{3}\Sigma_{1\;out}^{+}⟵B^{3}1$ and $E^{3}\Sigma_{1\;in}^{+}⟵B^{3}1$ spectra (blue and grey traces, respectively) performed using the LEVEL [205] and PGOPHER [206] programs in which $E^{3}\Sigma_{1\;out}^{+}$, $E^{3}\Sigma_{1\;in}^{+}$ and $B^{3}1$-state representations were taken from Refs. [19,106], and $T_{rot}=3\;K$ and $\Delta_{L}=\Delta_{G}=0.15\;cm^{-1}$ were assumed. (**e**) B-S plot drawn for the $E^{3}\Sigma_{1\;out}^{+}$ potential well in CdKr based on the $E^{3}\Sigma_{1\;out}^{+}{,\upsilon }_{E_{out}}^{\prime }⟵B^{3}1,\upsilon_{B}^{\prime \prime }=6$ transition shown in (**d**), in which strong nonlinearity is present, and two regions, A ($\upsilon_{E_{out}}^{\prime }=0–8$) and B ($\upsilon_{E_{out}}^{\prime }=9–16$), each of linear behaviour, can be extracted.

- To the outer potential well (see Figure 27a–d):

$$E^{3}\Sigma_{1\;out}^{+},\upsilon_{E_{out}}^{\prime }=1–3⟵B^{3}1,\upsilon_{B}^{\prime \prime }=0;\;E^{3}\Sigma_{1\;out}^{+},\upsilon_{E_{out}}^{\prime }=1–8⟵B^{3}1,\upsilon_{B}^{\prime \prime }=1;\;E^{3}\Sigma_{1\;out}^{+},\upsilon_{E_{out}}^{\prime }=0–10⟵B^{3}1,\upsilon_{B}^{\prime \prime }=4;\;E^{3}\Sigma_{1\;out}^{+},\upsilon_{E_{out}}^{\prime }=0–17⟵B^{3}1,\upsilon_{B}^{\prime \prime }=6.$$

Figure 26a,c present LIF excitation spectra recorded using transitions, terminating at the energy structure of $\upsilon_{E_{in}}^{\prime }$ levels probed from both the $A^{3}\Pi_{0^{+}},\upsilon_{A}^{\prime \prime }=9$ and $B^{3}1,\upsilon_{B}^{\prime \prime }=1$ intermediate levels, respectively, this way extending accessibility of the excitation to all $\upsilon_{E_{in}}^{\prime }$ levels (from 0 to 28) supported by the $E^{3}\Sigma_{1\;in}^{+}$ well. Before simulating both spectra, it was concluded that the $E^{3}\Sigma_{1\;in}^{+}$ well characterizes itself with a distinct non- Morse behaviour close to the dissociation limit. As can be seen in Figure 26e, the B-S plot reveals a non-linear component for $\upsilon_{E_{in}}^{\prime }\geq 24$ that prevents the representation of the $E^{3}\Sigma_{1\;in}^{+}$ well with a Morse function. Therefore, to solve this problem and find a proper representation for $E^{3}\Sigma_{1\;in}^{+}$, the IPA methodology [207] was employed. This approach allowed for arriving at simulations, presented in Figure 26b,d, that reconstruct both spectra satisfactorily as far as positions of the components in $\upsilon_{E_{in}}^{\prime }⟵\upsilon_{A}^{\prime \prime }=9$ and $\upsilon_{E_{in}}^{\prime }⟵\upsilon_{B}^{\prime \prime }=1$ progressions are concerned, including their partly resolved isotopic structure for high $\upsilon_{E_{in}}^{\prime }$. As for the $E^{3}\Sigma_{1}^{+}⟵A^{3}\Pi_{0^{+}}$ and $E^{3}\Sigma_{1}^{+}⟵B^{3}1$ transitions in CdKr, in the simulations, only FC-F factors were taken into account, concluding with relatively good agreement between simulated and recorded intensities of vibrational components including their isotopic structure.

The $E^{3}\Sigma_{1}^{+}$-state outer well was probed using LIF excitation spectra recorded using the $E^{3}\Sigma_{1\;out}^{+},\upsilon_{E_{out}}^{\prime }⟵B^{3}1,\upsilon_{B}^{\prime \prime }=0,\;1,\;4$, 6 second-step transitions of the OODR process (note that in the first study [20], only one $\upsilon_{E_{out}}^{\prime }⟵\upsilon_{B}^{\prime \prime }=1$ progression was recorded with poor spectral resolution). The spectra are shown in Figure 27a–d. As can be easily concluded, $\upsilon_{E_{out}}^{\prime }⟵\upsilon_{B}^{\prime \prime }$ progressions of the $E^{3}\Sigma_{1\;out}^{+}⟵B^{3}1$ transition overlap with the $\upsilon_{E_{in}}^{\prime }⟵\upsilon_{B}^{\prime \prime }$ progressions of the $E^{3}\Sigma_{1\;in}^{+}⟵B^{3}1$ transition, which makes the analysis more difficult. However, two separate simulations for each $\upsilon_{B}^{\prime \prime }$ with assumed representations of the $E^{3}\Sigma_{1\;out}^{+}$ and $E^{3}\Sigma_{1\;in}^{+}$ potential wells as those derived from the IPA methodology (for $E^{3}\Sigma_{1\;in}^{+},$ the representation derived from the spectrum shown in Figure 26 was used), and the $B^{3}1$-state representation from Ref. [106] revealed satisfactory reconstruction of the recorded spectra. It is important to note that the simulations were performed without a TDM function for the transition, and the intensities of vibrational components were assumed to have arbitrary values. Additionally, as the $B^{3}1$-state potential also possesses a double-well structure (which was concluded from ab initio calculation [40] and experimental studies [106]), a small change in its shape would have a significant impact on the shape of $\upsilon_{B}^{\prime \prime }$-level wavefunctions and, consequently, on the intensities of $\upsilon_{E_{out}}^{\prime }⟵\upsilon_{B}^{\prime \prime }$ and $\upsilon_{E_{in}}^{\prime }⟵\upsilon_{B}^{\prime \prime }$ transitions.

**Figure 28:**
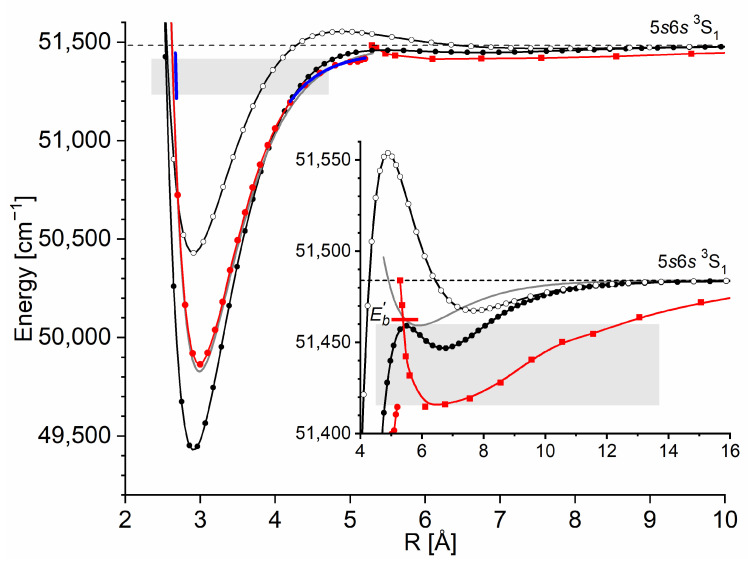
Representations of the $E^{3}\Sigma_{1}^{+}({5s6s\;}^{3}S_{1})$ Rydberg state potential of CdKr with an inset showing details of the potential barrier and outer potential well close to the dissociation limit. $E^{3}\Sigma_{1\;in}^{+}$ (red line and circles) and $E^{3}\Sigma_{1\;out}^{+}$ (red line and squares) potential well representations as results of the IPA methodology and analysis of the spectra in Figure 26a,c and Figure 27d, respectively. A Morse representation of $E^{3}\Sigma_{1\;in}^{+},$ being the result of the B-S plot analysis in Figure 26e, is also presented (blue solid line). The experimentally determined representations are compared with two results of ab initio calculations [216], showing their considerable difference, including those from 2008 performed by Krośnicki and collaborators [39,40] (black line and empty circles) and those most recently performed by Krośnicki et al. [208] (black line and full circles). The estimated height of the potential barrier $E_{b}^{\prime }$ is shown as a red horizontal line. For comparison, the result of the first experimental study [20] (grey solid lines) is included. The ranges of $\upsilon_{E_{in}}^{\prime }$ and $\upsilon_{E_{out}}^{\prime }$ excited from $B^{3}1,\upsilon_{B}^{\prime \prime }$ and analysed in [19] are shown (grey rectangles).

Figure 28 presents a representation of the $E^{3}\Sigma_{1}^{+}$ Rydberg state potential in CdKr that was derived from the LIF excitation spectra of the $E^{3}\Sigma_{1\;in}^{+},\upsilon_{E_{in}}^{\prime }⟵A^{3}\Pi_{0^{+}},\upsilon_{A}^{\prime \prime }=9$ and $E^{3}\Sigma_{1\;in}^{+},\upsilon_{E_{in}}^{\prime }⟵B^{3}1,\upsilon_{B}^{\prime \prime }=1$ transitions (see Figure 26a,c), and the $E^{3}\Sigma_{1\;out}^{+},\upsilon_{E_{out}}^{\prime }⟵B^{3}1,\upsilon_{B}^{\prime \prime }=6$ transition (see Figure 27d) using the IPA methodology. The approach was justified by nonlinearity in the B-S plots that were constructed for $\upsilon_{E_{in}}^{\prime }$ (Figure 26e) and $\upsilon_{E_{out}}^{\prime }$ (Figure 27e) vibrational energy structures supported by $E^{3}\Sigma_{1}^{+}$-state inner and outer potential wells, respectively, and the immediate conclusion that neither the inner nor outer well can be represented by a Morse function. The IPA representation is compared with two results of ab initio calculations, including those from 2008 [39,40] and the most recent [208], showing their disagreement with the experimental result and calling for additional studies using both calculational and experimental approaches (note, the most recent ab initio result shows noticeable improvement as compared to the earlier one).

Special attention has to be paid to all three aspects of the problem as follows: the depth of two potential wells and the height of the potential barrier. As can be seen in Figure 28, the experimentally determined depth of the inner well [19] falls between both ab initio results, whereas the depth of the outer well is significantly larger than both ab initio-calculated results. The comparison is supplemented with the first experimental result [20], which for the inner well is close to the IPA potential (Table 3 in Ref. [19]), but for the outer well, not surprisingly, it departs largely from that obtained using the IPA method (Table 5 in Ref. [19]), as obtained from a smaller data set as argued above (for detailed spectroscopic data see Table 1). Unfortunately, from the recorded spectra, it was not possible to conclude either the shape of the $E^{3}\Sigma_{1}^{+}$-state potential near the barrier nor the shape of barrier itself. However, the highest $\upsilon_{E_{out}}^{\prime }=17$ observed in the $E^{3}\Sigma_{1\;out}^{+},\upsilon_{E_{out}}^{\prime }⟵B^{3}1,\upsilon_{B}^{\prime \prime }=6$ spectrum possesses the energy that is 3 $cm^{-1}$ above the top of the barrier, which was recently ab initio-calculated [208]. This suggests that the actual height of the barrier is somewhat larger. This can be additionally supported by the presence of a broad band (see Figure 27d) spanning over approx. 20 $cm^{-1}$ (i.e., between 51,462 $cm^{-1}$ and 51,483 $cm^{-1}$ with respect to $X^{1}\Sigma_{1}^{+}$-state dissociation energy), which was interpreted based on the hypothesis of unresolved $\upsilon_{E_{out}}^{\prime }⟵\upsilon_{B}^{\prime \prime }=6$ vibrational transitions supported by the $E^{3}\Sigma_{1}^{+}$-state potential above the barrier and below the dissociation limit, correlating with the $\left({5s6s\;}^{3}S_{1}\right)$ Cd asymptote.

### 5.5. Practical Method for Isotopologue Selection Using OODR—The Cases of CdKr and CdAr

In general, isotopologue-selective excitation could be possible provided that the isotopic shift in the chosen vibrational components is sufficiently large. This has been demonstrated with the proper choice of a laser wavenumber with sufficiently narrow spectral bandwidth in OODR first-step excitation, which can lead to a isotopologue-selective excitation in the $A^{3}\Pi_{0^{+}}$ intermediate state. Thanks to this approach, selected isotopologues could be excited, contributing subsequently to the intensity of LIF signal originating from the $E^{3}\Sigma_{1}^{+}$ final state after OODR second-step excitation.

In Section 5.2.1, isotopologue selection in excitation was suggested in the case of the $A^{3}\Pi_{0^{+}},\upsilon_{A}^{\prime \prime }=6⟵X^{1}\Sigma_{1}^{+},\upsilon =0$ first-step OODR transition in CdAr, where only two isotopologues, ^116^${Cd}^{40}Ar$ and ^114^${Cd}^{40}Ar$, were excited, with the former predominating (see Figure 17B). Then, the result of the isotopologues selection was demonstrated in the subsequent $E^{3}\Sigma_{1in}^{+},\upsilon_{E_{in}}^{\prime }=2,5,11,17⟵A^{3}\Pi_{0^{+}},\upsilon_{A}^{\prime \prime }=6$ second-step OODR transitions (see Figure 17A), showing the possibility of increasing the resolution of rotational structures in the recorded spectra because of the limited number of isotopologues excited. Note that CdAr possesses 6 out of  ${24\;}^{A_{Cd}}{Cd}^{A_{Ar}}Ar$ isotopologues with a considerable abundance of α, i.e., α>3% $A_{Cd}$ (and $A_{Ar}$ are Cd and Ar mass numbers, respectively).

**Figure 29:**
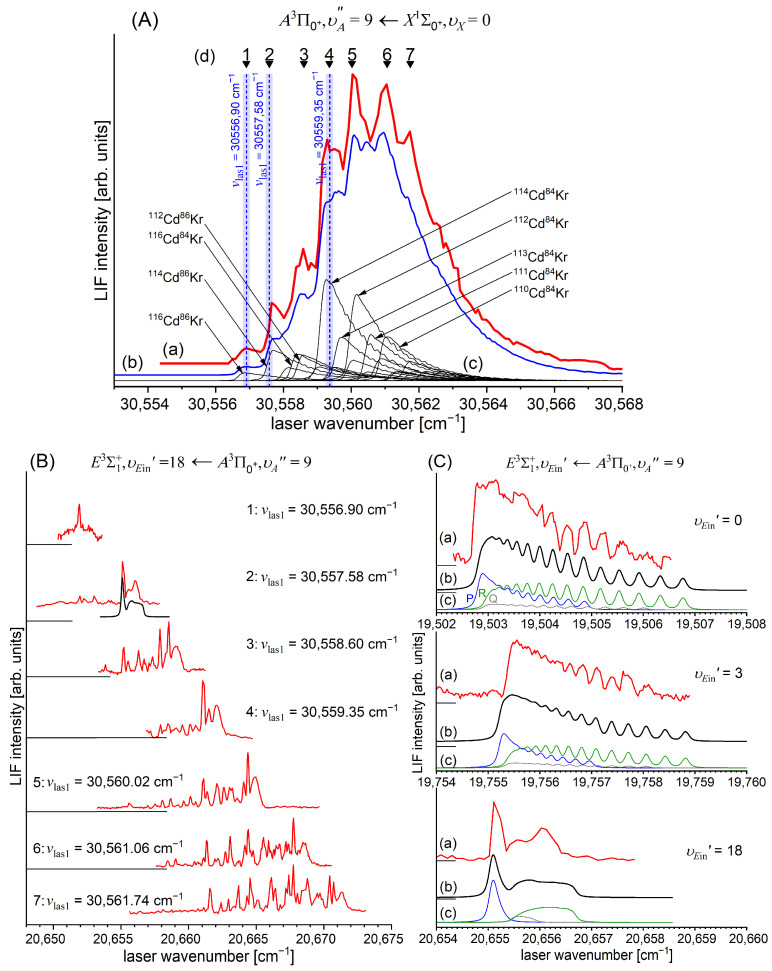
(**A**): (**a**) Profile of the LIF excitation spectrum recorded using the $A^{3}\Pi_{0^{+}},\upsilon_{A}^{\prime \prime }=9⟵X^{1}\Sigma_{1}^{+},\upsilon =0$ transition in CdKr. (**b**) Total simulation of the profile performed using the PGOPHER [206] program, assuming $T_{rot}=4\;K$ and $\Delta_{L}=\Delta_{G}=0.1\;cm^{-1}$ as well as $B_{\upsilon }$ and $D_{\upsilon }$ rotational constants, and transition energies calculated using the LEVEL [205] program for the $A^{3}\Pi_{0^{+}}$ [16] and $X^{1}\Sigma_{1}^{+}$-state [215] potential characteristics. (**c**) Individual contributions to (**b**) that originate from different CdAKrACdKr isotopologues with abundances larger than 1%. (**d**) Positions of the $\nu_{las\;1}$ laser wavenumber of OODR first-step excitation used in (**B**); examples of $\nu_{las\;1}\pm 0.1\;cm^{-1}$ are represented with blue vertical dashed lines and rectangles. (**B**): Profiles of LIF excitation spectra recorded using the $E^{3}\Sigma_{1in}^{+},\upsilon_{E_{in}}^{\prime }=18⟵A^{3}\Pi_{0^{+}},\upsilon_{A}^{\prime \prime }=9$ OODR second-step transition for different $\nu_{las\;1}\pm 0.1\;cm^{-1}$ depicted in (**A**)-(**d**), i.e., for different combinations of excited CdAKrACdKr isotopologues. For the simulation shown with the black line (in position-2), refer to (**C**). (**C**): (**a**) Profiles of LIF excitation spectra recorded using the $E^{3}\Sigma_{1in}^{+},\upsilon_{E_{in}}^{\prime }=0,3,18⟵A^{3}\Pi_{0^{+}},\upsilon_{A}^{\prime \prime }=9$ transitions in ^114^${Cd}^{86}Kr$ (with small admixture of ^116^${Cd}^{40}Kr$)—excitation in position-2: $\nu_{las\;1}=30,557.58\pm 0.1\;cm^{-1}$ shown in (**A**). (**b**) Simulations performed using the PGOPHER [206] program, assuming $J_{A\;min}^{\prime \prime }=0,\;J_{A\;max}^{\prime \prime }=15$, $T_{rot}=4\;K$ (for $\upsilon_{E_{in}}^{\prime }=0\;and\;3$), $T_{rot}=15\;K\;(for\upsilon_{E_{in}}^{\prime }=18$), and $\Delta_{L}=\Delta_{G}=0.1\;cm^{-1}$, as well as $B_{\upsilon }$ and $D_{\upsilon }$ rotational constants, and transition energies calculated using the LEVEL [205] program for the $E^{3}\Sigma_{1in}^{+}$ [16] and $A^{3}\Pi_{0^{+}}$-state [16] potential characteristics. (**c**) Simulated distributions of P-, Q-, and R-branch components depicted with a colour code as in part for $\upsilon_{E_{in}}^{\prime }=0$. The intensity of the Q-branch is damped, as concluded in Ref. [10].

The isotopologue selection in the excitation was also convincingly demonstrated for CdKr using the $A^{3}\Pi_{0^{+}},\upsilon_{A}^{\prime \prime }=9⟵X^{1}\Sigma_{1}^{+},\upsilon =0$ first- and, subsequent, $E^{3}\Sigma_{1in}^{+},\upsilon_{E_{in}}^{\prime }=18⟵A^{3}\Pi_{0^{+}},\upsilon_{A}^{\prime \prime }=9$ second-step OODR transitions. Figure 29A presents the $A^{3}\Pi_{0^{+}},\upsilon_{A}^{\prime \prime }=9⟵X^{1}\Sigma_{1}^{+},\upsilon =0$ profile with a considerably large isotopic shift (the shift increases with increasing $\upsilon_{A}^{\prime \prime }$) between CdAKrACdKr isotopologues. In Figure 29A, it is evident that the possibility of the excitation of selected isotopologues is most favourable for $\nu_{las\;1}=30,556.90\;cm^{-1},$ for which sole ^116^${Cd}^{86}Kr$ can be excited (see position-1 in Figure 29A). However, as the natural abundances of Cd and Kr were used in the experiment, the abundance of ^116^${Cd}^{86}Kr$ in the molecular beam is relatively small, i.e., α=1.3%, which resulted in a small signal-to-noise ratio (SNR) (see Figure 29B). The SNR is much better for other positions of $\nu_{las\;1}$. This is the case with $\nu_{las\;1}=30,557.58\;cm^{-1}$, in position-2, for which ^114^${Cd}^{86}Kr$ and a small admixture of ^116^${Cd}^{86}Kr$ are excited, or for $\nu_{las\;1}=30,559.35\;cm^{-1}$, in position-4, for the excitation of the most abundant ^114^${Cd}^{84}Kr$ with relatively very small admixtures of four other isotopologues. Note that CdKr possesses 22 out of 48 isotopologues with a considerable abundance of α (i.e., α>1%).

Simplification of LIF excitation spectra by reducing the number of excited isotopologues allows for partly resolving their rotational structure and facilitates their simulation. In some cases, such as that discussed here, for vibrational components recorded at the $E^{3}\Sigma_{1in}^{+},\upsilon_{E_{in}}^{\prime }⟵A^{3}\Pi_{0^{+}},\upsilon_{A}^{\prime \prime }$ second-step OODR transitions, it would be difficult (or even impossible) to achieve partial resolution of the rotational structure without applying the proposed approach. Using a sufficiently narrow spectral bandwidth laser in the first-step excitation also has an impact on the distribution of the populations of the excited $J_{A}^{\prime \prime }$ intermediate-state rotational levels. In other words, if an OODR first-step excitation laser $\nu_{las\;1}$ is sufficiently spectrally narrow, only a selected group of rotational levels $J_{A}^{\prime \prime }$ (from $J_{A\;min}^{\prime \prime }$ to $J_{A\;max}^{\prime \prime }$) in the $\upsilon_{A}^{\prime \prime }$ level is populated. It is evident that by narrowing the $\nu_{las\;1}$ spectral bandwidth, one can restrict the range of $J_{A}^{\prime \prime }$ in the intermediate state without manipulating $T_{rot}$. Consequently, the LIF excitation spectrum originated as the result of the $E^{3}\Sigma_{1in}^{+},\upsilon_{E_{in}}^{\prime }⟵A^{3}\Pi_{0^{+}},\upsilon_{A}^{\prime \prime }$ transition also contains a contribution only from the selected range of $J_{E_{in}}^{\prime \prime }$. An illustration of the problem is presented in Figure 29C, with profiles of LIF excitation spectra recorded using the $E^{3}\Sigma_{1in}^{+},\upsilon_{E_{in}}^{\prime }=0,3,18⟵A^{3}\Pi_{0^{+}},\upsilon_{A}^{\prime \prime }=9$ transitions in ^114^${Cd}^{86}Kr$ with small admixture of ^116^${Cd}^{86}Kr$ (see Figure 29A: $\nu_{las\;1}$ in position-2). Simulations of the profiles were performed with the assumption of strictly specified $T_{rot}$ and ranges of excited $J_{A}^{\prime \prime }$ from $J_{A\;min}^{\prime \prime }$ to $J_{A\;max}^{\prime \prime }$ (see figure caption).

It is evident that because of the isotopic shift between different isotopologues of the investigated CdNg molecules, even for relatively rich isotopic composition, by employing a laser with sufficiently small spectral bandwidth, it is possible to select one or few CdANgACdNg isotopologues in OODR first-step excitation. Limiting the number of isotopologues participating in the LIF signal as the result of OODR second-step excitation leads to the simplification of the recorded spectra originating from considerably dense rotational energy structure, allowing for partly resolving and simulating rotational profiles.

## 6. Particular Spectroscopic Applications of Rydberg Double-Well Electronic Energy States in Diatomic Molecules

A number of current spectroscopic applications of the results related to knowledge on potentials of the higher-excited (Rydberg) electronic energy states of homo- and heteroatomic molecules were reviewed in Section 2. Here, this review is concluded with two interesting applications that employ the HgAr vdW molecule in supersonic beams. The reason why the HgAr is mostly used in such demonstrations is the relative ease of production of Hg carried with Ar in a supersonic expansion (Hg temperature and Ar pressure corresponding to 400 K and 1–3 bar, respectively), which, in effect, constitutes an efficient source of ro-vibrationally cold HgAr mostly in $\left(\upsilon^{\prime \prime }=0,\;low\;J^{\prime \prime }\right)$ ground-state levels.

### 6.1. Spectroscopy of the ‘Dark’ c^*3*^*1* State of HgAr

**Figure 30:**
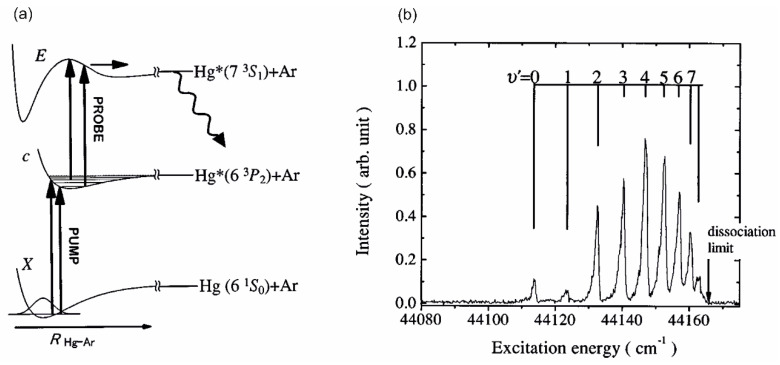
(**a**) Scheme of the double-resonance technique for the detection of the ‘dark’ intermediate $\upsilon^{\prime }$ vibrational levels of the $c^{3}1$ state in HgAr using probe laser excitation to the potential barrier in the $E^{3}1$ Rydberg state. (**b**) $c^{3}1\left(\upsilon^{\prime }\right)-\;X^{1}0^{+}(\upsilon^{\prime \prime }=0)$ excitation spectrum of the HgAr vdW complex plotted against the pump wavenumber (from Ref. [25], under the permission of the American Institute of Physics).

A very interesting application was presented by Amano and coworkers [25], aimed at the spectroscopy of the so-called ‘dark’ state (see Figure 30; notation of molecular states after Ref. [25]). This was the first observation of the $c^{3}1\left({6s6p\;}^{3}P_{2}\right)$ state of HgAr that, after excitation from the $X^{1}0^{+}({6s^{2}\;}^{1}S_{0})$ ground state, does not emit back; it is caused by the fact that it correlates with the ${(6s6p\;}^{3}P_{2})$ atomic level to which the dipole transition from the $\left({6s^{2}\;}^{1}S_{0}\right)$ level is strictly forbidden. This property partly transfers from atomic levels to the respective molecular states; $c^{3}1⟵\;X^{1}0^{+}$ excitation may occur, but emission is supressed because of the long fluorescence lifetime. To overcome this obstacle, the authors used a sequence of pump and probe laser pulses. The first, the pump laser pulse, tuned to the $c^{3}1(\upsilon_{c}^{\prime }=0–8)⟵\;X^{1}0^{+}(\upsilon_{X}^{\prime \prime }=0)$ progression, preceded the probe laser pulse, tuned to the $E^{3}1({6s7s\;}^{3}S_{1})⟵c^{3}1$ transition. The knowledge on the $E^{3}1$ Rydberg double-well state, which was acquired from the study by Duval and coworkers [24], allowed for time-adjusting the pump laser pulse so that it terminated at the outer wall of the potential barrier separating the two wells. Consequently, the molecule dissociated, and the $\left({6s7s\;}^{3}S_{1})⟶({6s6p\;}^{3}P_{J}),({6s^{2}\;}^{1}S_{0}\right)$ atomic fluorescence was recorded, displaying $\upsilon_{c}^{\prime }$ progression in the $c^{3}1$ ‘dark’ state.

### 6.2. Molecular Wave-Packet Interferometry with HgAr

**Figure 31:**
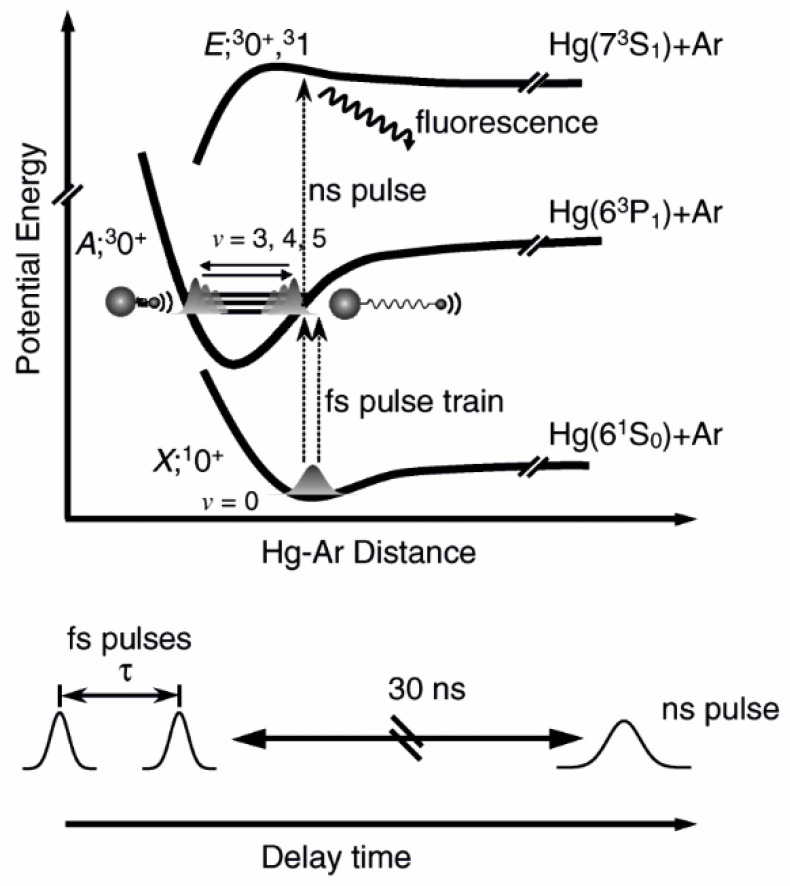
Pump control probe scheme for detecting the populations of the $A^{3}0^{+}$, $\upsilon_{A}^{\prime \prime }$ vibrational levels created by a double-laser pulse from the $X^{1}0^{+}$, $\upsilon_{X}=0$ vibrational level (from Ref. [217], under the permission of the American Physical Society).

Molecular wave-packet interferometry demonstrated with high-precision was reported by Ohmori and coworkers [217]. The wave packet was created in the $A^{3}0^{+}({6s6p\;}^{3}P_{1})$ state of the HgAr molecule using two time-delayed (τ) femtosecond (300 fs) pulses with a wavelength centred at 254.2 nm (see Figure 31; notation of molecular states after Ref. [217]). The centre wavelength of the pulses was selected so that the $\upsilon_{A}^{\prime \prime }=3,4,\;and\;5$ vibrational eigenstates of the $A^{3}0^{+}$ state were coherently superimposed, and two molecular wave-packets were created sequentially near the outer turning point of the $A^{3}0^{+}$-state PEC. A probe laser nanosecond pulse was delayed by 30 ns with respect to the femtosecond pulses, tuned to the $E^{3}1⟵A^{3}0^{+}$ transition, and used for LIF detection of the $A^{3}0^{+}$—state population. Because of its spectral bandwidth, the probe laser covered a small number of rotational lines ($J_{A}^{\prime \prime }=4–8$) with either $\upsilon_{E}^{\prime }=15⟵\upsilon_{A}^{\prime \prime }=3$, 16⟵4, or 17⟵5 vibrational bands of the $E^{3}1⟵A^{3}0^{+}$ transition. This allowed for observing the interferograms (with almost 100% fringe contrast) for the populations of the $\upsilon_{A}^{\prime \prime }=3,4,\;and\;5$ vibrational levels as a function of the τ delay time, which was tuned with sub-10 ns stability and resolution.

## 7. Conclusions

In this review, recent progress in studies of Rydberg double-well electronic energy states of 12-group CdNg (Ng = Ne, Ar, Kr) vdW molecules produced in molecular beams and investigated using techniques of laser spectroscopy such as OODR were presented and analysed.

**Figure 32:**
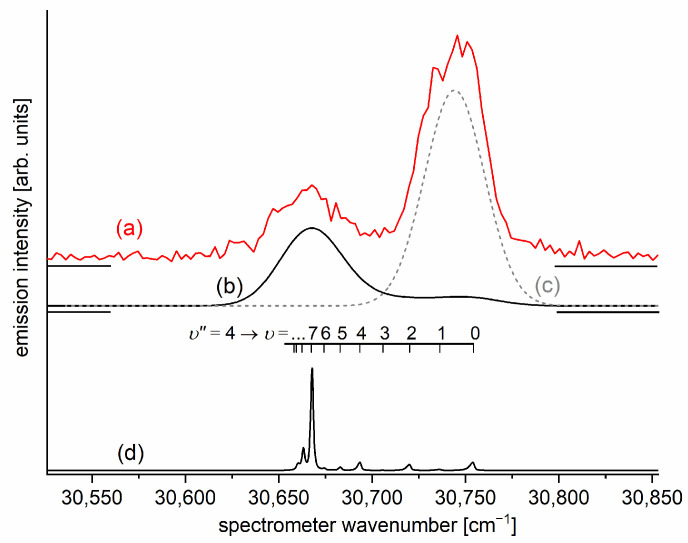
Dispersed emission spectrum corresponding to the $B^{3}1\left(5s{5p\;}^{3}P_{1}\right),\upsilon^{\prime \prime }=4\to \;X^{1}\Sigma_{0^{+}}\left({5s^{2}\;}^{1}S_{0}\right),\upsilon$ transitions in the CdAr molecule (**a**) recorded using a SpectraPro HRS 750 spectrometer (Teledyne Princeton Instruments) equipped with a CCD camera with an image intensifier (PIMAX 4) and diffraction grating with 1200 grooves/mm. (**b**) Simulation of the dispersed emission spectrum from (**a**) performed using the LEVEL [205] and PGOPHER [206] programs and assuming parameters of the $B^{3}1$ and $X^{1}\Sigma_{0^{+}}$-state potentials from Refs. [14,218], respectively, as well as Gaussian broadening responsible for spectrometer spectral throughput $\Delta_{G}=38\;cm^{-1}$. (**c**) Simulation of the recorded background signal associated with the excitation laser beam at the $B^{3}1,\upsilon^{\prime \prime }=4⟵X^{1}\Sigma_{0^{+}},\upsilon =0$ transition. (**d**) Simulated [205,206] distribution of transitions to different υ values in the $X^{1}\Sigma_{0^{+}}\;$ground state originating from $\upsilon^{\prime \prime }=4$ in the $B^{3}1$ state performed for $\Delta_{G}=1.9\;cm^{-1}$.

As a representative illustration, we quoted the progress that has been made to the present [10,12,13,14,15,19,202] as well as other supplemental results representing the most interesting advances in the $E^{3}\Sigma_{1}^{+}$ Rydberg state characterization in CdNg molecules, providing the following real added value to this review:

- Ab initio analysis of the formation of the outer well and the energy barrier in the $E^{3}\Sigma_{1}^{+}$ state of MeNg molecules presented in Section 1;
- Newly recorded experimental spectra, e.g., presented in Figure 11 ($E^{3}\Sigma_{1}^{+},\upsilon_{E}^{\prime }=0⟵A^{3}\Pi_{0^{+}},\upsilon_{A}^{\prime \prime }=0,1$ transition in CdNe) and Figure 22 ($E^{3}\Sigma_{1\;out}^{+},\upsilon_{E_{out}}^{\prime }⟵B^{3}1,\upsilon_{B}^{\prime \prime }=0$ transition in CdAr);
- New simulations of rotational profiles, e.g., presented in Figure 11 ($E^{3}\Sigma_{1}^{+},\upsilon_{E}^{\prime }=0⟵A^{3}\Pi_{0^{+}},\upsilon_{A}^{\prime \prime }=0,1$ transition in CdNe), Figure 17 ($E^{3}\Sigma_{1\;out}^{+},\upsilon_{E_{out}}^{\prime }⟵B^{3}1,\upsilon_{B}^{\prime \prime }$ transition in CdAr), and Figure 29 ($E^{3}\Sigma_{1\;in}^{+},\upsilon_{E_{in}}^{\prime }=0,3,18⟵A^{3}\Pi_{0^{+}},\upsilon_{A}^{\prime \prime }=9$ transitions in ^114^${Cd}^{86}Kr$);
- New analyses of experimental spectra, e.g., presented in Figure 19 ($E^{3}\Sigma_{1\;out}^{+},\upsilon_{E_{out}}^{\prime }⟵B^{3}1,\upsilon_{B}^{\prime \prime }=1–4$ transitions in CdAr) and Figure 20 ($E^{3}\Sigma_{1\;out}^{+},\upsilon_{E_{out}}^{\prime }⟵B^{3}1,\upsilon_{B}^{\prime \prime }=1,3$ transitions in CdAr);
- A preliminary study of dispersed emission spectra recorded using the $B^{3}1\left(5s{5p\;}^{3}P_{1}\right),\upsilon^{\prime \prime }=4\to \;X^{1}\Sigma_{0^{+}}\left(5s^{2\;1}S_{0}\right),\upsilon$ transitions in CdAr and their simulations presented in Figure 32 below.

Several examples of recent achievements and interesting approaches for rotational characterization, including direct bond-length determination in both potential wells, improved determination of potential representations for inner and outer potential wells, and the barrier that separates them, were described. This allowed for determining the complete Rydberg-state potential energy curves using recorded free⟵bound and bound⟵bound excitation spectra.

The concept of the so-called agreement plot and agreement parameter, employed for more comprehensive vibrational characterization of the energy structure supported by the electronic energy state as well as for the determination of the molecular bond length from modelling od distributions of F-C factors in excitation spectra, were also introduced.

As a practical approach, the isotopologue selection method for a rich isotopic and spectroscopically dense molecular energy structure using OODR was demonstrated as a useful method for considerable simplification of vibrational and rotational analyses.

Bound→free and bound→bound dispersed emission spectra after OODR excitation of a selected Rydberg state were presented via simulation as a perspective for characterization of the lower-lying ‘dark’ states or other electronic energy states inaccessible in a direct excitation from the ground state.

The presentation and analyses were performed in a broader context of the Rydberg states of MeNg and Me_2_ diatomic molecules, for which interatomic potentials possess complex (for example, double-well) structure, and including variety of spectroscopic methods of their investigations, such as laser vaporization–optical resonance and pump-and-probe methods or polarization labelling spectroscopy.

The importance of current state-of-the-art applications of Rydberg states with irregular potentials in photoassociation, vibrational and rotational cooling, molecular clocks, frequency standards, and molecular wave-packet interferometry was emphasized.

All spectroscopic characteristics of the $E^{3}\Sigma_{1}^{+}({5s6s\;}^{3}S_{1})$ Rydberg state in CdNe, CdAr, and CdKr determined in the presented studies of these molecules performed in the authors’ laboratory are collected in Table 1, where they are compared with experimental results of other studies and with the results of ab initio calculations. Suggestions for recommended values are also shown.

Tentative estimates of the errors in ab initio values of the $E^{3}\Sigma_{1}^{+}$-state inner potential well characteristics are shown in Table 1. Errors are extrapolated from the analyses presented in Ref. [3] for the two dominating sources of errors, i.e., incompleteness of the basis sets and deficiency in the description of the electron correlation. The latter factor appears to be the main source of errors. The errors due to the remaining factors were estimated by the magnitude of error due to the deficiency in the description of the electron correlation. The estimation of the errors of the characteristics of energy barrier and outer potential well needs further analysis that goes beyond the scope of this review. Similarly, the estimates of errors of the results of ab initio calculations performed for CdKr and presented in Ref. [208] need further analysis.

Considering future research plans on CdNg molecules, one has to postulate new ab initio calculations related to interatomic potentials of low-lying, non-Rydberg electronic energy states. It was indicated that deficiencies in the description of the electron correlation could be main source of the inaccuracies in ab initio calculations of excited states of MeNg molecules. Also, it was pointed out that future approaches of capturing electron correlation will most likely be based on EOM-CC methods, where the level of the approximation should go beyond the CCSD method. This would provide better theory-to-experiment agreement in future research not only for Rydberg but also lower-lying electronic states.

As far as experimental studies are concerned, it is necessary to point out that so far, they have mainly been based on the analysis of bound⟵bound and free⟵bound LIF excitation spectra. Future detection of both bound→bound and bound→free dispersed emission spectra can provide additional information about the shape of the PECs of the electronic energy states. In our laboratory, we plan to carry out experiments with detection of dispersed emission spectra of CdNg molecules, spectra that correspond to transitions originating from both non-Rydberg electronic states ($A^{3}\Pi_{0^{+}}$, $B^{3}1$) and low-lying Rydberg states ($E^{3}\Sigma_{1}^{+})$. Figure 32 presents preliminary results showing an example of the dispersed emission spectrum recorded for the CdAr molecule after laser excitation of the $\upsilon^{\prime \prime }=4$ level in the $B^{3}1$ state along with its simulation.

## Figures and Tables

**Table 1:** Spectroscopic characteristics of the $E^{3}\Sigma_{1}^{+}({5s6s\;}^{3}S_{1})$ state in CdNg (Ng = Ne, Ar, Kr) where characteristics obtained recently in Refs. [10,12,13,14,15,19,202], are supplemented/compared with those of analyses of this review, earlier studies [11,16,17,18,105], and results of recent ab initio calculations [4,39,40,207]. Recommended values are in bold. Note: $D_{e\;}^{\prime }$ is a rotational constant in contrast to $D_{e}^{\prime }$ well depth.

Designation	CdNe	CdAr		CdKr	
		$E^{3}\Sigma_{1\;in}^{+}$	$E^{3}\Sigma_{1\;out}^{+}$	$E^{3}\Sigma_{1\;in}^{+}$	$E^{3}\Sigma_{1\;out}^{+}$
$\omega_{e}^{\prime }\;({cm}^{-1})$	56.6 ± 3.0 [11]	**106.9** ± **0.2** [13] ^e^ 105.0 [105] 107.1 ± 2.0 [17] 105.4 [18] 106.5 ± 0.3 [16] 97.2 ± 11.0 [4] 93.5 ± 18.0 [39,40]	**4.36** ± **0.05** [12] ^e^ 4.4 ± 0.2 [17] 4.15 ± 0.05 [16] 4.15 ± 0.10 [15]	**107.36** ± **1.98** [19] ^e^ 91.0 ± 1.0 [20] 90.97 ± 1.00 [16] 91.1 ± 0.5 [14] ^m^	3.09 ± 0.14 [19] ^n^ 5.14 ± 0.23 [19] ^o^ 4.10 ± 0.15 [20]
$\omega_{e}^{\prime }x_{e}^{\prime }$ $\left({cm}^{-1}\right)$	8.8 ± 0.4 [11]	**2.052** ± **0.015** [13] ^e^ 2.21 [105] 2.1 ± 0.1 [17] 2.19 [18] 2.01 [4]	**0.207** ± **0.005** [12] ^e^ 0.20 ± 0.01 [17] 0.225 ± 0.005 [16] 0.225 ± 0.010 [15]	**1.626** ± **0.081** [19] ^e^ 1.25 ± 0.01 [20] 1.374 ± 0.030 [16] 1.42 ± 0.04 [14] ^m^	0.012 ± 0.012 [19] ^n^ 0.115 ± 0.009 [19] ^o^ 0.170 ± 0.008 [20]
$D_{e}^{\prime }$ $\left({cm}^{-1}\right)$	91.0 ± 4.0 [11]	**1260** ± **15** [13] ^f^ 1252.8 [105] 1309.5 ± 10.0 [17] 1266 [18] 1312.8 ± 14.2 [16] 1115 ± 230 [4] 1055 ± 380 [39,40]	**22.96** ± **0.76** [12] ^e^ 24.2 ± 1.0 [17] 19.14 ± 0.63 [16] 19.10 ± 1.30 [15] 16 [4] 17 [39,40]	**1772** ± **20** [19] ^e^ 1656.0 ± 3.0 [20] 1505.7 ± 1.0 [16] 1461.1 ± 9.0 [14] ^m^ 2056 [208] 1053 ± 505 [39,40]	**71** [19] 25.0 ± 2.0 [20] 27.0 ± 2.0 [20] ^k^ 38 [208] 16 [39,40]
$R_{e}^{\prime }$ (Å)	**2.98** ± **0.06** [10] ^b^ 3.21 ± 0.05 [11]	**2.850** ± **0.005** [16,17] **2.850** ± **0.005** [13] ^g^ 2.84 ± 0.03 [18,105] 2.88 ± 0.04 [4] 2.91 ± 0.06 [39,40]	**7.235** ± **0.121** [12] ^j^ 5.60 ± 0.05 [17] 7.63 ± 0.05 [16] 6.90 ± 0.15 [15] 7.356 [4] 7.673 [39,40]	2.99 ± 0.05 [16,20] 2.93 [208] 2.92 ± 0.10 [39,40]	5.90 ± 0.05 [20] 6.74 [208] 7.72 [39,40]
$B_{e}^{\prime }$ $\left({cm}^{-1}\right)$	**0.112** ± **0.002** [10] ^c^	0.07104 [18] 0.07016 ± 0.00246 [16]	0.00979 ± 0.00013 [16]	0.0385 ± 0.0013 [16]	**—**
$B_{\upsilon^{\prime }}\;({cm}^{-1})$	**0.106** ± **0.001** *_υ′_*_=0_ [10] **0.095** ± **0.001** *_υ′_*_=1_ [10] 0.092 *_υ′_*_=0_ [10] ^d^ 0.080 *_υ′_*_=1_ [10] ^d^	0.06754 *_υ__′_*_=0_ [13] ^d^ 0.06683 *_υ__′_*_=2_ [13] ^d^ 0.06360 *_υ__′_*_=5_ [13] ^d^ 0.06222 *_υ__′_*_=6_ [18] ^i^ 0.06066 *_υ__′_*_=7_ [18] ^i^ 0.05904 *_υ__′_*_=8_ [18] ^i^ 0.05738 *_υ′_*_=9_ [18] ^i^ 0.05565 *_υ′_*_=10_ [18] ^i^ 0.05543 *_υ′_*_=11_ [13] ^d^ 0.04132 *_υ′_*_=17_ [13] ^d^	**—**	0.03828 *_υ′_*_=0_ [14] ^l^ 0.03782 *_υ′_*_=1_ [14] ^l^ 0.03736 *_υ′_*_=2_ [14] ^l^ 0.03689 *_υ′_*_=3_ [14] ^l^	**—**
$D_{e\;}^{\prime }$ $\left({cm}^{-1}\right)$	**—**	(1.218 ± 0.128) $\times \;10^{-7}$ [16]	(2.18 ± 1.00) $\times \;10^{-7}$ [16]	(2.758 ± 0.319) $\times \;10^{-8}$ [16]	**—**
$D_{\upsilon^{\prime }}\;({cm}^{-1})$	**—**	1.172 $\times \;10^{-7}$ *_υ′_*_=0_ [13] ^d^ 1.495 $\times \;10^{-7}$ *_υ′_*_=2_ [13] ^d^ 1.654 $\times \;10^{-7}$ *_υ′_*_=5_ [13] ^d^ 2.499 $\times \;10^{-7}$ *_υ′_*_=11_ [13] ^d^ 7.289 $\times \;10^{-7}$ *_υ′_*_=17_ [13] ^d^	**—**	0.2980 $\times \;10^{-7}$ *_υ′_*_=0_ [14] ^l^ 0.3081 $\times \;10^{-7}$ *_υ′_*_=1_ [14] ^l^ 0.3189 $\times \;10^{-7}$ *_υ′_*_=2_ [14] ^l^ 0.3305 $\times \;10^{-7}$ *_υ′_*_=3_ [14] ^l^	**—**
$R_{b}^{\prime }$ (Å)	4.0 [11]	**5.15** [12] 4.7 [17] 4.964 [4] 4.890 [39,40]		4.63 [20] 5.46 [208] 4.90 [39,40]	
$E_{b}^{\prime }$ $\left({cm}^{-1}\right)$	(132–135) ± 4 [11] ^a^	**27** [12] 48.0 [17] ^a^ 21.1–39.4 [13] ^h^ 46 [4] 70 [39,40]		**22** [19] ^p^ (40–45) ± 10 [20] ^a^ 25 [208] ^p^ 70 [39,40] ^a^	

^a^ with respect to the $Cd\left({5s6s\;}^{3}S_{1}\right)$ asymptote and above the asymptote. ^b^ determined from $B_{\upsilon^{\prime }=0}$ and $B_{\upsilon^{\prime }=1}$. ^c^ from $B_{e}=(3B_{\upsilon^{\prime }=0}-B_{\upsilon^{\prime }=1})/2$. ^d^ using the LEVEL program [205] for ^116^${Cd}^{40}Ar$ and the $E^{3}\Sigma_{1}^{+}$-state characteristics. ^e^ from the linear B-S plot. ^f^ from $D_{0}^{\prime }+{\omega_{e}^{\prime }/2-\omega }_{e}^{\prime }x_{e}^{\prime }/4$, $D_{0}^{\prime }=\nu_{\upsilon^{\prime }=19\leftarrow \upsilon^{\prime \prime }=6}-\nu_{\upsilon^{\prime }=0\leftarrow \upsilon^{\prime \prime }=6}$. ^g^ estimated using the IPA method. ^h^ from $\nu_{\upsilon^{\prime }=0\leftarrow \upsilon^{\prime \prime }=6}+\nu_{\upsilon^{\prime }=19\leftarrow \upsilon^{\prime \prime }=6}-D_{0}\left(X^{1}\Sigma_{1}^{+}\right)-E_{at}\left({5s6s}^{3}S_{1}-{5s5p\;}^{3}P_{1}\right)$. ^i^ from analysis of the $e^{3}\Sigma_{1}^{+}\leftarrow b^{3}\Pi_{2}$ transition for ^116^${Cd}^{40}Ar$. ^j^ using the *agreement coefficient*, Equation (2). ^k^ from LRB analysis. ^l^ using the LEVEL program [205] for ^114^${Cd}^{86}Kr$ and $E^{3}\Sigma_{1}^{+}$-state characteristics. ^m^ using the agreement plot for ^114^${Cd}^{86}Kr$, Equation (3). ^n^ for $\upsilon_{E_{out}}^{\prime }=2–11$. ^o^ for $\upsilon_{E_{out}}^{\prime }=12–18$. ^p^ with respect to the $Cd\left({5s6s\;}^{3}S_{1}\right)$ asymptote and below the asymptote.

## Data Availability

No new data were created or analyzed in this study.
